# Supplementary material for: Increased Circulating CD14+ Monocytes in Patients with Psoriatic Arthritis Presenting Impaired Apoptosis Activity
Source: Biomedicines. 2024 Apr 1;12(4):775. doi: 10.3390/biomedicines12040775 (PMC11048590; doi:10.3390/biomedicines12040775)
Supplement: Supplementary file 1 [file biomedicines-12-00775-s001.zip › biomedicines-2894718-supplementary.pdf]

Supplementary Table S1. The primer sequences.

gene	sequence
IKBKB forward	CTCTGGAATCTCCTGAAGATTGCTTGTAG
IKBKB reverse	ACTCTTCTTGGCTGGCTCAGGTAA
CTSL forward	GCTGGTGGTTGGCTACGG
CTSL reverse	CTGGCTGCTGAGGCAATTCC
18S forward	GTAACCCGTTGAACCCCATT
18S ereverse	CCATCCAATCGGTAGTAGCG

Supplementary Table S2. The significantly (p-value<0.05) differentially expressed genes between HC and PsA samples.

Gene Symbol	p-value(PsA vs. HC)	Ratio(PsA vs. HC)	Fold-Change(PsA vs. HC)
RP11-1250I15.3	2.37E-05	0.849095	Down-regulated in PsA
MIR4529	7.16E-05	0.852082	Down-regulated in PsA
CTD-2046J7.1	8.50E-05	1.13452	Up-regulated in PsA
Metazoa_SRP	9.77E-05	1.40689	Up-regulated in PsA
CCM2	0.000138632	0.652289	Down-regulated in PsA
RP11-296L22.8	0.00016851	0.643956	Down-regulated in PsA
RN7SL32P	0.000186652	1.04918	Up-regulated in PsA
MR1	0.000190391	0.750151	Down-regulated in PsA
SERPINB8P1	0.000197135	1.30825	Up-regulated in PsA
RN7SL336P	0.000234964	1.02217	Up-regulated in PsA
AC005943.5	0.000252125	1.33327	Up-regulated in PsA
RPSAP19	0.000286829	0.847361	Down-regulated in PsA
FGF9	0.000287224	0.927472	Down-regulated in PsA
SAP30	0.000379988	0.728834	Down-regulated in PsA
AFF2	0.000381297	1.35121	Up-regulated in PsA
ZNF408	0.00038907	1.13381	Up-regulated in PsA
AP2A2	0.000467788	0.874084	Down-regulated in PsA
RP11-187C18.4	0.000491202	0.846995	Down-regulated in PsA
AC144836.1	0.000499784	1.44191	Up-regulated in PsA
HENMT1	0.000524287	0.655233	Down-regulated in PsA
RP11-463O9.2	0.000529401	1.28918	Up-regulated in PsA
GAS8	0.000553462	0.952534	Down-regulated in PsA

DMWD	0.000590159	0.816036	Down-regulated in PsA
RP11-25K19.1	0.00059694	1.21044	Up-regulated in PsA
TRAT1	0.000621351	1.14239	Up-regulated in PsA
RPL31P13	0.000645405	1.49662	Up-regulated in PsA
RP13-210D15.1	0.000648518	0.833492	Down-regulated in PsA
RP11-47G4.1	0.000649559	0.816868	Down-regulated in PsA
MIR575	0.000660723	1.3028	Up-regulated in PsA
SEPT14P8	0.000672576	0.722071	Down-regulated in PsA
FAM53C	0.000675196	1.14722	Up-regulated in PsA
SLC9A5	0.000698574	0.892672	Down-regulated in PsA
RP11-624L4.1	0.000714884	1.1813	Up-regulated in PsA
SNRPF	0.000739719	0.731	Down-regulated in PsA
RAB36	0.000752844	1.21569	Up-regulated in PsA
ZNRD1-AS1	0.00075875	0.523453	Down-regulated in PsA
GS1-466O4.5	0.000769065	0.672766	Down-regulated in PsA
LOC102467222	0.000786555	0.848018	Down-regulated in PsA
CTA-415G2.2	0.000808644	0.78895	Down-regulated in PsA
XXbac-BPGBPG34I8.2	0.000878839	1.45339	Up-regulated in PsA
RN7SL679P	0.000935574	1.27347	Up-regulated in PsA
RP11-525K10.2	0.00093749	1.28064	Up-regulated in PsA
NR2E1	0.000957441	1.14396	Up-regulated in PsA
SEC62-AS1	0.000960994	1.17614	Up-regulated in PsA
TRPM1	0.000961237	0.856917	Down-regulated in PsA
AC002400.1	0.00104132	0.774939	Down-regulated in PsA
SNORD74	0.00107904	1.31914	Up-regulated in PsA

APOBR	0.00111514	0.770995	Down-regulated in PsA
LOC401585	0.00112606	1.15786	Up-regulated in PsA
RNY1	0.00114947	1.07862	Up-regulated in PsA
RP11-159D12.8	0.00123031	0.727212	Down-regulated in PsA
ATP13A4	0.00125105	1.32945	Up-regulated in PsA
MED4	0.0012511	0.8265	Down-regulated in PsA
RP11-132M7.2	0.00127915	1.619	Up-regulated in PsA
NMNAT1P4	0.0012866	0.774346	Down-regulated in PsA
HERC2P4	0.0012932	0.485075	Down-regulated in PsA
RAD51AP2	0.00129942	0.64071	Down-regulated in PsA
HMGB1P47	0.00132219	1.21196	Up-regulated in PsA
RP11-339N8.1	0.00135137	0.814475	Down-regulated in PsA
GABBR1	0.00138556	0.765234	Down-regulated in PsA
RP11-561N12.6	0.0013925	0.826986	Down-regulated in PsA
PAFAH1B1	0.00139348	0.81306	Down-regulated in PsA
ZNF354B	0.00139818	0.772608	Down-regulated in PsA
CRPP1	0.00142678	0.80707	Down-regulated in PsA
EIF4EBP2P1	0.00145529	1.3221	Up-regulated in PsA
RP11-344N17.3	0.0015087	0.906627	Down-regulated in PsA
MIR526A2	0.00152026	0.76561	Down-regulated in PsA
RN7SL143P	0.00152524	1.30417	Up-regulated in PsA
MUS81	0.00153708	0.843145	Down-regulated in PsA
PRKCA-AS1	0.00153898	1.28617	Up-regulated in PsA
RP5-1142J19.2	0.00154122	1.37129	Up-regulated in PsA
NCAM2	0.00154883	0.832106	Down-regulated in PsA

ZNF529-AS1	0.00156011	0.89338	Down-regulated in PsA
WWC2	0.00156703	0.77491	Down-regulated in PsA
STARD5	0.00157963	1.11423	Up-regulated in PsA
E4F1	0.00159106	1.02397	Up-regulated in PsA
ZNRD1-AS1_2	0.00159355	0.903781	Down-regulated in PsA
RP11-791O21.5	0.00161576	1.18544	Up-regulated in PsA
LOC100653233	0.00162109	1.68109	Up-regulated in PsA
RP11-22A3.2	0.00165188	1.14784	Up-regulated in PsA
RP11-497J7.4	0.00171121	1.0779	Up-regulated in PsA
CIART	0.00171504	0.76399	Down-regulated in PsA
HAUS3	0.00172091	0.713264	Down-regulated in PsA
RP11-341D18.2	0.00172892	1.36082	Up-regulated in PsA
INIP	0.00174577	0.798018	Down-regulated in PsA
SNAR-A12	0.00178368	1.15196	Up-regulated in PsA
SNAR-A12	0.00178368	1.15196	Up-regulated in PsA
ODC1	0.00178536	0.858466	Down-regulated in PsA
LOC105375556	0.0017926	0.819601	Down-regulated in PsA
RASD1	0.00183187	1.1485	Up-regulated in PsA
TOP3B	0.00184204	0.773297	Down-regulated in PsA
MIR4526	0.00184753	1.23005	Up-regulated in PsA
RP11-594C13.2	0.00186159	0.736686	Down-regulated in PsA
GAPDHP67	0.00186321	0.856196	Down-regulated in PsA
LOC339803	0.00188497	1.09246	Up-regulated in PsA
PCBD2	0.00189097	0.719388	Down-regulated in PsA
CTD-2008P7.1	0.00190436	0.829623	Down-regulated in PsA

MIR185	0.00190999	1.17352	Up-regulated in PsA
SLC25A10	0.00191206	1.19034	Up-regulated in PsA
RP11-963H4.6	0.00192238	1.33855	Up-regulated in PsA
AC090957.2	0.00195133	1.12174	Up-regulated in PsA
RP11-553K8.2	0.00196529	1.36273	Up-regulated in PsA
RP11-369K16.1	0.00197949	0.528255	Down-regulated in PsA
LOC101927488	0.00199046	0.88184	Down-regulated in PsA
FOXN3-AS1	0.0020203	0.730273	Down-regulated in PsA
SARS	0.00203359	0.844009	Down-regulated in PsA
TRPV4	0.00206361	0.650063	Down-regulated in PsA
RP11-684B21.1	0.00208275	1.33798	Up-regulated in PsA
RP11-65E10.1	0.0020914	1.84909	Up-regulated in PsA
AC006129.1	0.00210851	1.14746	Up-regulated in PsA
RP11-48B3.3	0.0021164	1.15804	Up-regulated in PsA
MGA	0.00213153	0.699174	Down-regulated in PsA
OSTC	0.00214325	0.806597	Down-regulated in PsA
RNU6-129P	0.00214526	1.37997	Up-regulated in PsA
L29074.3	0.0021462	0.892528	Down-regulated in PsA
RP11-1112C15.2	0.00216375	0.858329	Down-regulated in PsA
RP11-485B17.3	0.00217026	1.16139	Up-regulated in PsA
SFRP5	0.00220237	1.45525	Up-regulated in PsA
SCRG1	0.00220414	0.726525	Down-regulated in PsA
TRIM6-TRIM34	0.00220676	0.596184	Down-regulated in PsA
COL4A1	0.0022109	1.34186	Up-regulated in PsA
TBCD	0.00223584	0.905093	Down-regulated in PsA

RP11-460I13.2	0.00223777	1.10834	Up-regulated in PsA
ABT1P1	0.00224491	1.19451	Up-regulated in PsA
ATXN3L	0.00224512	0.828166	Down-regulated in PsA
RP11-341D18.6	0.00224619	0.911294	Down-regulated in PsA
GAS2L1P1	0.00225826	0.875587	Down-regulated in PsA
LOC728673	0.00227901	1.3815	Up-regulated in PsA
PADI2	0.00227902	0.505285	Down-regulated in PsA
PTCHD3P2	0.00231908	0.755604	Down-regulated in PsA
RP11-417J1.1	0.00234347	0.885868	Down-regulated in PsA
TNPO1P2	0.00235542	0.652873	Down-regulated in PsA
RP11-95G6.1	0.00240088	1.14529	Up-regulated in PsA
SPANXA2-OT1	0.00242616	1.17192	Up-regulated in PsA
S100A4	0.00245338	0.941235	Down-regulated in PsA
DPH6	0.00246262	0.638028	Down-regulated in PsA
RP11-295P9.8	0.00246832	0.903634	Down-regulated in PsA
MUTYH	0.00246933	1.13437	Up-regulated in PsA
TDRD12	0.00248528	0.73348	Down-regulated in PsA
KRT43P	0.00248782	1.6596	Up-regulated in PsA
ZEB1-AS1	0.00249932	1.14993	Up-regulated in PsA
CST1	0.00249953	0.642655	Down-regulated in PsA
SERPINB5	0.00250835	0.905523	Down-regulated in PsA
MIR4473	0.00251442	0.845493	Down-regulated in PsA
DAPK3	0.00252426	0.677331	Down-regulated in PsA
RP11-155I9.1	0.00254579	0.773374	Down-regulated in PsA
PFDN4	0.00254612	0.716175	Down-regulated in PsA

RPS26P30	0.00254771	0.895834	Down-regulated in PsA
AC099850.1	0.00258976	1.20673	Up-regulated in PsA
LINC01164	0.00260623	1.46895	Up-regulated in PsA
TPT1P7	0.00262174	1.13061	Up-regulated in PsA
HSPD1P15	0.00262727	0.768833	Down-regulated in PsA
SEPT14P24	0.00265262	0.767683	Down-regulated in PsA
NDUFA9	0.00265777	0.795713	Down-regulated in PsA
RER1	0.0026781	0.738385	Down-regulated in PsA
UTS2	0.002705	0.0756545	Down-regulated in PsA
TNFRSF11B	0.002738	1.17584	Up-regulated in PsA
NUS1P4	0.00274304	0.819097	Down-regulated in PsA
RP1-229K20.5	0.0027863	1.28903	Up-regulated in PsA
COA1	0.00283402	1.21282	Up-regulated in PsA
AC079630.4	0.00283408	0.899963	Down-regulated in PsA
SCARNA6	0.00284538	0.964833	Down-regulated in PsA
SCARNA6	0.00284538	0.964833	Down-regulated in PsA
CTD-2140B24.6	0.00286076	1.49012	Up-regulated in PsA
LINC-ROR	0.00287027	0.819535	Down-regulated in PsA
ERV3-1	0.00291675	0.549128	Down-regulated in PsA
CTD-2073O6.1	0.00293916	1.14276	Up-regulated in PsA
GDPGP1	0.0029466	0.64306	Down-regulated in PsA
SETP11	0.00300706	1.20961	Up-regulated in PsA
RP11-328L11.1	0.00300813	1.10549	Up-regulated in PsA
FAM216A	0.00302729	0.761242	Down-regulated in PsA
RN7SKP178	0.00303302	0.73638	Down-regulated in PsA

RP11-319G9.4	0.00304267	1.40115	Up-regulated in PsA
ACTR1A	0.00305609	0.935546	Down-regulated in PsA
RP11-506H20.2	0.00306726	2.08243	Up-regulated in PsA
MIA	0.00307125	1.15328	Up-regulated in PsA
GS1-251I9.3	0.00308504	1.0445	Up-regulated in PsA
RP11-7C6.1	0.0031035	1.44143	Up-regulated in PsA
RP11-110A12.2	0.00315054	0.850289	Down-regulated in PsA
KRTAP5-2	0.00318865	1.28341	Up-regulated in PsA
RP11-380D23.1	0.00321748	1.31556	Up-regulated in PsA
RP11-540B6.3	0.00322121	0.686639	Down-regulated in PsA
LMO4	0.00326811	0.800057	Down-regulated in PsA
AC009120.3	0.00329156	0.815601	Down-regulated in PsA
ZNF37A	0.00330081	0.711254	Down-regulated in PsA
AC012671.2	0.00333413	1.14702	Up-regulated in PsA
CTC-505O3.3	0.00333558	0.736855	Down-regulated in PsA
DHRS4	0.00335418	0.697931	Down-regulated in PsA
TTC7B	0.0033821	0.863209	Down-regulated in PsA
RP11-498E2.7	0.00340056	0.909108	Down-regulated in PsA
DNAJC5B	0.00340484	0.636965	Down-regulated in PsA
SLC16A1	0.00343672	0.61593	Down-regulated in PsA
OR10J3	0.00347406	0.807802	Down-regulated in PsA
RP11-656D10.6	0.00350096	1.21533	Up-regulated in PsA
GSTZ1	0.00350855	0.858688	Down-regulated in PsA
CYP2D6	0.00351064	1.16694	Up-regulated in PsA
LINC00910	0.00352302	1.1248	Up-regulated in PsA

NCALD	0.00353343	1.21617	Up-regulated in PsA
EMC6	0.00354397	0.948797	Down-regulated in PsA
RN7SL271P	0.00355242	1.09255	Up-regulated in PsA
RN7SL426P	0.0035645	1.08685	Up-regulated in PsA
FADD	0.00357763	0.803587	Down-regulated in PsA
PARG	0.00362911	0.715398	Down-regulated in PsA
LRRC1	0.0036561	0.588371	Down-regulated in PsA
LOC283194	0.00369756	0.743497	Down-regulated in PsA
RNASEH1	0.00374252	1.21212	Up-regulated in PsA
MIR6871	0.00375236	1.25936	Up-regulated in PsA
OR1L4	0.00377919	0.729836	Down-regulated in PsA
ATP10A	0.00383424	0.788678	Down-regulated in PsA
C5orf66-AS2	0.00388041	1.33384	Up-regulated in PsA
LOC105755953	0.00388824	0.733734	Down-regulated in PsA
LINC00871	0.00389759	0.782372	Down-regulated in PsA
RP11-257P3.3	0.00393682	1.48002	Up-regulated in PsA
RP11-74E22.5	0.00394805	0.671788	Down-regulated in PsA
RP11-116O18.3	0.00395799	1.16359	Up-regulated in PsA
ZNF416	0.00399014	0.657003	Down-regulated in PsA
LOC105378405	0.00404002	0.806765	Down-regulated in PsA
ARL14	0.00405189	0.785451	Down-regulated in PsA
RP11-21B21.4	0.00407211	0.758047	Down-regulated in PsA
WISP2	0.00408051	1.09555	Up-regulated in PsA
LINC00686	0.00410882	1.11831	Up-regulated in PsA
CNTD1	0.00410961	0.599738	Down-regulated in PsA

DCLRE1CP1	0.00411346	0.675854	Down-regulated in PsA
RNASE3	0.00412125	0.53984	Down-regulated in PsA
RN7SKP124	0.00413348	1.28027	Up-regulated in PsA
MYO16	0.00413565	0.858195	Down-regulated in PsA
FAM166B	0.00414421	0.750668	Down-regulated in PsA
CECR3	0.00415132	1.11422	Up-regulated in PsA
RYBP	0.00415809	0.777539	Down-regulated in PsA
AC007274.2	0.00417095	1.18362	Up-regulated in PsA
SLX4	0.00420109	0.764217	Down-regulated in PsA
RP11-141J13.3	0.00421486	1.28601	Up-regulated in PsA
CTB-31O20.9	0.00423711	1.40283	Up-regulated in PsA
RP11-149F8.4	0.00423944	1.57165	Up-regulated in PsA
RP11-160K17.1	0.00425917	1.68065	Up-regulated in PsA
MIR4660	0.00425926	0.702459	Down-regulated in PsA
SDR42E1P3	0.00426188	1.15878	Up-regulated in PsA
FRG2DP	0.00426626	1.34858	Up-regulated in PsA
RP11-136I14.2	0.00428654	0.556263	Down-regulated in PsA
EIF4EP5	0.00429041	0.735852	Down-regulated in PsA
CTNNBIP1	0.00432032	0.971721	Down-regulated in PsA
MIR4438	0.00432458	0.794243	Down-regulated in PsA
DCAF10	0.00432743	0.717625	Down-regulated in PsA
RPS12	0.004377	1.04855	Up-regulated in PsA
DNAJC3-AS1	0.00440964	0.854969	Down-regulated in PsA
NCAPG2	0.00444087	0.694894	Down-regulated in PsA
RP1-293L6.1	0.00446686	0.880661	Down-regulated in PsA

DCDC5	0.00447122	1.2984	Up-regulated in PsA
CTD-2326C4.1	0.00452074	0.893583	Down-regulated in PsA
KCNMB2-AS1	0.0045243	1.27477	Up-regulated in PsA
AC072061.2	0.00452518	1.39015	Up-regulated in PsA
RP3-406A7.7	0.00456223	1.59587	Up-regulated in PsA
CH507-236L23.2	0.00457291	0.864762	Down-regulated in PsA
RP11-184M15.2	0.0046194	1.94118	Up-regulated in PsA
ZCCHC7	0.00463584	0.769934	Down-regulated in PsA
RP11-321A17.5	0.0046494	1.34882	Up-regulated in PsA
SNORD96B	0.00466571	0.756276	Down-regulated in PsA
CCL1	0.00468728	1.14494	Up-regulated in PsA
C9orf153	0.00470691	0.688649	Down-regulated in PsA
SUDS3P1	0.00470879	1.22618	Up-regulated in PsA
SPATA1	0.00472009	0.603738	Down-regulated in PsA
RP11-334C17.3	0.00472829	1.46256	Up-regulated in PsA
RP11-495P10.4	0.00473981	1.38565	Up-regulated in PsA
HSPD1	0.00475232	0.726778	Down-regulated in PsA
SKIV2L2	0.00475541	0.759377	Down-regulated in PsA
AP000997.3	0.00480097	1.36401	Up-regulated in PsA
FRAT2	0.00481767	0.808028	Down-regulated in PsA
N4BP1	0.00482278	0.8006	Down-regulated in PsA
NDUFS5P1	0.00482359	1.30486	Up-regulated in PsA
CHRM3	0.00483858	0.842188	Down-regulated in PsA
CTC-563A5.2	0.00484796	1.36219	Up-regulated in PsA
LDLRAP1	0.00487077	1.07088	Up-regulated in PsA

RP11-541M12.3	0.00488496	0.723912	Down-regulated in PsA
CRLS1	0.00488721	0.854033	Down-regulated in PsA
AC116050.1	0.00492068	1.23103	Up-regulated in PsA
RP11-12A20.8	0.00494205	1.51236	Up-regulated in PsA
CPLX2	0.00496012	1.16175	Up-regulated in PsA
MTCO1P27	0.00497427	0.847112	Down-regulated in PsA
EFNA2	0.00498569	1.12998	Up-regulated in PsA
RP11-261C10.1	0.00499573	1.1722	Up-regulated in PsA
ANGPT2	0.00503224	0.727173	Down-regulated in PsA
EIF3J	0.00507148	0.721147	Down-regulated in PsA
ASB4	0.00507336	0.652761	Down-regulated in PsA
BCS1L	0.0050736	0.850477	Down-regulated in PsA
PRELID3BP8	0.0050826	1.20175	Up-regulated in PsA
RAB3IP	0.00508628	0.666303	Down-regulated in PsA
PPARG	0.0050969	0.66373	Down-regulated in PsA
RP11-675F6.4	0.00512486	1.18438	Up-regulated in PsA
LOC101929380	0.00513246	1.1531	Up-regulated in PsA
RP3-508I15.9	0.00516896	1.43841	Up-regulated in PsA
INAFM1	0.00518649	1.15023	Up-regulated in PsA
LOC101929229	0.00518975	0.802629	Down-regulated in PsA
ACD	0.00520317	0.957171	Down-regulated in PsA
RNU7-127P	0.00521904	0.946926	Down-regulated in PsA
ZNF652	0.00522751	0.86364	Down-regulated in PsA
VPS8	0.00524515	0.654255	Down-regulated in PsA
RP11-43N22.1	0.00525257	1.28672	Up-regulated in PsA

RP11-573G6.8	0.00525898	1.37994	Up-regulated in PsA
RBPJP7	0.00527112	0.782031	Down-regulated in PsA
RP11-615I2.1	0.00529453	1.0405	Up-regulated in PsA
Y_RNA	0.00529605	1.26903	Up-regulated in PsA
RP4-700A9.1	0.00530483	1.18213	Up-regulated in PsA
CTD-2574D22.4	0.00530916	0.705094	Down-regulated in PsA
DEFB121	0.00534962	0.723061	Down-regulated in PsA
BUD13	0.00535294	0.80032	Down-regulated in PsA
LOC375196	0.00535464	0.84649	Down-regulated in PsA
IPO5P1	0.00538708	1.4073	Up-regulated in PsA
ANP32AP1	0.00540384	0.725111	Down-regulated in PsA
FAM187B2P	0.00542706	1.45972	Up-regulated in PsA
C3orf36	0.00543893	1.13802	Up-regulated in PsA
ZNF35	0.00545505	0.661146	Down-regulated in PsA
PRLHR	0.0054785	0.749183	Down-regulated in PsA
CASP4	0.00551814	0.844179	Down-regulated in PsA
VPS41	0.00554984	0.812942	Down-regulated in PsA
PSMA4	0.00555456	0.792666	Down-regulated in PsA
RN7SL478P	0.00557039	1.17022	Up-regulated in PsA
RP11-295D4.3	0.00557781	1.27365	Up-regulated in PsA
RP11-770G2.5	0.0055896	0.923485	Down-regulated in PsA
RP11-546K22.1	0.00562181	0.843081	Down-regulated in PsA
AC067969.2	0.00562408	1.13279	Up-regulated in PsA
RP11-25I15.1	0.00573407	1.19101	Up-regulated in PsA
SQRDL	0.00579198	0.93359	Down-regulated in PsA

USP20	0.00579519	0.823787	Down-regulated in PsA
PAGE2	0.00583178	0.74928	Down-regulated in PsA
MIR519A2	0.00584976	1.50033	Up-regulated in PsA
FCHSD1	0.00586749	0.834644	Down-regulated in PsA
RP11-54P19.1	0.00590369	1.18392	Up-regulated in PsA
RP1-209B5.2	0.00591842	0.74783	Down-regulated in PsA
OR4X1	0.00597582	0.813801	Down-regulated in PsA
ISM1-AS1	0.00603712	1.27374	Up-regulated in PsA
MIR500B	0.00604299	0.925764	Down-regulated in PsA
OTUD6B-AS1	0.00605562	0.664448	Down-regulated in PsA
ZHX1-C8orf76	0.00605965	0.820116	Down-regulated in PsA
AC027124.3	0.00606797	1.22441	Up-regulated in PsA
GALNT11	0.00607955	0.752496	Down-regulated in PsA
RP11-754I20.3	0.00608033	1.28594	Up-regulated in PsA
RP3-334F4.2	0.00608927	0.883886	Down-regulated in PsA
BPI	0.0061322	0.560751	Down-regulated in PsA
ZNF30	0.0061327	1.21466	Up-regulated in PsA
RP6-43L17.2	0.00615906	1.19947	Up-regulated in PsA
KLF2P4	0.00621865	1.28213	Up-regulated in PsA
RP11-298J23.8	0.00627122	1.60688	Up-regulated in PsA
LOC102724776	0.00628333	1.05654	Up-regulated in PsA
MYC	0.00637983	0.656943	Down-regulated in PsA
NFKB1	0.00639143	0.777525	Down-regulated in PsA
ABI1	0.00640495	0.838628	Down-regulated in PsA
MIR15B	0.0064208	0.632928	Down-regulated in PsA

OR7C2	0.00644177	0.685007	Down-regulated in PsA
RP11-119F19.5	0.00644718	0.658865	Down-regulated in PsA
LGI2	0.00646744	0.767121	Down-regulated in PsA
GPR21	0.00646896	0.636485	Down-regulated in PsA
KB-1073A2.1	0.00647753	0.76761	Down-regulated in PsA
PSMG3	0.00654107	1.18457	Up-regulated in PsA
MRPL19	0.00655923	0.780393	Down-regulated in PsA
ATG16L1	0.00657083	0.796327	Down-regulated in PsA
DEDD	0.00661664	0.885878	Down-regulated in PsA
CASP7	0.00662208	0.918258	Down-regulated in PsA
MYDGF	0.00663738	0.861432	Down-regulated in PsA
RP11-247I13.3	0.00666912	1.15751	Up-regulated in PsA
pRNA	0.00668096	1.29658	Up-regulated in PsA
AC083843.2	0.0066843	0.91512	Down-regulated in PsA
COLGALT1	0.00668998	0.864835	Down-regulated in PsA
MRPL3	0.00671108	0.600815	Down-regulated in PsA
AC008264.4	0.00672353	0.930355	Down-regulated in PsA
RP11-109G23.1	0.00675894	1.1208	Up-regulated in PsA
TMEM191A	0.00676967	1.70745	Up-regulated in PsA
FAM138A	0.00677039	1.27234	Up-regulated in PsA
RP11-517A5.5	0.00677436	0.689605	Down-regulated in PsA
RP11-180N14.1	0.00678162	1.13139	Up-regulated in PsA
RP11-394O4.3	0.00678991	1.46251	Up-regulated in PsA
ALDOC	0.00681328	1.23164	Up-regulated in PsA
UBTD2	0.0068179	0.726476	Down-regulated in PsA

SART1	0.00681975	0.942469	Down-regulated in PsA
RP11-773H22.4	0.00684109	0.78577	Down-regulated in PsA
RP11-207C16.4	0.00688563	0.899984	Down-regulated in PsA
LOC101929512	0.00689218	1.11059	Up-regulated in PsA
BORCS5	0.00690713	1.13577	Up-regulated in PsA
PRR27	0.00691333	0.780799	Down-regulated in PsA
RPSAP11	0.00693384	0.905925	Down-regulated in PsA
RP5-933B4.1	0.00700519	1.2463	Up-regulated in PsA
LINC00265	0.00704248	1.11097	Up-regulated in PsA
FAM134A	0.00705194	0.782394	Down-regulated in PsA
RN7SL172P	0.00705772	1.0535	Up-regulated in PsA
RP11-44L9.2	0.00706018	0.858814	Down-regulated in PsA
RP11-641A6.5	0.00706095	0.82385	Down-regulated in PsA
KLF17	0.00706521	1.06563	Up-regulated in PsA
MIR7975	0.00707078	1.27506	Up-regulated in PsA
IGFL3	0.00708221	0.880189	Down-regulated in PsA
FOXP1-IT1	0.00708429	0.414445	Down-regulated in PsA
FCF1P8	0.00709316	0.656781	Down-regulated in PsA
OXSM	0.00712667	0.860758	Down-regulated in PsA
COX15	0.00714549	0.720769	Down-regulated in PsA
MIR4489	0.00715228	1.20623	Up-regulated in PsA
PDCL3P3	0.00720557	1.38562	Up-regulated in PsA
ACAD10	0.00722612	0.751417	Down-regulated in PsA
AP000568.2	0.00722918	0.855874	Down-regulated in PsA
EYS	0.00723079	0.743966	Down-regulated in PsA

MIR6893	0.00725357	1.21992	Up-regulated in PsA
SERTAD4	0.00726763	1.17045	Up-regulated in PsA
DLL4	0.00727035	0.731345	Down-regulated in PsA
SAE1	0.00728154	0.855829	Down-regulated in PsA
LOC644172	0.00731909	1.45265	Up-regulated in PsA
RP11-492D6.3	0.00732515	1.426	Up-regulated in PsA
B4GALT3	0.00732809	0.823075	Down-regulated in PsA
RNU6-335P	0.00732987	1.28174	Up-regulated in PsA
RP11-406O23.2	0.00733767	1.08122	Up-regulated in PsA
USP17L2	0.00733842	1.29633	Up-regulated in PsA
UROD	0.00734919	0.846176	Down-regulated in PsA
RPSAP50	0.00735321	1.07795	Up-regulated in PsA
RCAN3AS	0.00736079	0.698701	Down-regulated in PsA
BEX4	0.00738477	0.53521	Down-regulated in PsA
RP11-692E14.1	0.00745898	0.795922	Down-regulated in PsA
RP11-775J23.2	0.00746231	0.754538	Down-regulated in PsA
RP11-429K17.1	0.00747537	1.09805	Up-regulated in PsA
RP11-321E2.8	0.00748495	1.37775	Up-regulated in PsA
RPS4XP6	0.00749694	1.13986	Up-regulated in PsA
RP11-803B1.8	0.00752195	1.31841	Up-regulated in PsA
XPC	0.00752382	0.751593	Down-regulated in PsA
MAPK10	0.00752709	0.935478	Down-regulated in PsA
LOC101929696	0.00754991	1.42786	Up-regulated in PsA
BIRC2	0.00755812	0.738397	Down-regulated in PsA
NUMB	0.00756741	0.911542	Down-regulated in PsA

PCDHB2	0.00760125	1.44554	Up-regulated in PsA
METTL20	0.00761144	0.736846	Down-regulated in PsA
RP11-204M4.1	0.00762856	1.56924	Up-regulated in PsA
SHROOM1	0.00764417	0.490798	Down-regulated in PsA
PRDX3P4	0.00764601	0.860692	Down-regulated in PsA
AP000697.6	0.00765304	1.21097	Up-regulated in PsA
TMEM9B	0.00767462	0.853226	Down-regulated in PsA
MIR4461	0.00767983	1.14185	Up-regulated in PsA
LSG1	0.00771706	0.768655	Down-regulated in PsA
RP11-422J15.1	0.00772608	1.20003	Up-regulated in PsA
RP11-490G8.1	0.00772652	1.25113	Up-regulated in PsA
TMEM99	0.0077396	0.610421	Down-regulated in PsA
LOC102723854	0.00778705	1.09136	Up-regulated in PsA
HSPE1P19	0.00779574	0.982937	Down-regulated in PsA
SARNP	0.00782535	0.850169	Down-regulated in PsA
KPNA7	0.00785567	0.728853	Down-regulated in PsA
LOC101929633	0.00789925	0.668527	Down-regulated in PsA
KANK3	0.00791355	1.21322	Up-regulated in PsA
RN7SKP133	0.00793193	1.15502	Up-regulated in PsA
RP11-312J18.5	0.00795553	1.17548	Up-regulated in PsA
YARS	0.00798152	0.884192	Down-regulated in PsA
RP11-186N15.3	0.00799624	1.2873	Up-regulated in PsA
CRKL	0.00801309	0.92916	Down-regulated in PsA
IKBKE	0.00803629	0.800358	Down-regulated in PsA
MIR6774	0.00805456	1.11864	Up-regulated in PsA

RP5-916L7.2	0.00809178	1.32248	Up-regulated in PsA
PRRX2-AS1	0.00813795	1.14267	Up-regulated in PsA
MNX1-AS2	0.00816562	1.22051	Up-regulated in PsA
Metazoa_SRP	0.00818901	0.949015	Down-regulated in PsA
NIFK	0.00819847	0.839695	Down-regulated in PsA
RP11-216P16.6	0.00824565	1.48255	Up-regulated in PsA
MTF1	0.00825209	0.80284	Down-regulated in PsA
FABP3	0.00826929	1.28844	Up-regulated in PsA
SMCR2	0.00827252	1.52065	Up-regulated in PsA
RP5-981O7.2	0.00831552	0.712022	Down-regulated in PsA
RAX	0.00832233	1.25184	Up-regulated in PsA
TRIM36	0.0083521	0.822854	Down-regulated in PsA
CCDC107	0.00835467	0.798112	Down-regulated in PsA
VPS11	0.00837109	1.20605	Up-regulated in PsA
OR6N2	0.00837431	0.697704	Down-regulated in PsA
DNHD1	0.0083943	0.80938	Down-regulated in PsA
XRCC2	0.00840349	0.645581	Down-regulated in PsA
AXIN2	0.00841021	0.689425	Down-regulated in PsA
PCDH18	0.00842244	1.13039	Up-regulated in PsA
RNU7-190P	0.00842417	0.594434	Down-regulated in PsA
LOC101928569	0.00844103	1.26387	Up-regulated in PsA
RP11-493P1.2	0.00845137	0.693564	Down-regulated in PsA
ZIC5	0.00847535	0.831505	Down-regulated in PsA
ACBD3	0.00847997	0.829023	Down-regulated in PsA
RP11-357N13.6	0.00849045	0.650666	Down-regulated in PsA

SEMG1	0.00851587	0.6701	Down-regulated in PsA
C2orf78	0.00851785	1.21973	Up-regulated in PsA
AC005009.2	0.00852862	2.27665	Up-regulated in PsA
SLC29A2	0.00853014	0.892759	Down-regulated in PsA
CTC-459F4.6	0.00855946	0.698069	Down-regulated in PsA
TMEM44-AS1	0.00856919	0.581583	Down-regulated in PsA
BRCC3	0.00862232	0.770874	Down-regulated in PsA
LOC101929125	0.0086246	0.837294	Down-regulated in PsA
KRT8P8	0.00871791	1.40995	Up-regulated in PsA
ABCA17P	0.00872431	1.07881	Up-regulated in PsA
DOCK9-AS2	0.008757	1.46846	Up-regulated in PsA
AF240627.2	0.00876563	1.29193	Up-regulated in PsA
C18orf54	0.00879043	0.556558	Down-regulated in PsA
PDK4	0.0087989	0.73095	Down-regulated in PsA
LYPD5	0.0088082	0.869058	Down-regulated in PsA
DDX53	0.00884087	1.1882	Up-regulated in PsA
CTC-340I23.2	0.00884451	0.84293	Down-regulated in PsA
AC145343.2	0.0088662	1.44383	Up-regulated in PsA
MECOM	0.00889583	0.74019	Down-regulated in PsA
ST20	0.00890773	0.971548	Down-regulated in PsA
SMIM10	0.00891778	1.36499	Up-regulated in PsA
CTD-2616J11.15	0.00892559	1.08837	Up-regulated in PsA
OR8D1	0.00894648	1.19634	Up-regulated in PsA
RP11-282K24.3	0.0089578	0.753175	Down-regulated in PsA
SMIM23	0.0089814	0.84615	Down-regulated in PsA

FAR1P1	0.00898805	0.72127	Down-regulated in PsA
LGI3	0.00899095	0.786929	Down-regulated in PsA
SNORD113-8	0.00900266	1.29444	Up-regulated in PsA
DLX6-AS1	0.00901969	0.819304	Down-regulated in PsA
SLC6A12	0.00903048	0.731641	Down-regulated in PsA
RP11-83M16.1	0.00903319	1.2172	Up-regulated in PsA
TMEM208	0.00903917	1.09598	Up-regulated in PsA
HMGB3P7	0.00905887	0.91116	Down-regulated in PsA
C10orf71	0.00907341	0.742987	Down-regulated in PsA
RP11-295P22.3	0.00908761	0.687144	Down-regulated in PsA
ZNF496	0.00911131	1.22176	Up-regulated in PsA
RN7SL828P	0.00913245	1.19489	Up-regulated in PsA
AC005752.10	0.00915632	1.26237	Up-regulated in PsA
MIR2681	0.00920157	1.21229	Up-regulated in PsA
RP11-16E18.1	0.00922775	0.88877	Down-regulated in PsA
IGFBP7-AS1	0.00922918	1.28132	Up-regulated in PsA
SMAD1-AS1	0.00924457	0.976898	Down-regulated in PsA
Metazoa_SRP	0.00925345	0.871311	Down-regulated in PsA
KIF15	0.00926003	0.87695	Down-regulated in PsA
RP11-16C1.1	0.00926756	1.47627	Up-regulated in PsA
ACIN1	0.00927566	0.77516	Down-regulated in PsA
AC006296.2	0.00929904	1.19304	Up-regulated in PsA
GLYATL2	0.00935543	0.781997	Down-regulated in PsA
HNRNPDLP3	0.00935725	1.32717	Up-regulated in PsA
MIR3646	0.00937937	1.33406	Up-regulated in PsA

AP001046.6	0.00939043	1.33851	Up-regulated in PsA
C22orf24	0.00939417	0.676629	Down-regulated in PsA
SLC25A6P6	0.00939544	0.78383	Down-regulated in PsA
FAM150B	0.00941495	0.739387	Down-regulated in PsA
TSR3	0.00943432	0.902891	Down-regulated in PsA
STK39	0.00943745	0.594441	Down-regulated in PsA
CTC-281M20.4	0.009438	1.17609	Up-regulated in PsA
RP11-43P8.2	0.00947182	0.881286	Down-regulated in PsA
AC092421.1	0.00949169	1.68645	Up-regulated in PsA
RP4-745E8.2	0.00951487	1.48782	Up-regulated in PsA
NHLH2	0.00953972	0.757468	Down-regulated in PsA
RP11-568G11.5	0.00954288	0.857045	Down-regulated in PsA
RP1-34L19.1	0.00954594	1.30378	Up-regulated in PsA
AE000658.31	0.00957309	0.82308	Down-regulated in PsA
HAUS7	0.00958654	0.824989	Down-regulated in PsA
PRKAG3	0.00960968	0.890706	Down-regulated in PsA
RP11-114G22.1	0.00961663	1.22614	Up-regulated in PsA
CTD-2538A21.1	0.0096271	0.79452	Down-regulated in PsA
RP11-629F19.1	0.00962845	0.786488	Down-regulated in PsA
LINC01033	0.00965541	0.794782	Down-regulated in PsA
CTD-2090I13.1	0.00965686	1.6754	Up-regulated in PsA
IGSF11-AS1	0.00965974	0.642819	Down-regulated in PsA
RP11-311H10.7	0.00966359	1.20454	Up-regulated in PsA
NR2F2	0.00966819	1.22716	Up-regulated in PsA
NUDCD1	0.00968296	0.595954	Down-regulated in PsA

RNF216	0.00971042	0.923692	Down-regulated in PsA
SPANXN4	0.00973959	1.07312	Up-regulated in PsA
LOC101927811	0.00975689	1.5596	Up-regulated in PsA
TTC23L	0.00977304	1.25988	Up-regulated in PsA
CCDC17	0.0097979	1.15367	Up-regulated in PsA
LOC100144595	0.00980863	0.862234	Down-regulated in PsA
MTHFD2	0.00981298	0.861188	Down-regulated in PsA
PRR21	0.0098216	1.15407	Up-regulated in PsA
MIR3976HG	0.00982674	1.13995	Up-regulated in PsA
FLI1	0.00984317	0.858482	Down-regulated in PsA
PKMP1	0.00984694	0.871688	Down-regulated in PsA
RP11-11C20.3	0.00989917	0.895632	Down-regulated in PsA
RP11-98G13.1	0.00995922	0.790235	Down-regulated in PsA
Y_RNA	0.00997178	0.83594	Down-regulated in PsA
RP11-995C19.2	0.00997399	0.876103	Down-regulated in PsA
AMIGO2	0.0100595	0.788473	Down-regulated in PsA
VCX2	0.0100732	1.11516	Up-regulated in PsA
RP11-702C7.1	0.0100806	1.30284	Up-regulated in PsA
RP11-411A19.5	0.0100923	1.67013	Up-regulated in PsA
SNX27	0.0100982	0.845899	Down-regulated in PsA
RP11-312J18.3	0.0101408	0.915577	Down-regulated in PsA
AC109309.4	0.0101569	0.874609	Down-regulated in PsA
HLA-DQB2	0.0101603	1.18119	Up-regulated in PsA
SAP30L	0.010162	0.738738	Down-regulated in PsA
ACER2	0.0101696	1.28452	Up-regulated in PsA

RP11-638L3.4	0.0102109	1.11539	Up-regulated in PsA
MIR1269B	0.0102144	1.1584	Up-regulated in PsA
ASZ1	0.010224	0.832705	Down-regulated in PsA
MIR7-3	0.0102619	0.760302	Down-regulated in PsA
SRXN1	0.0103012	1.06558	Up-regulated in PsA
RP11-420H19.3	0.01035	0.861672	Down-regulated in PsA
WBSCR16	0.0103999	0.875353	Down-regulated in PsA
DOCK7	0.0104919	0.495701	Down-regulated in PsA
RP11-193M21.1	0.0105124	1.05015	Up-regulated in PsA
MIR8063	0.0105201	1.40823	Up-regulated in PsA
NXT1	0.0105532	1.31902	Up-regulated in PsA
LINC01082	0.0105535	0.80162	Down-regulated in PsA
TRIP12	0.0105685	0.77102	Down-regulated in PsA
RUFY1	0.0105781	0.72674	Down-regulated in PsA
RP11-554D14.7	0.0105925	1.51588	Up-regulated in PsA
UTP14A	0.0106136	0.743574	Down-regulated in PsA
LINC01476	0.0106226	0.796715	Down-regulated in PsA
FKRP	0.0106493	1.23999	Up-regulated in PsA
CDK5RAP3	0.0106616	0.824084	Down-regulated in PsA
RNY1P8	0.0106707	1.39006	Up-regulated in PsA
RP11-927P21.2	0.0106773	1.64683	Up-regulated in PsA
RP11-267M23.7	0.0107055	1.2848	Up-regulated in PsA
CCNJL	0.0107332	1.10393	Up-regulated in PsA
SLC52A2	0.0107502	0.885048	Down-regulated in PsA
CTSD	0.0107855	0.919197	Down-regulated in PsA

PDLIM3	0.0108171	0.888745	Down-regulated in PsA
ITIH2	0.0108217	1.47037	Up-regulated in PsA
ELP6	0.0108403	0.610795	Down-regulated in PsA
RP11-529P9.1	0.0108737	1.43873	Up-regulated in PsA
RP11-257O5.2	0.0108916	0.725269	Down-regulated in PsA
CGN	0.0109701	0.795923	Down-regulated in PsA
C1orf54	0.0109784	0.68481	Down-regulated in PsA
CCDC149	0.0109908	0.813711	Down-regulated in PsA
AREL1	0.0109961	0.919822	Down-regulated in PsA
RP11-711M9.2	0.0110471	1.09394	Up-regulated in PsA
RIMKLBP2	0.0110744	0.938779	Down-regulated in PsA
PRR25	0.0111175	0.924818	Down-regulated in PsA
RN7SL262P	0.0111226	1.06217	Up-regulated in PsA
NME1P1	0.0111288	1.18641	Up-regulated in PsA
RPP40	0.0111359	0.700754	Down-regulated in PsA
RP11-239C9.1	0.0111379	0.844854	Down-regulated in PsA
KCNMA1-AS1	0.0111821	1.26179	Up-regulated in PsA
RP11-424N24.2	0.0112187	1.2649	Up-regulated in PsA
RP11-34P13.7	0.0112405	0.778477	Down-regulated in PsA
NMRK1	0.0112448	0.569071	Down-regulated in PsA
CHMP5	0.0112546	0.765145	Down-regulated in PsA
LOC101927394	0.0112672	1.39075	Up-regulated in PsA
SYT2	0.0112764	0.8176	Down-regulated in PsA
PIP4K2A	0.0112977	0.778446	Down-regulated in PsA
ATP6V1H	0.0113081	0.730852	Down-regulated in PsA

RP4-673D20.6	0.0113349	0.536008	Down-regulated in PsA
C16orf86	0.0113438	1.08971	Up-regulated in PsA
SNX10	0.011368	0.828148	Down-regulated in PsA
LAPTM4BP2	0.0113686	0.793956	Down-regulated in PsA
ERP44	0.0113704	0.865122	Down-regulated in PsA
RP11-794P6.6	0.0113744	0.784718	Down-regulated in PsA
RP11-302K17.4	0.0113753	0.95065	Down-regulated in PsA
RP11-417J1.5	0.011394	1.40301	Up-regulated in PsA
RP11-151H2.1	0.0114207	1.31578	Up-regulated in PsA
RP11-323J4.1	0.011445	1.11927	Up-regulated in PsA
IFNWP2	0.0114519	0.687017	Down-regulated in PsA
LINC01122	0.0114828	1.22302	Up-regulated in PsA
AC004945.1	0.0114928	0.827606	Down-regulated in PsA
WDFY3-AS1	0.011532	0.84985	Down-regulated in PsA
SORD2P	0.0115894	0.760022	Down-regulated in PsA
RP11-294J22.5	0.011626	0.936774	Down-regulated in PsA
RP11-298D21.3	0.0116517	1.40166	Up-regulated in PsA
CCNYL2	0.0116539	1.33503	Up-regulated in PsA
CHORDC1	0.0116703	0.642811	Down-regulated in PsA
SIAH1	0.0117034	1.11325	Up-regulated in PsA
CASC17	0.0117276	1.2345	Up-regulated in PsA
AC068580.5	0.0117303	1.1352	Up-regulated in PsA
OR10T2	0.0117352	0.815462	Down-regulated in PsA
RP11-533K11.1	0.0117732	0.882726	Down-regulated in PsA
RP4-583P15.10	0.0117764	0.858618	Down-regulated in PsA

RP11-358B23.1	0.0118105	1.21078	Up-regulated in PsA
UBA6-AS1	0.0118136	0.842007	Down-regulated in PsA
LOC101928896	0.0118297	1.11949	Up-regulated in PsA
MTND6P12	0.011833	1.32419	Up-regulated in PsA
RP11-539I5.1	0.0118972	1.14016	Up-regulated in PsA
GHR	0.0119682	0.85937	Down-regulated in PsA
CHRDL1	0.0119719	0.577259	Down-regulated in PsA
SURF4	0.0119725	0.832016	Down-regulated in PsA
AIPL1	0.0119901	0.760607	Down-regulated in PsA
TGDS	0.012012	0.580223	Down-regulated in PsA
CANX	0.012033	0.927668	Down-regulated in PsA
RN7SL221P	0.0120558	1.22277	Up-regulated in PsA
VN1R36P	0.0120986	0.893242	Down-regulated in PsA
ELL2P2	0.0121377	1.23209	Up-regulated in PsA
RP4-797C5.2	0.0121406	1.48382	Up-regulated in PsA
RP11-659P15.1	0.0121608	1.25358	Up-regulated in PsA
DNAJC19P6	0.0121766	0.716618	Down-regulated in PsA
RP11-322L17.1	0.0121815	1.07536	Up-regulated in PsA
MEIKIN	0.0121817	0.579636	Down-regulated in PsA
MIR499A	0.012218	1.11459	Up-regulated in PsA
PRRG1	0.0122315	0.676605	Down-regulated in PsA
SERBP1P4	0.0122457	1.32829	Up-regulated in PsA
SNORD8	0.0122791	1.13509	Up-regulated in PsA
RP11-347H15.2	0.0123047	0.676524	Down-regulated in PsA
ZAR1L	0.0123119	1.08784	Up-regulated in PsA

MYL6BP1	0.0123158	0.822375	Down-regulated in PsA
GDF7	0.012339	0.788093	Down-regulated in PsA
RP11-351M8.2	0.0123396	1.26322	Up-regulated in PsA
GBAT2	0.0123479	1.34782	Up-regulated in PsA
PCNA-AS1	0.0123647	0.769849	Down-regulated in PsA
CLEC2D	0.0123736	0.873309	Down-regulated in PsA
RP11-416A14.1	0.0123784	0.692192	Down-regulated in PsA
AC005822.1	0.0123923	0.826062	Down-regulated in PsA
RP11-361M10.4	0.0123975	0.846087	Down-regulated in PsA
ASCL5	0.0123993	1.27308	Up-regulated in PsA
RP11-95L3.2	0.0124005	1.24258	Up-regulated in PsA
Y_RNA	0.0124189	0.937966	Down-regulated in PsA
PCNAP3	0.0124359	0.688647	Down-regulated in PsA
CTC-525D6.2	0.0124367	1.34938	Up-regulated in PsA
PRDM15	0.0124423	0.841196	Down-regulated in PsA
DDAH1	0.0124825	0.753633	Down-regulated in PsA
RP11-321E2.7	0.0124918	1.33435	Up-regulated in PsA
DNAI1	0.0124926	1.16604	Up-regulated in PsA
LSAMP-AS1	0.0124938	1.0681	Up-regulated in PsA
FHAD1	0.0124941	0.771479	Down-regulated in PsA
PPFIA1	0.0125023	0.698509	Down-regulated in PsA
RN7SKP12	0.0125559	0.769873	Down-regulated in PsA
CTC-422A18.2	0.0125915	1.2107	Up-regulated in PsA
SNTG1	0.0125916	0.683986	Down-regulated in PsA
AC208162.1	0.0126081	1.16145	Up-regulated in PsA

MORF4L1P7	0.0126197	0.793712	Down-regulated in PsA
BST1	0.0126214	0.783351	Down-regulated in PsA
SUSD1	0.0126243	0.719639	Down-regulated in PsA
RP11-400N13.3	0.0126294	0.874071	Down-regulated in PsA
CTD-2128A3.2	0.0126394	1.83614	Up-regulated in PsA
RP11-1084A12.2	0.0126513	0.80328	Down-regulated in PsA
IFIH1	0.0126615	0.717218	Down-regulated in PsA
MAMDC4	0.0126621	0.806503	Down-regulated in PsA
TAF6L	0.0127	1.08999	Up-regulated in PsA
ENOX1-AS2	0.0127124	1.30147	Up-regulated in PsA
GS1-124K5.9	0.01273	1.26836	Up-regulated in PsA
DMTN	0.0127477	1.05981	Up-regulated in PsA
RN7SL52P	0.0127489	1.48746	Up-regulated in PsA
RPL21P38	0.0127501	1.16386	Up-regulated in PsA
KNTC1	0.0127569	0.75763	Down-regulated in PsA
RP11-452C13.1	0.0127638	0.76602	Down-regulated in PsA
LOC100996583	0.012797	1.61248	Up-regulated in PsA
CTD-2184C24.2	0.0128007	0.605893	Down-regulated in PsA
PLCG2	0.0128606	0.803241	Down-regulated in PsA
OR2T8	0.012905	1.37211	Up-regulated in PsA
RGS5	0.0129053	0.622202	Down-regulated in PsA
AC007386.4	0.0129282	0.463875	Down-regulated in PsA
ADAMTS13	0.0129284	0.807631	Down-regulated in PsA
TMEM204	0.0129308	1.19281	Up-regulated in PsA
CTBP2P6	0.0129352	0.949251	Down-regulated in PsA

LOC284395	0.0129574	1.42025	Up-regulated in PsA
RABEPK	0.0129635	0.920463	Down-regulated in PsA
RALGAPB	0.0130014	0.765285	Down-regulated in PsA
RP11-797E24.1	0.0130467	0.808183	Down-regulated in PsA
DR1	0.0130508	0.788779	Down-regulated in PsA
TXNDC12	0.0130822	0.821215	Down-regulated in PsA
RP11-148O21.6	0.0131007	1.44312	Up-regulated in PsA
SSTR1	0.0131062	1.22458	Up-regulated in PsA
TCP1P1	0.0131079	0.87618	Down-regulated in PsA
LINC01349	0.0131169	0.809369	Down-regulated in PsA
RP11-478B11.2	0.01312	0.691119	Down-regulated in PsA
RP3-333H23.9	0.0131413	0.902614	Down-regulated in PsA
FAM84B	0.0131416	0.785875	Down-regulated in PsA
LINC01479	0.0131421	1.46249	Up-regulated in PsA
CHCHD4P5	0.0131917	1.29088	Up-regulated in PsA
LOC101929058	0.013221	1.1447	Up-regulated in PsA
HIST2H2AB	0.013225	1.20024	Up-regulated in PsA
AC012462.3	0.0132342	1.32378	Up-regulated in PsA
SGOL1P1	0.0132687	1.31422	Up-regulated in PsA
SLFN5	0.0132981	0.611826	Down-regulated in PsA
RP11-279F6.2	0.0132991	1.39376	Up-regulated in PsA
INMT	0.0133092	0.906965	Down-regulated in PsA
LOC399715	0.0133405	1.135	Up-regulated in PsA
RP11-329B9.4	0.0133424	0.913492	Down-regulated in PsA
RP11-26L20.3	0.0133723	1.57032	Up-regulated in PsA

GS1-309P15.3	0.0133919	0.801215	Down-regulated in PsA
RN7SKP98	0.0134027	1.26204	Up-regulated in PsA
RP11-211N8.6	0.0134209	1.29277	Up-regulated in PsA
AC004066.3	0.0134472	1.24735	Up-regulated in PsA
SMUG1P1	0.0134596	0.812584	Down-regulated in PsA
CYCSP20	0.013572	1.12658	Up-regulated in PsA
RP11-1299A16.3	0.0135811	1.43898	Up-regulated in PsA
DNMBP-AS1	0.0136088	1.80334	Up-regulated in PsA
LOC100507642	0.0136834	0.595464	Down-regulated in PsA
SAG	0.0136855	0.750934	Down-regulated in PsA
HECTD2	0.0136997	0.712233	Down-regulated in PsA
RP11-108K3.4	0.0137251	1.39604	Up-regulated in PsA
NOTCH4	0.0137554	0.734452	Down-regulated in PsA
PLK1	0.0137783	1.26801	Up-regulated in PsA
AC074338.5	0.0138043	1.20361	Up-regulated in PsA
RN7SKP212	0.0138087	1.762	Up-regulated in PsA
CARNS1	0.0138136	1.2853	Up-regulated in PsA
Y_RNA	0.0138344	1.27806	Up-regulated in PsA
TAF12	0.0138451	0.677301	Down-regulated in PsA
VPS39	0.0138483	0.91078	Down-regulated in PsA
ARL11	0.01385	0.616554	Down-regulated in PsA
PALB2	0.0138556	0.814836	Down-regulated in PsA
GOT2	0.0138856	0.909648	Down-regulated in PsA
LINC01481	0.0139039	1.21551	Up-regulated in PsA
RP1-206D15.5	0.0139278	1.18233	Up-regulated in PsA

AL109767.1	0.013937	1.46454	Up-regulated in PsA
Y_RNA	0.0140061	0.850631	Down-regulated in PsA
FAM210A	0.0140126	0.855966	Down-regulated in PsA
AKAP3	0.0140201	1.35459	Up-regulated in PsA
GSTA7P	0.0140252	1.30586	Up-regulated in PsA
PQLC2L	0.0140567	0.552147	Down-regulated in PsA
GYG1	0.0140657	0.868128	Down-regulated in PsA
AC064834.1	0.0140684	0.862114	Down-regulated in PsA
TBC1D3B	0.0140692	0.846446	Down-regulated in PsA
FENDRR	0.0140734	0.886847	Down-regulated in PsA
AC007272.3	0.0140739	0.814527	Down-regulated in PsA
RP11-81A1.4	0.0140793	0.71674	Down-regulated in PsA
IFITM8P	0.0140798	1.41827	Up-regulated in PsA
SLIRP	0.0141255	0.920526	Down-regulated in PsA
SPEN	0.0141432	0.889605	Down-regulated in PsA
CETN3	0.0141563	0.703829	Down-regulated in PsA
USP17L19	0.0141666	0.672169	Down-regulated in PsA
ZNF793	0.0141764	0.869854	Down-regulated in PsA
RPL23AP39	0.0142154	1.11581	Up-regulated in PsA
LETM1P2	0.0142526	1.19443	Up-regulated in PsA
FAM3D	0.0142652	0.869692	Down-regulated in PsA
RP11-177G23.2	0.0143076	0.928587	Down-regulated in PsA
NPC2	0.0143444	0.900601	Down-regulated in PsA
OR8S1	0.0143587	0.614034	Down-regulated in PsA
PRKX	0.0143914	0.726588	Down-regulated in PsA

MIR34A	0.0144172	1.19853	Up-regulated in PsA
RP11-241N4.2	0.0144327	1.13512	Up-regulated in PsA
DBF4B	0.0144455	0.745283	Down-regulated in PsA
RP11-419M24.1	0.0144679	1.40696	Up-regulated in PsA
RN7SKP242	0.0145111	1.28875	Up-regulated in PsA
EEF1DP4	0.0145139	1.31112	Up-regulated in PsA
RP11-248E9.6	0.014525	0.857665	Down-regulated in PsA
ZNF321P	0.0145504	1.02478	Up-regulated in PsA
SRPX	0.0146319	2.07591	Up-regulated in PsA
NOL10	0.0146454	0.804888	Down-regulated in PsA
DDX11-AS1	0.0146473	1.40578	Up-regulated in PsA
PCNPP5	0.0146635	1.39557	Up-regulated in PsA
RP11-107E5.3	0.0146717	0.704683	Down-regulated in PsA
RP11-388M20.2	0.0146854	1.17126	Up-regulated in PsA
RP11-169K16.4	0.0146942	0.930529	Down-regulated in PsA
ST6GALNAC4	0.0146973	1.26136	Up-regulated in PsA
RP11-517O13.3	0.0147115	0.81887	Down-regulated in PsA
RP11-689C9.1	0.0147191	0.877453	Down-regulated in PsA
WDR89	0.0147225	0.576633	Down-regulated in PsA
RP11-747H12.4	0.0147386	1.13448	Up-regulated in PsA
RP11-439A17.10	0.014747	0.631861	Down-regulated in PsA
RP11-368I23.3	0.0147575	0.849415	Down-regulated in PsA
MIR6735	0.014766	0.812285	Down-regulated in PsA
WIPF1	0.0147742	1.0489	Up-regulated in PsA
CRAT	0.0147775	1.07158	Up-regulated in PsA

MIR3162	0.014788	1.28211	Up-regulated in PsA
PRTN3	0.0148911	1.06257	Up-regulated in PsA
AC114755.3	0.0148973	0.925168	Down-regulated in PsA
UXT	0.0149073	1.1758	Up-regulated in PsA
NPTN	0.0149548	0.811273	Down-regulated in PsA
FRRS1	0.0149608	0.493081	Down-regulated in PsA
RP11-205K6.1	0.0149717	1.79124	Up-regulated in PsA
RQCD1	0.0149787	0.797466	Down-regulated in PsA
RN7SL359P	0.0150116	1.21893	Up-regulated in PsA
PRRC2C	0.0150244	0.929762	Down-regulated in PsA
RP11-23E19.2	0.0150719	0.68832	Down-regulated in PsA
ANKRD13C	0.0151438	0.702322	Down-regulated in PsA
MIR2052HG	0.0151583	1.12114	Up-regulated in PsA
NDUFAF2	0.0151935	0.83039	Down-regulated in PsA
CKS2	0.0152003	0.601748	Down-regulated in PsA
LINC01237	0.0152204	1.32609	Up-regulated in PsA
SORT1	0.0152412	0.726037	Down-regulated in PsA
RP11-51L5.5	0.0152429	0.810048	Down-regulated in PsA
RP11-543P15.1	0.0152473	1.10662	Up-regulated in PsA
RPS4XP14	0.0152681	1.32847	Up-regulated in PsA
RP1-290I10.2	0.0152735	0.678444	Down-regulated in PsA
LOC101928887	0.015307	1.1672	Up-regulated in PsA
OR5B17	0.0153341	0.570812	Down-regulated in PsA
RP11-304C12.4	0.0153373	1.10864	Up-regulated in PsA
RP11-117L5.4	0.0153661	0.786289	Down-regulated in PsA

MORC1-AS1	0.015388	0.862121	Down-regulated in PsA
LOC101927472	0.0153909	1.17037	Up-regulated in PsA
NCOA5	0.0153951	0.7564	Down-regulated in PsA
DYRK1B	0.0154253	0.879671	Down-regulated in PsA
ASCL3	0.0154339	0.932192	Down-regulated in PsA
AZGP1P1	0.0154496	0.800049	Down-regulated in PsA
ATP2C1	0.0154506	0.792584	Down-regulated in PsA
ZNF402P	0.0154691	0.736615	Down-regulated in PsA
MIR3139	0.0154718	1.4666	Up-regulated in PsA
TMPRSS11E	0.015505	1.3221	Up-regulated in PsA
LINC01422	0.0155177	0.847663	Down-regulated in PsA
RN7SL304P	0.0155535	1.16452	Up-regulated in PsA
MIR6819	0.0156085	1.29344	Up-regulated in PsA
TRIM67	0.0156208	0.727064	Down-regulated in PsA
7SK	0.0156331	0.87231	Down-regulated in PsA
AC092675.4	0.0156414	1.30307	Up-regulated in PsA
CASP10	0.0156491	0.815481	Down-regulated in PsA
RN7SL152P	0.0156531	1.20342	Up-regulated in PsA
RP11-643G5.1	0.0156675	1.26995	Up-regulated in PsA
RP11-426D19.1	0.0156911	0.639017	Down-regulated in PsA
HNRNPA3P9	0.0157094	1.21763	Up-regulated in PsA
FAM122A	0.0157219	0.814201	Down-regulated in PsA
LINC00240	0.0157355	0.823104	Down-regulated in PsA
FTH1P22	0.0157419	1.24518	Up-regulated in PsA
CHRFAM7A	0.0157641	0.634929	Down-regulated in PsA

KRT18P45	0.0157897	0.874473	Down-regulated in PsA
CNOT6	0.0157985	0.815923	Down-regulated in PsA
HDLBP	0.0158077	0.948886	Down-regulated in PsA
RP11-365O16.1	0.0158108	0.799706	Down-regulated in PsA
LOC102724917	0.0158191	1.34654	Up-regulated in PsA
AC074011.2	0.0158352	1.16583	Up-regulated in PsA
QPCT	0.0158693	0.874962	Down-regulated in PsA
RP11-446P9.1	0.0158698	0.635271	Down-regulated in PsA
RTN4RL2	0.0158878	0.864707	Down-regulated in PsA
RN7SL702P	0.0158934	0.714191	Down-regulated in PsA
SYNPO2L	0.0159069	1.1975	Up-regulated in PsA
SYNPR	0.0159295	0.803779	Down-regulated in PsA
MNAT1	0.0159597	0.704699	Down-regulated in PsA
CFAP99	0.0160137	0.808303	Down-regulated in PsA
RP11-325D15.2	0.0160295	1.47712	Up-regulated in PsA
SPRY4-IT1_1	0.0160308	0.671902	Down-regulated in PsA
HMMR-AS1	0.0160555	1.26259	Up-regulated in PsA
UQCRFS1P2	0.0160896	1.2016	Up-regulated in PsA
ICE2P1	0.0160952	0.788607	Down-regulated in PsA
SYT11	0.0161541	0.851486	Down-regulated in PsA
NUP58	0.0161667	0.781885	Down-regulated in PsA
AC009237.11	0.0161854	1.33131	Up-regulated in PsA
PPP2R2D	0.0162044	0.793093	Down-regulated in PsA
LINC00106	0.0162304	0.820251	Down-regulated in PsA
RP11-163O19.11	0.0162664	1.29452	Up-regulated in PsA

OR10H2	0.0162735	1.23855	Up-regulated in PsA
AP000892.6	0.0162883	0.826161	Down-regulated in PsA
HIST1H4J	0.0162991	0.871403	Down-regulated in PsA
OR2K2	0.0163144	0.736422	Down-regulated in PsA
RIIAD1	0.0163187	0.758072	Down-regulated in PsA
FANCD2OS	0.0163447	0.721836	Down-regulated in PsA
RP11-285B24.1	0.0164063	0.854916	Down-regulated in PsA
RAB11FIP5	0.0164556	0.725301	Down-regulated in PsA
RP1-302D9.2	0.0164674	1.28524	Up-regulated in PsA
RP1-149M18.4	0.0164677	1.1249	Up-regulated in PsA
AC138472.4	0.0164829	1.06412	Up-regulated in PsA
CHMP1B2P	0.0165964	1.22752	Up-regulated in PsA
RP11-196H14.3	0.0166226	1.19604	Up-regulated in PsA
CTD-2057J6.1	0.0166563	1.27676	Up-regulated in PsA
IPO11	0.0167654	0.787619	Down-regulated in PsA
ANKRD33B-AS1	0.0167756	0.681028	Down-regulated in PsA
GTF2F2	0.0167846	0.841199	Down-regulated in PsA
GZMK	0.0167895	2.72679	Up-regulated in PsA
FAM133DP	0.0167944	0.929857	Down-regulated in PsA
RP11-805L22.3	0.0168039	1.28031	Up-regulated in PsA
RARRES2P8	0.0168192	1.29742	Up-regulated in PsA
KLHL2P1	0.0168442	0.895179	Down-regulated in PsA
RP11-71H17.1	0.0168457	1.6249	Up-regulated in PsA
SLC2A13	0.0168476	0.74903	Down-regulated in PsA
RP4-751H13.5	0.0168592	1.19051	Up-regulated in PsA

RP13-192B19.2	0.0168735	1.18166	Up-regulated in PsA
LOC389247	0.0168821	1.12359	Up-regulated in PsA
RAD23B	0.0168981	0.907729	Down-regulated in PsA
CTD-3006G17.2	0.0169246	1.10248	Up-regulated in PsA
WASF2	0.0169533	1.0723	Up-regulated in PsA
SYCN	0.016986	1.35747	Up-regulated in PsA
DEPDC7	0.0169989	0.643557	Down-regulated in PsA
CTB-26E19.1	0.017095	1.31458	Up-regulated in PsA
RP11-687D19.1	0.0171075	0.927511	Down-regulated in PsA
MFSD14C	0.0171172	0.703848	Down-regulated in PsA
CHMP7	0.0171405	0.743544	Down-regulated in PsA
ACAA1	0.0171557	0.927201	Down-regulated in PsA
USP39	0.0171663	0.901638	Down-regulated in PsA
CCDC114	0.0172412	1.18658	Up-regulated in PsA
DMP1	0.0172565	0.867076	Down-regulated in PsA
RP11-250H24.7	0.0172716	1.45391	Up-regulated in PsA
RP11-795A2.2	0.0172864	1.2763	Up-regulated in PsA
ATP6V1E1	0.0172877	0.727702	Down-regulated in PsA
RP11-465L10.10	0.0172893	1.39402	Up-regulated in PsA
RP11-20D14.6	0.0173159	0.695902	Down-regulated in PsA
COL18A1-AS2	0.0173424	1.16075	Up-regulated in PsA
Metazoa_SRP	0.0173503	0.888684	Down-regulated in PsA
RP11-48O20.5	0.0173504	1.58567	Up-regulated in PsA
ELAVL1	0.0173678	0.894152	Down-regulated in PsA
PRM3	0.0173752	0.858603	Down-regulated in PsA

WWC2-AS1	0.017384	1.30938	Up-regulated in PsA
MYBBP1A	0.0173848	0.868042	Down-regulated in PsA
PLEKHB2	0.0173971	0.861432	Down-regulated in PsA
AC114730.7	0.0174025	1.15484	Up-regulated in PsA
PF4	0.0174538	2.02182	Up-regulated in PsA
RP11-770E5.2	0.0174545	1.35742	Up-regulated in PsA
FAM160A2	0.0174657	0.915063	Down-regulated in PsA
NUP50-AS1	0.0174905	0.750337	Down-regulated in PsA
DIMT1	0.0175513	0.754307	Down-regulated in PsA
RP11-729M20.1	0.0175964	0.89866	Down-regulated in PsA
MAK16	0.0176028	0.855824	Down-regulated in PsA
RP11-10J21.2	0.0176541	1.19296	Up-regulated in PsA
RP11-983C2.3	0.0176565	0.727904	Down-regulated in PsA
TCEB1P35	0.0176803	0.784056	Down-regulated in PsA
RP11-136O12.2	0.0176949	1.33008	Up-regulated in PsA
RP11-54I5.1	0.0177273	0.551702	Down-regulated in PsA
SH2D4B	0.0177321	1.16083	Up-regulated in PsA
AC020743.4	0.0177476	1.31179	Up-regulated in PsA
PGM3	0.0177549	0.589768	Down-regulated in PsA
SLCO4A1	0.0177623	0.855806	Down-regulated in PsA
ATXN3	0.0177625	0.768441	Down-regulated in PsA
RP11-333J10.2	0.0177856	0.861757	Down-regulated in PsA
RP11-318K15.2	0.0177879	0.718097	Down-regulated in PsA
SNORA14B	0.017797	1.32004	Up-regulated in PsA
YWHAH	0.0178351	0.7419	Down-regulated in PsA

AC006960.7	0.0178386	1.30169	Up-regulated in PsA
RP11-337N6.2	0.0178611	1.28715	Up-regulated in PsA
SLA2	0.017866	1.37493	Up-regulated in PsA
SMIM12	0.0178747	0.847768	Down-regulated in PsA
SLC15A2	0.0179188	0.669679	Down-regulated in PsA
RNU6-835P	0.0179522	1.28381	Up-regulated in PsA
MRPL20	0.0180122	0.894509	Down-regulated in PsA
CREB3L1	0.0180551	0.792143	Down-regulated in PsA
PDCD11	0.0180595	0.682719	Down-regulated in PsA
ZNF624	0.018065	0.607614	Down-regulated in PsA
GAP43	0.0180888	1.45852	Up-regulated in PsA
RP11-75L1.1	0.0181117	1.4078	Up-regulated in PsA
NOTCH2	0.0181676	0.908545	Down-regulated in PsA
CTAGE16P	0.0181679	0.750665	Down-regulated in PsA
WBP1LP6	0.0181728	0.71719	Down-regulated in PsA
ZC3H8	0.0181738	0.63811	Down-regulated in PsA
HMGN5	0.018217	0.817583	Down-regulated in PsA
ATM	0.0182896	0.84475	Down-regulated in PsA
MINPP1	0.0183039	0.666342	Down-regulated in PsA
SMIM14	0.0183531	0.690021	Down-regulated in PsA
RN7SKP278	0.0183662	1.49364	Up-regulated in PsA
GSG1	0.0183758	0.677412	Down-regulated in PsA
RP11-545G3.1	0.0184159	0.736202	Down-regulated in PsA
RP1-178F10.1	0.0184301	0.841511	Down-regulated in PsA
MLANA	0.0184312	0.676498	Down-regulated in PsA

EIF4EBP2P3	0.0184475	0.836286	Down-regulated in PsA
Metazoa_SRP	0.0184481	0.68337	Down-regulated in PsA
LOC441178	0.0184534	1.24807	Up-regulated in PsA
DBET	0.0185185	1.25487	Up-regulated in PsA
AMOT	0.0185199	0.835162	Down-regulated in PsA
LOC102724814	0.0185697	0.880819	Down-regulated in PsA
RP11-384J4.2	0.018583	1.30326	Up-regulated in PsA
RP11-290D2.5	0.0186073	0.745082	Down-regulated in PsA
ZC3H7A	0.0186083	1.06013	Up-regulated in PsA
KREMEN2	0.0186355	0.836913	Down-regulated in PsA
SSXP10	0.0186995	0.679593	Down-regulated in PsA
SNORD77	0.0187483	0.886156	Down-regulated in PsA
DCAF4L2	0.0187506	0.79433	Down-regulated in PsA
XKR3	0.0187511	0.881367	Down-regulated in PsA
AKR7A3	0.0187652	0.801105	Down-regulated in PsA
RP1-148H17.1	0.0187911	1.13723	Up-regulated in PsA
RP11-315E17.1	0.0187998	1.50431	Up-regulated in PsA
RP11-433O3.1	0.0188008	1.28259	Up-regulated in PsA
ATP6V1G1	0.0188028	0.897796	Down-regulated in PsA
VTRNA2-2P	0.0188032	0.672774	Down-regulated in PsA
TNFSF14	0.0188067	0.811751	Down-regulated in PsA
RP11-170L3.4	0.0188095	0.729645	Down-regulated in PsA
DKC1	0.0188189	0.781778	Down-regulated in PsA
CTD-2363C16.1	0.0188297	1.33977	Up-regulated in PsA
CTD-2366F13.2	0.0188743	1.62013	Up-regulated in PsA

RP11-53L24.1	0.0188747	1.09996	Up-regulated in PsA
TMEM237	0.0189005	1.17557	Up-regulated in PsA
CTD-2552B11.3	0.0189066	1.22946	Up-regulated in PsA
PTCH2	0.018955	0.92298	Down-regulated in PsA
SBF1	0.0189577	1.13332	Up-regulated in PsA
KCNJ2-AS1	0.0189608	0.579943	Down-regulated in PsA
MIR3926-2	0.0189731	0.744441	Down-regulated in PsA
ZNF705D	0.0189742	1.24469	Up-regulated in PsA
ZDHHC21	0.0189793	0.624503	Down-regulated in PsA
EPDR1	0.0189811	0.771774	Down-regulated in PsA
RP11-197B12.1	0.0190294	1.18076	Up-regulated in PsA
SIGLEC11	0.0190432	0.72625	Down-regulated in PsA
LOC100129973	0.0190662	1.62503	Up-regulated in PsA
SKIV2L	0.0190712	0.93047	Down-regulated in PsA
CHCHD2P1	0.0190833	0.798123	Down-regulated in PsA
NDUFC1	0.0190853	0.936891	Down-regulated in PsA
ACOX1	0.0190902	0.769621	Down-regulated in PsA
DYM	0.0190986	0.858114	Down-regulated in PsA
SMG1P7	0.0191065	0.781551	Down-regulated in PsA
LOC100506188	0.0191079	0.705351	Down-regulated in PsA
RP11-419I17.1	0.0191202	0.792948	Down-regulated in PsA
RP11-12A20.11	0.0191411	1.31495	Up-regulated in PsA
EBNA1BP2	0.0191974	0.859401	Down-regulated in PsA
MAP3K7CL	0.0192233	1.86997	Up-regulated in PsA
RP5-894D12.4	0.0192257	1.20491	Up-regulated in PsA

AC092162.1	0.0192417	0.899586	Down-regulated in PsA
TCEB1P13	0.0192768	0.704895	Down-regulated in PsA
RPL21P8	0.0192906	1.08356	Up-regulated in PsA
ADI1	0.019309	0.804776	Down-regulated in PsA
MIR7641-1	0.0193107	1.2913	Up-regulated in PsA
GKN2	0.019325	0.777372	Down-regulated in PsA
HPRT1P1	0.0193417	1.35989	Up-regulated in PsA
PDZPH1P	0.0193436	1.20543	Up-regulated in PsA
PIP4K2B	0.0193506	0.802716	Down-regulated in PsA
PCP4L1	0.0193545	0.863968	Down-regulated in PsA
TTI2	0.0193597	0.793068	Down-regulated in PsA
LOC728613	0.0193933	0.825965	Down-regulated in PsA
OR52N1	0.0194112	0.912864	Down-regulated in PsA
RP11-22N19.2	0.0194231	0.804914	Down-regulated in PsA
RAD17P1	0.0194419	0.889053	Down-regulated in PsA
NAA30	0.01947	0.7455	Down-regulated in PsA
TMLHE-AS1	0.0194882	1.3337	Up-regulated in PsA
KRT20	0.0194931	0.726887	Down-regulated in PsA
EXTL2P1	0.0195467	0.822914	Down-regulated in PsA
RP11-151N17.1	0.019549	1.11702	Up-regulated in PsA
RP11-219F10.1	0.0195606	0.751819	Down-regulated in PsA
BMP7	0.0195704	0.678616	Down-regulated in PsA
TACR3	0.0196665	0.485627	Down-regulated in PsA
PROC	0.0196768	0.906399	Down-regulated in PsA
TLX1	0.0196828	0.685978	Down-regulated in PsA

SPG20	0.0197527	0.725681	Down-regulated in PsA
NAPB	0.0197566	0.812473	Down-regulated in PsA
RP11-276M12.1	0.0197593	1.24014	Up-regulated in PsA
RP11-108E14.1	0.0197838	1.12497	Up-regulated in PsA
COL4A2	0.0197929	1.28739	Up-regulated in PsA
RP11-453N3.7	0.0197997	0.767813	Down-regulated in PsA
AC007277.3	0.019826	0.908734	Down-regulated in PsA
ASCC2	0.0198376	0.932375	Down-regulated in PsA
RP11-760D2.4	0.0198622	0.877175	Down-regulated in PsA
KB-318B8.7	0.0198623	1.46981	Up-regulated in PsA
GAS6-AS1	0.0199147	0.835311	Down-regulated in PsA
HAO1	0.019941	0.718023	Down-regulated in PsA
Y_RNA	0.0199474	1.15719	Up-regulated in PsA
CTD-2325M2.1	0.0199479	2.16951	Up-regulated in PsA
GLIS2	0.0199487	1.16523	Up-regulated in PsA
SETP8	0.0199528	0.565301	Down-regulated in PsA
PAN2	0.0199664	0.800124	Down-regulated in PsA
MIR3180-5	0.0200104	1.0966	Up-regulated in PsA
RP11-463I20.4	0.0200139	1.66578	Up-regulated in PsA
PRSS55	0.0200156	1.26807	Up-regulated in PsA
SEC24C	0.0200214	0.795202	Down-regulated in PsA
CCDC58	0.0200285	0.569073	Down-regulated in PsA
RP11-373F14.1	0.0200513	1.13001	Up-regulated in PsA
CTB-60B18.12	0.020078	1.14075	Up-regulated in PsA
EIF4ENIF1	0.02008	0.796216	Down-regulated in PsA

MOB3C	0.0200824	0.767912	Down-regulated in PsA
RP11-113O24.3	0.0200882	1.13123	Up-regulated in PsA
LSM14B	0.0201379	0.783253	Down-regulated in PsA
SLC26A6	0.0201564	0.824539	Down-regulated in PsA
ALOX5	0.0201575	0.924192	Down-regulated in PsA
RP11-856M7.4	0.0201602	1.39918	Up-regulated in PsA
ROCK1P1	0.0201752	0.713584	Down-regulated in PsA
LINC00511	0.0202645	1.18185	Up-regulated in PsA
AP2S1	0.0202828	0.948179	Down-regulated in PsA
LEAP2	0.0202912	0.799531	Down-regulated in PsA
RP5-944M2.4	0.0203293	1.04645	Up-regulated in PsA
LOC389831	0.020363	1.15309	Up-regulated in PsA
SIGLEC15	0.0204251	0.985713	Down-regulated in PsA
RASEF	0.0204311	1.23573	Up-regulated in PsA
HHAT	0.0204671	0.858157	Down-regulated in PsA
LOC440434	0.0204699	0.885982	Down-regulated in PsA
LOC101928358	0.0204821	0.942956	Down-regulated in PsA
RP4-641G12.4	0.0204876	0.666389	Down-regulated in PsA
VIPR1-AS1	0.0204957	0.876643	Down-regulated in PsA
UPF3B	0.0205169	0.708155	Down-regulated in PsA
LURAP1L-AS1	0.0205222	1.3086	Up-regulated in PsA
MTND5P4	0.0205361	1.30402	Up-regulated in PsA
RP11-21I4.2	0.0205449	0.792026	Down-regulated in PsA
RP11-30K9.7	0.0205575	1.48706	Up-regulated in PsA
AP4B1-AS1	0.0205621	0.477906	Down-regulated in PsA

Y_RNA	0.0205653	1.29596	Up-regulated in PsA
RNF43	0.0205718	0.904343	Down-regulated in PsA
RNU6-441P	0.0205732	0.847589	Down-regulated in PsA
MTATP6P16	0.0205893	0.860777	Down-regulated in PsA
CTD-3126B10.2	0.0206093	1.16678	Up-regulated in PsA
RAB9AP4	0.0206215	1.21603	Up-regulated in PsA
CYLC2	0.0206293	0.955061	Down-regulated in PsA
KIAA0100	0.0206293	0.952486	Down-regulated in PsA
RP11-582J16.4	0.0206305	1.25815	Up-regulated in PsA
AKAP13	0.0206352	0.780432	Down-regulated in PsA
BMP3	0.0206691	1.40898	Up-regulated in PsA
RP11-629N8.3	0.0206726	1.20225	Up-regulated in PsA
EEF1GP6	0.020719	1.08959	Up-regulated in PsA
MIR4451	0.0207287	0.784973	Down-regulated in PsA
HPN-AS1	0.0207293	1.04318	Up-regulated in PsA
SNX5P1	0.0207325	0.678436	Down-regulated in PsA
RP11-461L13.4	0.0207363	0.778197	Down-regulated in PsA
NACAP2	0.0207828	1.02954	Up-regulated in PsA
CTC-498M16.4	0.0208078	0.885884	Down-regulated in PsA
OR5AP2	0.0208125	0.74335	Down-regulated in PsA
TTL	0.0208263	0.907511	Down-regulated in PsA
RP11-162A12.3	0.0208296	1.18928	Up-regulated in PsA
UBR5	0.0208382	0.797174	Down-regulated in PsA
LOC101928162	0.0208713	1.24529	Up-regulated in PsA
TPMT	0.0208755	0.75415	Down-regulated in PsA

AD001527.7	0.0209436	1.03683	Up-regulated in PsA
EEF1A1P15	0.0209478	0.912038	Down-regulated in PsA
Y_RNA	0.0209553	0.800793	Down-regulated in PsA
ANKRD26P4	0.0210136	0.715536	Down-regulated in PsA
CNTN6	0.0210466	0.763279	Down-regulated in PsA
RP11-40H20.4	0.0210497	1.11023	Up-regulated in PsA
MIR518E	0.0210655	0.684677	Down-regulated in PsA
TRIM44	0.0210921	0.814904	Down-regulated in PsA
EIF2B5-AS1	0.0210987	1.28073	Up-regulated in PsA
FAP	0.021109	0.856127	Down-regulated in PsA
C2orf27AP1	0.0211348	0.865811	Down-regulated in PsA
RNA5SP350	0.0211469	1.11076	Up-regulated in PsA
CACNA1C-IT1	0.0211557	1.31874	Up-regulated in PsA
ANAPC4	0.021163	0.881069	Down-regulated in PsA
LOC105372672	0.0211727	1.20185	Up-regulated in PsA
AC005324.6	0.0211819	0.747	Down-regulated in PsA
MAB21L2	0.0212165	1.2236	Up-regulated in PsA
RP11-968A15.2	0.0212216	1.09478	Up-regulated in PsA
RP11-29H23.6	0.0212369	1.2414	Up-regulated in PsA
RP11-536C10.11	0.0212377	0.760156	Down-regulated in PsA
RP11-475A13.1	0.0212706	1.43155	Up-regulated in PsA
CLIC3	0.0212863	1.37773	Up-regulated in PsA
CACNA1C-IT2	0.0212989	1.58241	Up-regulated in PsA
ITSN2	0.021324	0.798066	Down-regulated in PsA
POLR1B	0.0213387	0.758308	Down-regulated in PsA

DNAJC6	0.0213538	1.42327	Up-regulated in PsA
CTC-215O4.4	0.0213553	1.32052	Up-regulated in PsA
ADAD1P2	0.0213637	1.32237	Up-regulated in PsA
DDX11L16	0.021373	1.86964	Up-regulated in PsA
DHX33	0.021403	0.679742	Down-regulated in PsA
MHRT	0.0214096	1.17842	Up-regulated in PsA
AC010468.1	0.0214117	1.19915	Up-regulated in PsA
OSBP	0.0214374	0.90746	Down-regulated in PsA
GAPVD1	0.0214599	0.80606	Down-regulated in PsA
GTF2IRD1	0.0214681	1.03935	Up-regulated in PsA
RP3-431P23.2	0.0215201	1.39468	Up-regulated in PsA
SFTPB	0.0215235	0.845953	Down-regulated in PsA
IQCK	0.0215345	1.43964	Up-regulated in PsA
SCARNA22	0.0215726	0.910386	Down-regulated in PsA
AC005498.4	0.0216293	0.802889	Down-regulated in PsA
IDH3A	0.021636	0.894865	Down-regulated in PsA
NEUROD1	0.0216371	0.888773	Down-regulated in PsA
RP11-100N21.1	0.0216694	1.01953	Up-regulated in PsA
TUBB4BP6	0.0216786	1.42421	Up-regulated in PsA
TMF1	0.0216836	0.784299	Down-regulated in PsA
LOC101927020	0.0216984	1.45186	Up-regulated in PsA
RGS4	0.0217185	1.36256	Up-regulated in PsA
RN7SL470P	0.0217291	1.04904	Up-regulated in PsA
RP11-124N3.3	0.0217423	1.14055	Up-regulated in PsA
CTSL	0.0217646	0.601047	Down-regulated in PsA

CTC-436P18.5	0.0217879	0.841057	Down-regulated in PsA
MAPK14	0.0218147	0.687047	Down-regulated in PsA
ANAPC5	0.0218168	0.898068	Down-regulated in PsA
MIPOL1	0.0218279	0.570345	Down-regulated in PsA
B3GALNT1P1	0.0218381	1.46657	Up-regulated in PsA
DNAH11	0.0218995	0.695589	Down-regulated in PsA
RP11-757H14.2	0.021905	0.854912	Down-regulated in PsA
SREK1IP1	0.0219057	0.757382	Down-regulated in PsA
PRAMEF10	0.0219102	0.8109	Down-regulated in PsA
GPR157	0.0219195	1.18183	Up-regulated in PsA
HP1BP3	0.0219291	0.882253	Down-regulated in PsA
C21orf59	0.0219322	0.843396	Down-regulated in PsA
VPS11	0.0219471	0.902523	Down-regulated in PsA
C10orf88	0.0219492	0.436408	Down-regulated in PsA
STX1B	0.0219819	1.73503	Up-regulated in PsA
LINC01372	0.0219885	0.587521	Down-regulated in PsA
RP4-785G19.2	0.0220049	1.1633	Up-regulated in PsA
ZMYM4	0.0220163	0.872016	Down-regulated in PsA
TAT	0.0220465	1.42251	Up-regulated in PsA
MIR558	0.0220739	0.772378	Down-regulated in PsA
RP13-616I3.1	0.022082	1.4256	Up-regulated in PsA
RND1	0.0221122	0.830445	Down-regulated in PsA
SNRNP35	0.0221555	0.866157	Down-regulated in PsA
RP11-546K22.3	0.0221763	1.23604	Up-regulated in PsA
RNF26	0.0222037	1.17515	Up-regulated in PsA

ETFDH	0.0222237	0.633569	Down-regulated in PsA
WRAP53	0.0222495	0.778695	Down-regulated in PsA
AC009237.4	0.0222572	0.948452	Down-regulated in PsA
CFAP77	0.022273	0.81457	Down-regulated in PsA
RP11-586D5.3	0.0222832	1.27151	Up-regulated in PsA
LINC00642	0.0223251	1.63664	Up-regulated in PsA
CASP5	0.0223336	0.765088	Down-regulated in PsA
Y_RNA	0.0223439	1.13401	Up-regulated in PsA
MYHAS	0.0224065	0.777833	Down-regulated in PsA
Y_RNA	0.02245	0.866315	Down-regulated in PsA
PFN1P11	0.0224554	1.25679	Up-regulated in PsA
CCDC38	0.0224933	0.796525	Down-regulated in PsA
NBPF21P	0.0224945	1.13295	Up-regulated in PsA
NBPF2P	0.0224967	0.862198	Down-regulated in PsA
C6orf25	0.0225149	1.32112	Up-regulated in PsA
RP11-336A10.7	0.0225486	1.07781	Up-regulated in PsA
LOC100130075	0.0225609	1.39664	Up-regulated in PsA
RP11-798K3.3	0.0225807	0.917154	Down-regulated in PsA
LOC643802	0.0226118	0.862231	Down-regulated in PsA
TIMM23	0.0226182	0.870433	Down-regulated in PsA
PAX7	0.0226682	0.840745	Down-regulated in PsA
RP11-517C16.2	0.0226906	1.29949	Up-regulated in PsA
ZNF532	0.0226971	0.715587	Down-regulated in PsA
TUBA8	0.0227001	0.903879	Down-regulated in PsA
RP11-1141N12.1	0.0227139	0.822602	Down-regulated in PsA

MIR6075	0.0227825	1.24194	Up-regulated in PsA
NAV2-AS4	0.0227955	1.49169	Up-regulated in PsA
HNRNPCL4	0.0228079	0.674554	Down-regulated in PsA
AC002386.1	0.0228202	0.914189	Down-regulated in PsA
RP1-13D10.2	0.0228421	0.752957	Down-regulated in PsA
PP7080	0.0228478	1.24837	Up-regulated in PsA
OR8G2	0.0229171	1.22237	Up-regulated in PsA
RN7SL764P	0.0229338	1.18596	Up-regulated in PsA
RP11-1072C15.6	0.0229385	0.663367	Down-regulated in PsA
MIR4472-1	0.0229457	1.25103	Up-regulated in PsA
MRPS9	0.0229587	0.781036	Down-regulated in PsA
RP11-542K23.7	0.022962	1.51913	Up-regulated in PsA
RP1-95L4.3	0.0229637	0.895278	Down-regulated in PsA
COPB1	0.0229755	0.820122	Down-regulated in PsA
AC009133.17	0.0229906	1.26143	Up-regulated in PsA
RP11-474L11.3	0.0230052	1.21599	Up-regulated in PsA
MTRF1LP1	0.0230215	1.26197	Up-regulated in PsA
BGLT3	0.0230895	0.628864	Down-regulated in PsA
RP11-882I15.1	0.0231062	1.06384	Up-regulated in PsA
DLG1-AS1	0.023137	1.26301	Up-regulated in PsA
SAMD14	0.0231687	1.2063	Up-regulated in PsA
RN7SKP83	0.0232007	1.20647	Up-regulated in PsA
DIAPH2	0.023207	0.847188	Down-regulated in PsA
MIR1302-8	0.0232215	1.24601	Up-regulated in PsA
LOC101929721	0.0232339	1.26834	Up-regulated in PsA

PAK1IP1	0.0232462	0.751605	Down-regulated in PsA
PPID	0.0232888	0.678186	Down-regulated in PsA
ADAM32	0.023297	0.63202	Down-regulated in PsA
ALG1	0.0233057	0.790346	Down-regulated in PsA
KIAA0753	0.0233106	0.693035	Down-regulated in PsA
RP11-309H21.3	0.0233197	1.42125	Up-regulated in PsA
RP11-340I6.7	0.0233434	1.20962	Up-regulated in PsA
RP11-530C5.2	0.0233898	0.7725	Down-regulated in PsA
RP11-1H8.5	0.0234094	1.1609	Up-regulated in PsA
RN7SL330P	0.0234098	1.36345	Up-regulated in PsA
C1orf95	0.0234173	0.882603	Down-regulated in PsA
OFD1P3Y	0.0234303	1.30087	Up-regulated in PsA
MIR1538	0.0234324	1.26668	Up-regulated in PsA
SNORA13	0.0234378	1.40018	Up-regulated in PsA
RP11-1082L8.4	0.0234735	0.838503	Down-regulated in PsA
AC010145.4	0.0235106	0.860027	Down-regulated in PsA
LL22NC03-24A12.8	0.0235142	1.46078	Up-regulated in PsA
OR5M11	0.0235431	0.559693	Down-regulated in PsA
CACNA1A	0.023553	0.776837	Down-regulated in PsA
RP11-746P2.5	0.023566	1.35092	Up-regulated in PsA
DDX39B-AS1	0.0235782	1.25862	Up-regulated in PsA
RP13-39P12.2	0.02358	0.848749	Down-regulated in PsA
RP11-68E19.1	0.0235853	0.770848	Down-regulated in PsA
KRI1	0.0236193	0.647284	Down-regulated in PsA
NTN5	0.023638	0.85983	Down-regulated in PsA

UBE2Z	0.0236519	1.15634 Up-regulated in PsA
RP11-431M3.1	0.0236849	0.859079 Down-regulated in PsA
MYCNUT	0.0237128	1.22998 Up-regulated in PsA
RP11-12A16.3	0.0237619	1.17866 Up-regulated in PsA
MYL12BP1	0.0237968	0.864669 Down-regulated in PsA
TNPO1P3	0.0238005	0.52588 Down-regulated in PsA
RP11-23E19.1	0.0238149	1.26061 Up-regulated in PsA
RP11-431K24.2	0.0238156	1.18624 Up-regulated in PsA
GLYCAM1	0.0238321	1.24675 Up-regulated in PsA
MRPL3P1	0.0238374	0.808676 Down-regulated in PsA
POTEB2	0.0238763	1.27004 Up-regulated in PsA
SNORD113-7	0.0239015	1.23845 Up-regulated in PsA
GPLD1	0.0239165	0.819672 Down-regulated in PsA
ZNF347	0.0239206	0.61531 Down-regulated in PsA
LINC00951	0.0239246	0.716109 Down-regulated in PsA
RP5-1132H15.2	0.0239256	0.873986 Down-regulated in PsA
RP11-291O7.1	0.0239381	0.785648 Down-regulated in PsA
RP1-96H9.5	0.0239692	1.13928 Up-regulated in PsA
ABCD2	0.0239814	0.590476 Down-regulated in PsA
RNMTL1P1	0.0239921	1.13825 Up-regulated in PsA
LINC00605	0.0240451	0.695844 Down-regulated in PsA
FRMPD2B	0.0240547	0.828948 Down-regulated in PsA
RAD9A	0.0240983	0.781374 Down-regulated in PsA
ZNF33BP1	0.0241637	0.833233 Down-regulated in PsA
RP11-510J16.5	0.0241719	0.61222 Down-regulated in PsA

CFAP161	0.0241942	1.37444	Up-regulated in PsA
RP11-399E6.4	0.0242013	1.18795	Up-regulated in PsA
RP11-234A1.1	0.024205	1.40774	Up-regulated in PsA
LINC01548	0.0242617	1.43886	Up-regulated in PsA
MIR367	0.0242935	1.05669	Up-regulated in PsA
AC108004.3	0.0243003	1.40483	Up-regulated in PsA
C9orf85	0.024321	0.733874	Down-regulated in PsA
CCT4	0.0243251	0.793996	Down-regulated in PsA
C9orf40	0.0243493	0.751785	Down-regulated in PsA
RP4-584D14.5	0.0243587	1.25515	Up-regulated in PsA
RP11-362L22.1	0.0243795	1.22971	Up-regulated in PsA
MIR5692C1	0.0243847	0.883853	Down-regulated in PsA
LOC100505609	0.0244118	0.711954	Down-regulated in PsA
MIR548AY	0.0244257	0.647893	Down-regulated in PsA
EPM2A	0.0244288	0.776564	Down-regulated in PsA
UBQLN1P1	0.024431	0.708482	Down-regulated in PsA
MED8	0.024459	0.846488	Down-regulated in PsA
RP4-593M8.1	0.0245242	0.669435	Down-regulated in PsA
GNPNAT1	0.0245393	0.666008	Down-regulated in PsA
AP000146.2	0.024588	1.19623	Up-regulated in PsA
LINC00933	0.0246308	0.959799	Down-regulated in PsA
RP11-118G23.1	0.0246321	1.48061	Up-regulated in PsA
CHCHD3P2	0.0246387	0.925957	Down-regulated in PsA
RP11-439C8.2	0.0246765	1.22969	Up-regulated in PsA
RP11-573C10.1	0.0247028	0.714487	Down-regulated in PsA

RP5-1186P10.1	0.024721	0.81078	Down-regulated in PsA
RP11-818O24.3	0.0247592	1.20205	Up-regulated in PsA
ADGRB1	0.0248141	0.756111	Down-regulated in PsA
NFX1	0.0248154	0.918611	Down-regulated in PsA
RN7SL503P	0.0248259	1.15273	Up-regulated in PsA
AC073850.6	0.0248425	0.668475	Down-regulated in PsA
LPCAT2	0.0248742	0.845235	Down-regulated in PsA
RP5-1139B12.2	0.0249038	0.732346	Down-regulated in PsA
ATP6V1E1P1	0.0249053	0.710674	Down-regulated in PsA
FAM133A	0.0249471	0.890594	Down-regulated in PsA
MIRLET7D	0.024948	1.05153	Up-regulated in PsA
UBE2B	0.0249508	0.838889	Down-regulated in PsA
RN7SKP107	0.0249678	1.12777	Up-regulated in PsA
RP1-37N7.1	0.024983	1.37948	Up-regulated in PsA
RP11-548K12.11	0.0249994	1.14882	Up-regulated in PsA
COQ5	0.0250057	0.684918	Down-regulated in PsA
KNOP1	0.0250079	0.721083	Down-regulated in PsA
ZNF780A	0.0250212	0.7421	Down-regulated in PsA
LINC00570	0.025058	0.831769	Down-regulated in PsA
RP11-439I14.3	0.0250766	1.69047	Up-regulated in PsA
AIFM2	0.0251033	0.606723	Down-regulated in PsA
RP11-108K3.3	0.0251103	0.860336	Down-regulated in PsA
RP11-753D20.1	0.0251222	0.880294	Down-regulated in PsA
SETX	0.0251249	0.833471	Down-regulated in PsA
RNY4P29	0.0251254	1.36992	Up-regulated in PsA

RP11-438F14.10	0.0251391	0.736269	Down-regulated in PsA
RP11-736G13.1	0.0251519	1.54641	Up-regulated in PsA
OTOS	0.0251707	0.687846	Down-regulated in PsA
PYM1	0.0251732	0.801609	Down-regulated in PsA
NKIRAS1	0.0251781	0.739509	Down-regulated in PsA
COPS8P3	0.0251881	1.169	Up-regulated in PsA
Y_RNA	0.0252196	0.924282	Down-regulated in PsA
AP000936.5	0.0252732	1.12528	Up-regulated in PsA
RP11-1084E5.1	0.0252774	0.792784	Down-regulated in PsA
ZNFX1	0.0252954	0.881708	Down-regulated in PsA
PTRH2	0.0252962	0.781455	Down-regulated in PsA
STMN2	0.0253005	1.22278	Up-regulated in PsA
TCEB1P6	0.0253123	1.32887	Up-regulated in PsA
PAX1	0.0253181	0.852891	Down-regulated in PsA
RN7SL846P	0.025326	1.39802	Up-regulated in PsA
LVCAT1	0.0253338	0.832261	Down-regulated in PsA
RPS4XP8	0.0253505	1.16413	Up-regulated in PsA
MTCO1P24	0.0253692	1.20845	Up-regulated in PsA
Y_RNA	0.0254193	0.864304	Down-regulated in PsA
TADA1	0.0254465	0.830386	Down-regulated in PsA
ARPC1A	0.0254641	0.822041	Down-regulated in PsA
ABCB10	0.0254679	0.710345	Down-regulated in PsA
RP11-356K22.1	0.0254805	0.629811	Down-regulated in PsA
RP11-10J5.1	0.0254991	1.32447	Up-regulated in PsA
NT5DC4	0.0255009	1.17508	Up-regulated in PsA

RP1-29C18.10	0.0255027	0.927091	Down-regulated in PsA
RNA5SP91	0.0255069	1.60243	Up-regulated in PsA
MIR5586	0.0255328	0.760242	Down-regulated in PsA
PEX14	0.0255401	0.83238	Down-regulated in PsA
USP9YP10	0.0255599	0.885965	Down-regulated in PsA
PRYP2	0.0255753	1.25925	Up-regulated in PsA
CTA-221G9.7	0.0255833	0.763866	Down-regulated in PsA
CST4	0.0255972	0.670668	Down-regulated in PsA
RP11-208P4.1	0.025601	1.31574	Up-regulated in PsA
TPI1P2	0.025604	0.912621	Down-regulated in PsA
FLJ43879	0.0256073	1.42896	Up-regulated in PsA
RP11-214K3.24	0.0256654	0.721694	Down-regulated in PsA
LOC101928185	0.0256701	1.46035	Up-regulated in PsA
MIR3617	0.0256819	1.05208	Up-regulated in PsA
TCEB1P24	0.0257067	0.860143	Down-regulated in PsA
TCEB1P24	0.0257067	0.860143	Down-regulated in PsA
TAF7L	0.0257219	0.571551	Down-regulated in PsA
RP11-354E23.4	0.0257768	0.578613	Down-regulated in PsA
MLXIP	0.0257931	1.29247	Up-regulated in PsA
MSRA	0.0258152	0.583873	Down-regulated in PsA
RP11-284F21.8	0.0258265	1.3871	Up-regulated in PsA
TP53TG3D	0.0258519	0.874481	Down-regulated in PsA
KB-1183D5.16	0.0258745	0.800129	Down-regulated in PsA
MTATP6P23	0.0258782	1.5354	Up-regulated in PsA
AC002551.1	0.0258869	0.704922	Down-regulated in PsA

CYP2D6	0.0258875	1.16377	Up-regulated in PsA
GEMIN2P1	0.0258918	1.46831	Up-regulated in PsA
RP5-849H19.2	0.025912	0.580426	Down-regulated in PsA
COX18	0.0259156	0.780325	Down-regulated in PsA
FMO5	0.025948	0.777368	Down-regulated in PsA
SNRPCP16	0.0259484	0.928079	Down-regulated in PsA
LOC285889	0.0259719	1.36448	Up-regulated in PsA
ECT2L	0.0260312	0.922388	Down-regulated in PsA
TMEM206	0.0260321	0.576896	Down-regulated in PsA
OR4F16	0.0260584	0.891465	Down-regulated in PsA
OR4F29	0.0260584	0.891465	Down-regulated in PsA
GON4L	0.0260757	0.929237	Down-regulated in PsA
ZBTB47	0.026081	0.761047	Down-regulated in PsA
CRTC3	0.0261301	1.11511	Up-regulated in PsA
SSPO	0.0261464	0.949878	Down-regulated in PsA
AC007679.4	0.0262022	1.16097	Up-regulated in PsA
LRRC3C	0.0262287	0.671028	Down-regulated in PsA
FPGT-TNNI3K	0.0262443	0.532659	Down-regulated in PsA
MIR130B	0.0262519	1.13403	Up-regulated in PsA
RP11-664I21.5	0.0262533	0.784596	Down-regulated in PsA
RP1-224A6.9	0.0262943	0.694483	Down-regulated in PsA
RP3-466P17.1	0.0263525	1.1625	Up-regulated in PsA
STX3	0.0263538	0.70192	Down-regulated in PsA
LOC100506125	0.0263598	1.32922	Up-regulated in PsA
TIFA	0.0263636	0.467679	Down-regulated in PsA

GBX2	0.0263718	0.743228 Down-regulated in PsA
LPAR5	0.026375	1.20303 Up-regulated in PsA
F2R	0.0263776	1.25958 Up-regulated in PsA
OR11J2P	0.026387	1.32433 Up-regulated in PsA
RP11-452L6.7	0.0263891	1.05424 Up-regulated in PsA
MDFIC	0.0264065	0.808846 Down-regulated in PsA
RP11-174O3.1	0.0264297	1.20545 Up-regulated in PsA
CTD-2651B20.5	0.0264424	1.25469 Up-regulated in PsA
KRT81	0.0264549	1.15543 Up-regulated in PsA
EMC4	0.0264744	0.871647 Down-regulated in PsA
FGFR3P6	0.0264762	0.827893 Down-regulated in PsA
NANOG	0.0265263	0.751676 Down-regulated in PsA
LRRC19	0.0265431	0.617696 Down-regulated in PsA
RBBP4	0.026559	0.810373 Down-regulated in PsA
MIR548A1	0.0265812	0.93246 Down-regulated in PsA
RP11-3D4.2	0.0265879	1.28936 Up-regulated in PsA
AC011754.1	0.0265991	0.846031 Down-regulated in PsA
REV3L	0.0265995	0.822068 Down-regulated in PsA
KRTAP25-1	0.0266287	0.763736 Down-regulated in PsA
RP11-166B2.5	0.0266416	0.946366 Down-regulated in PsA
LRIT2	0.0266521	0.85477 Down-regulated in PsA
SNORA25	0.0266679	0.675344 Down-regulated in PsA
RP11-427P5.2	0.026678	1.69394 Up-regulated in PsA
SNORA71C	0.0266949	1.26683 Up-regulated in PsA
ETFA	0.026709	0.733876 Down-regulated in PsA

RP11-449D8.5	0.0267207	0.822571	Down-regulated in PsA
KCNQ5-AS1	0.0267282	0.813722	Down-regulated in PsA
PCED1CP	0.0267423	1.45987	Up-regulated in PsA
MRGPRD	0.0267452	0.912701	Down-regulated in PsA
HIGD1AP10	0.0267736	0.801529	Down-regulated in PsA
EXOSC2	0.0267826	0.702928	Down-regulated in PsA
Y_RNA	0.0268032	1.26208	Up-regulated in PsA
LOC100288069	0.0268213	0.953096	Down-regulated in PsA
RP11-149I23.3	0.0268229	0.875428	Down-regulated in PsA
Y_RNA	0.0268295	1.35691	Up-regulated in PsA
HMGN2P11	0.026836	1.34961	Up-regulated in PsA
TOLLIP	0.026869	0.932847	Down-regulated in PsA
AC084121.14	0.0269163	0.86359	Down-regulated in PsA
VCP	0.0269527	0.875878	Down-regulated in PsA
AC007271.3	0.026982	1.56888	Up-regulated in PsA
TUBBP9	0.0269904	0.945648	Down-regulated in PsA
AC159540.3	0.0269949	0.925802	Down-regulated in PsA
PRB2	0.027003	0.871562	Down-regulated in PsA
RP11-127L20.5	0.0270321	0.890788	Down-regulated in PsA
ENPP7P9	0.0270392	0.971643	Down-regulated in PsA
MIR6859-3	0.0270564	1.24652	Up-regulated in PsA
Y_RNA	0.0270576	1.03406	Up-regulated in PsA
RP1-20N2.7	0.0270852	0.85247	Down-regulated in PsA
ZNF502	0.0270893	0.595178	Down-regulated in PsA
GTF2IP20	0.0271268	1.088	Up-regulated in PsA

MIR5091	0.0272064	1.30949	Up-regulated in PsA
RP11-277L2.5	0.02722	0.818339	Down-regulated in PsA
SLC22A10	0.0272202	0.780758	Down-regulated in PsA
OR4F21	0.027222	0.66156	Down-regulated in PsA
PHF1	0.0272347	1.49184	Up-regulated in PsA
TMEM187	0.0272518	0.544177	Down-regulated in PsA
RP11-549B18.1	0.0272645	1.69588	Up-regulated in PsA
EYA3	0.0272809	0.626811	Down-regulated in PsA
MIR3142	0.0273077	0.691587	Down-regulated in PsA
A2ML1	0.0273818	1.42429	Up-regulated in PsA
TTC31	0.0273915	0.891345	Down-regulated in PsA
PTPN6	0.0274019	0.932324	Down-regulated in PsA
RP4-575N6.2	0.027409	0.8747	Down-regulated in PsA
SETDB2	0.0274278	0.611784	Down-regulated in PsA
C8orf4	0.0274338	0.829524	Down-regulated in PsA
TMEM59	0.0274342	0.861872	Down-regulated in PsA
RP11-258O13.1	0.0274434	0.859974	Down-regulated in PsA
RP11-362I1.1	0.0274736	1.08873	Up-regulated in PsA
PARP11	0.0274743	0.89469	Down-regulated in PsA
LOC101927709	0.0274812	0.855077	Down-regulated in PsA
Metazoa_SRP	0.0274845	1.21149	Up-regulated in PsA
GTF2A2	0.0274993	0.778801	Down-regulated in PsA
GLRXP	0.0275584	0.793597	Down-regulated in PsA
ZFYVE21	0.0275952	0.661383	Down-regulated in PsA
RP11-428G5.5	0.0276237	0.393978	Down-regulated in PsA

CTC-471J1.2	0.027641	1.42474	Up-regulated in PsA
AP001610.9	0.027645	1.28297	Up-regulated in PsA
TDGF1P4	0.0276451	1.21844	Up-regulated in PsA
CTD-3113P16.5	0.0276527	1.21828	Up-regulated in PsA
TRIM43CP	0.0276697	1.16386	Up-regulated in PsA
LOC100130673	0.0277232	0.787142	Down-regulated in PsA
ADM5	0.0277844	0.794077	Down-regulated in PsA
AF241725.6	0.0277845	1.59433	Up-regulated in PsA
PNPLA7	0.0277848	1.10652	Up-regulated in PsA
ITM2C	0.0278157	1.56698	Up-regulated in PsA
RN7SL783P	0.0278159	1.3157	Up-regulated in PsA
CLEC1B	0.0278305	1.28833	Up-regulated in PsA
RPS6KB1	0.027839	0.871189	Down-regulated in PsA
ARHGEF6	0.0278465	0.908134	Down-regulated in PsA
AL133249.1	0.0278528	0.834069	Down-regulated in PsA
Metazoa_SRP	0.0278556	1.1538	Up-regulated in PsA
LOC401242	0.027886	1.23745	Up-regulated in PsA
SPRYD4	0.0278927	0.868637	Down-regulated in PsA
RP11-296C13.1	0.0278938	1.23765	Up-regulated in PsA
LINC00443	0.0278995	1.30438	Up-regulated in PsA
RP11-314P15.2	0.027919	1.19065	Up-regulated in PsA
NPM3	0.0279217	1.24856	Up-regulated in PsA
GAPDHP41	0.0279231	1.3011	Up-regulated in PsA
Metazoa_SRP	0.0279233	1.4054	Up-regulated in PsA
RP11-687M24.7	0.0279395	1.21459	Up-regulated in PsA

XX-FYM637E10_5.1	0.0279553	1.29654 Up-regulated in PsA
RP11-191F9.1	0.0279568	1.15252 Up-regulated in PsA
ZNF565	0.0279615	0.643019 Down-regulated in PsA
RP4-800M22.4	0.0279987	1.41286 Up-regulated in PsA
RP11-813F20.4	0.0280118	1.27494 Up-regulated in PsA
AC010900.2	0.0280383	1.13821 Up-regulated in PsA
SLC16A14P1	0.0280571	1.43636 Up-regulated in PsA
GS1-164F24.1	0.0280918	1.13454 Up-regulated in PsA
ABL1	0.0280937	0.839297 Down-regulated in PsA
STAC3	0.028132	0.679899 Down-regulated in PsA
AC008982.2	0.0281377	0.882196 Down-regulated in PsA
RP11-486A14.1	0.0281598	1.47715 Up-regulated in PsA
ANP32C	0.0281603	0.887853 Down-regulated in PsA
LOC101928438	0.0281724	1.18278 Up-regulated in PsA
HOTAIR	0.0281778	1.12547 Up-regulated in PsA
CH17-248H7.3	0.0281923	1.31413 Up-regulated in PsA
RP11-722M1.1	0.0282	1.36973 Up-regulated in PsA
CTD-2332E11.2	0.0282277	1.18874 Up-regulated in PsA
RN7SKP58	0.0282572	1.28731 Up-regulated in PsA
SOHLH1	0.0282599	1.06728 Up-regulated in PsA
ETV5-AS1	0.0282713	0.758833 Down-regulated in PsA
PDSS2	0.0282836	0.598606 Down-regulated in PsA
MTCYBP12	0.0282994	0.880778 Down-regulated in PsA
SRGAP3-AS1	0.0283042	1.31726 Up-regulated in PsA
OR2AE1	0.0283188	1.36154 Up-regulated in PsA

RPL27AP8	0.0283289	1.10022	Up-regulated in PsA
GNS	0.0283289	0.870387	Down-regulated in PsA
RP11-200A1.1	0.0283406	1.31243	Up-regulated in PsA
AADACL2	0.0283423	1.0773	Up-regulated in PsA
ATP11A	0.0283456	1.16628	Up-regulated in PsA
AHCY	0.0283717	1.0857	Up-regulated in PsA
RP5-1168A5.1	0.028377	0.641485	Down-regulated in PsA
RN7SL65P	0.0283778	1.29731	Up-regulated in PsA
AC079834.1	0.0283831	0.963432	Down-regulated in PsA
MIR3166	0.0283928	0.809189	Down-regulated in PsA
NDUFB4P11	0.0283933	0.468631	Down-regulated in PsA
RP1-149A16.12	0.0284103	1.56359	Up-regulated in PsA
RP11-389O22.1	0.0284185	0.639135	Down-regulated in PsA
DDX20	0.0284318	0.885644	Down-regulated in PsA
EN2	0.0284382	0.82109	Down-regulated in PsA
AC026150.8	0.0284563	0.565192	Down-regulated in PsA
NDUFA4L2	0.0284652	1.57974	Up-regulated in PsA
MDC1-AS1	0.0285007	0.808223	Down-regulated in PsA
RP11-455I9.1	0.028502	0.747708	Down-regulated in PsA
RP11-562A8.4	0.0285537	1.17888	Up-regulated in PsA
PTPN2P1	0.0285775	0.798945	Down-regulated in PsA
THAP10	0.0285842	0.75107	Down-regulated in PsA
EIF3H	0.0286019	0.931505	Down-regulated in PsA
IGSF6	0.0286065	0.752011	Down-regulated in PsA
OR2B4P	0.0286317	0.663047	Down-regulated in PsA

KCTD1	0.0286354	0.908403 Down-regulated in PsA
RP11-578F21.9	0.0286435	1.31371 Up-regulated in PsA
CORO2B	0.0286519	0.658305 Down-regulated in PsA
RP11-158L12.5	0.0286602	1.60713 Up-regulated in PsA
TNPO3	0.0286655	0.826266 Down-regulated in PsA
BECN2	0.0286663	1.21516 Up-regulated in PsA
RPS27	0.0286668	1.08849 Up-regulated in PsA
RPL23AP96	0.0286914	1.55137 Up-regulated in PsA
ZNF197	0.0287205	1.2335 Up-regulated in PsA
RP11-230L22.4	0.0287238	0.633238 Down-regulated in PsA
ACAT2	0.0287676	0.766119 Down-regulated in PsA
RP11-397G5.2	0.0287857	0.828732 Down-regulated in PsA
NDUFA5P5	0.0287875	0.708542 Down-regulated in PsA
PHOSPHO2	0.0287922	0.433209 Down-regulated in PsA
LINC00618	0.0288098	1.48627 Up-regulated in PsA
HSPA8P9	0.0288106	0.773773 Down-regulated in PsA
FCRL5	0.0288295	1.33605 Up-regulated in PsA
TUBBP6	0.0288411	1.1998 Up-regulated in PsA
RP13-395E19.2	0.0288576	1.05183 Up-regulated in PsA
ARPC3P2	0.0289052	0.730136 Down-regulated in PsA
FAM153B	0.0289134	0.676033 Down-regulated in PsA
RNU6-373P	0.0289159	1.35824 Up-regulated in PsA
TCEB3	0.0289215	0.726596 Down-regulated in PsA
HOTAIR_5	0.0289253	0.656074 Down-regulated in PsA
AC011330.5	0.0289363	1.66414 Up-regulated in PsA

METTL25	0.0289397	0.697256	Down-regulated in PsA
ATP10D	0.0289468	0.653866	Down-regulated in PsA
RP11-187E13.3	0.0289506	1.26608	Up-regulated in PsA
FAM89A	0.0289898	1.25251	Up-regulated in PsA
CICP16	0.0290252	1.1022	Up-regulated in PsA
TRAPPC4	0.029032	0.672063	Down-regulated in PsA
RP11-553E24.3	0.0290352	0.879392	Down-regulated in PsA
RP11-317P15.6	0.0290506	0.849916	Down-regulated in PsA
GOLGA2P2Y	0.0290934	0.849064	Down-regulated in PsA
BBS12	0.0291041	0.79892	Down-regulated in PsA
CNIH4	0.0291111	0.817293	Down-regulated in PsA
MIR181C	0.0291111	0.873682	Down-regulated in PsA
RP11-715F3.1	0.0291228	1.21146	Up-regulated in PsA
RALGAPA1	0.0291285	0.842278	Down-regulated in PsA
LINC00106	0.0291342	0.835845	Down-regulated in PsA
RP5-859M6.1	0.0291506	1.27772	Up-regulated in PsA
SRSF4	0.0291527	0.804784	Down-regulated in PsA
LOC102467079	0.0291527	0.815168	Down-regulated in PsA
pRNA	0.0292411	1.18617	Up-regulated in PsA
pRNA	0.0292411	1.18617	Up-regulated in PsA
pRNA	0.0292411	1.18617	Up-regulated in PsA
pRNA	0.0292411	1.18617	Up-regulated in PsA
pRNA	0.0292411	1.18617	Up-regulated in PsA
pRNA	0.0292411	1.18617	Up-regulated in PsA
pRNA	0.0292411	1.18617	Up-regulated in PsA

pRNA	0.0292411	1.18617	Up-regulated in PsA
AC010105.1	0.0292447	1.2208	Up-regulated in PsA
RBBP6	0.0292596	1.04532	Up-regulated in PsA
FABP4	0.0292662	1.42293	Up-regulated in PsA
MYO1F	0.0292756	0.973677	Down-regulated in PsA
AC013444.1	0.0292863	1.87952	Up-regulated in PsA
USP9YP22	0.0292946	1.09275	Up-regulated in PsA
TF	0.0293032	1.58088	Up-regulated in PsA
RP11-502F1.2	0.0293033	0.8149	Down-regulated in PsA
RPS3AP33	0.0293229	1.11286	Up-regulated in PsA
STAT6	0.0293369	0.87496	Down-regulated in PsA
LOC101927229	0.0293652	1.20422	Up-regulated in PsA
RP5-857K21.4	0.0293876	0.709808	Down-regulated in PsA
USP53	0.0293926	0.549341	Down-regulated in PsA
ZNF563	0.0294181	0.73171	Down-regulated in PsA
AP001619.3	0.0294504	1.10521	Up-regulated in PsA
AATK-AS1	0.029461	1.31231	Up-regulated in PsA
TAAR8	0.0294726	1.21888	Up-regulated in PsA
RPS23P9	0.0294728	1.6951	Up-regulated in PsA
RP11-64C12.7	0.0295545	0.869227	Down-regulated in PsA
RN7SKP77	0.0295633	0.780073	Down-regulated in PsA
SNORA28	0.0295688	1.08937	Up-regulated in PsA
BICD1	0.0296067	0.690598	Down-regulated in PsA
MEGF9	0.0296217	0.812021	Down-regulated in PsA
RP3-417O22.3	0.0296269	0.676436	Down-regulated in PsA

CTD-3126B10.5	0.0296339	0.799924	Down-regulated in PsA
PPP2R3C	0.0296456	0.755015	Down-regulated in PsA
UBE2N	0.0296831	0.726415	Down-regulated in PsA
RP11-360A18.2	0.029686	0.87159	Down-regulated in PsA
LINC01613	0.029686	0.87159	Down-regulated in PsA
RP1-8B22.2	0.0296998	0.793048	Down-regulated in PsA
RP11-467J12.2	0.0297035	1.07691	Up-regulated in PsA
AP000343.1	0.0297238	1.15148	Up-regulated in PsA
KCTD9P3	0.0297371	0.781694	Down-regulated in PsA
REXO4	0.0297897	0.842787	Down-regulated in PsA
DCAF13	0.0298042	0.679423	Down-regulated in PsA
RP11-593F5.1	0.0298145	0.848856	Down-regulated in PsA
BCAR1P2	0.0298221	1.17623	Up-regulated in PsA
NPAT	0.0298435	0.665063	Down-regulated in PsA
RP11-447M4.1	0.0298449	1.29704	Up-regulated in PsA
PLAC8L1	0.0299087	0.777428	Down-regulated in PsA
ARAP2	0.0299221	1.85078	Up-regulated in PsA
SLTM	0.0299302	0.833988	Down-regulated in PsA
LOC101927464	0.0299323	1.15338	Up-regulated in PsA
RN7SL254P	0.029937	1.16254	Up-regulated in PsA
RP11-973D8.4	0.0299387	1.65543	Up-regulated in PsA
PTP4A1P1	0.0299667	0.759061	Down-regulated in PsA
OR52A1	0.0299865	0.681623	Down-regulated in PsA
RNF8	0.029993	0.564927	Down-regulated in PsA
CYP27C1	0.0299953	0.531588	Down-regulated in PsA

RN7SL443P	0.0300038	1.18801	Up-regulated in PsA
BNIP3P1	0.0300093	0.857765	Down-regulated in PsA
PRKCB	0.0300152	0.749898	Down-regulated in PsA
AC140725.8	0.0300319	0.585401	Down-regulated in PsA
RP11-3P17.5	0.0300617	1.09018	Up-regulated in PsA
RP11-111A22.1	0.0300681	1.14114	Up-regulated in PsA
LDHBP1	0.0300849	1.41683	Up-regulated in PsA
CTC-428G20.3	0.0300866	0.700221	Down-regulated in PsA
CKAP5	0.0301508	0.767726	Down-regulated in PsA
STIL	0.0301606	0.54504	Down-regulated in PsA
SETP15	0.0301891	0.839559	Down-regulated in PsA
RMDN1	0.0302192	0.851111	Down-regulated in PsA
RP11-692D12.1	0.030239	0.704216	Down-regulated in PsA
FAM69B	0.0302497	0.79661	Down-regulated in PsA
PLCXD3	0.0302517	1.29565	Up-regulated in PsA
EIF4HP1	0.0302625	0.848271	Down-regulated in PsA
SNORA40	0.0302734	0.817037	Down-regulated in PsA
GUCY1A3	0.0303313	0.656245	Down-regulated in PsA
AC024162.2	0.0303407	0.880282	Down-regulated in PsA
RP11-622J8.1	0.030362	1.18808	Up-regulated in PsA
HSPH1	0.0303726	0.636086	Down-regulated in PsA
AC005614.5	0.0303922	0.645566	Down-regulated in PsA
AC019097.7	0.030403	0.762053	Down-regulated in PsA
RP11-365D23.4	0.0304143	2.20167	Up-regulated in PsA
RN7SL188P	0.0304171	1.16726	Up-regulated in PsA

Y_RNA	0.0304223	0.897798	Down-regulated in PsA
RPL35AP4	0.0304581	1.25277	Up-regulated in PsA
DUS3L	0.0304645	1.12662	Up-regulated in PsA
RP13-895J2.10	0.0304843	1.19541	Up-regulated in PsA
RP11-475O23.2	0.0304891	1.17124	Up-regulated in PsA
TCEB1P17	0.0305193	1.25207	Up-regulated in PsA
MIR32	0.0305207	0.680902	Down-regulated in PsA
GPR137B	0.0305335	0.828291	Down-regulated in PsA
PMVK	0.0305765	0.72285	Down-regulated in PsA
RP11-608B3.1	0.0306025	0.876085	Down-regulated in PsA
MIR99A	0.0306217	1.24181	Up-regulated in PsA
BDP1	0.0306332	0.690277	Down-regulated in PsA
CTD-2008E3.1	0.0306346	0.946109	Down-regulated in PsA
GART	0.0306388	0.710546	Down-regulated in PsA
RN7SL448P	0.0306407	1.19156	Up-regulated in PsA
ZBTB46-AS1	0.0306434	1.03264	Up-regulated in PsA
RP11-526K21.2	0.0306512	1.40971	Up-regulated in PsA
DHODH	0.0306555	0.787522	Down-regulated in PsA
RPL12P9	0.0306807	0.81202	Down-regulated in PsA
RP5-1164C1.2	0.0306882	1.28634	Up-regulated in PsA
RP11-96D1.8	0.0306925	1.11105	Up-regulated in PsA
DOK4	0.0307333	0.632465	Down-regulated in PsA
AC019185.2	0.0307591	0.842503	Down-regulated in PsA
OR4N1P	0.0307853	1.249	Up-regulated in PsA
RP11-302K17.3	0.030844	1.18302	Up-regulated in PsA

COL18A1	0.0308463	1.12259	Up-regulated in PsA
USP34	0.0308637	0.785467	Down-regulated in PsA
RP11-321E2.9	0.030866	1.46135	Up-regulated in PsA
TRIM26	0.0308919	0.806126	Down-regulated in PsA
MIR532	0.0308979	1.34771	Up-regulated in PsA
RP11-893F2.14	0.030905	1.24709	Up-regulated in PsA
BRICD5	0.0309054	1.35044	Up-regulated in PsA
RN7SL68P	0.0309483	0.773253	Down-regulated in PsA
SMIM10L2B	0.0309575	1.16447	Up-regulated in PsA
TRIM50	0.0309974	0.852604	Down-regulated in PsA
PARP1P1	0.0310399	0.81188	Down-regulated in PsA
CDH18	0.0310434	1.44564	Up-regulated in PsA
USP45	0.0310706	0.55508	Down-regulated in PsA
MIR3147	0.0310801	1.26009	Up-regulated in PsA
RP11-395N3.1	0.0311183	1.19532	Up-regulated in PsA
RP11-178H8.2	0.031129	1.14057	Up-regulated in PsA
BTN2A1	0.0311635	0.700908	Down-regulated in PsA
ALAD	0.0312111	0.885269	Down-regulated in PsA
CLECL1	0.0312379	2.62436	Up-regulated in PsA
ZNF540	0.0312529	0.752473	Down-regulated in PsA
KCCAT333	0.0312589	1.93481	Up-regulated in PsA
RP11-108M9.5	0.0312693	1.26263	Up-regulated in PsA
LOC102723604	0.0312729	1.49245	Up-regulated in PsA
KRT39	0.0312989	0.690348	Down-regulated in PsA
LENG8	0.0313029	0.951066	Down-regulated in PsA

RPSAP5	0.0313218	0.864886	Down-regulated in PsA
MIR4478	0.0313347	1.26094	Up-regulated in PsA
CITED4	0.0313557	0.906853	Down-regulated in PsA
DBIL5P2	0.0313625	1.38083	Up-regulated in PsA
MTCO1P45	0.0313634	1.20027	Up-regulated in PsA
RP11-1018N14.5	0.0313751	0.828515	Down-regulated in PsA
RP11-493L12.3	0.0314052	1.3897	Up-regulated in PsA
RP11-454L9.2	0.0314054	1.20373	Up-regulated in PsA
BUD31P1	0.0314354	1.20722	Up-regulated in PsA
NSFL1C	0.0314397	0.860885	Down-regulated in PsA
RP11-486M3.2	0.0314673	1.263	Up-regulated in PsA
OR7D2	0.0314766	0.869379	Down-regulated in PsA
LILRA2	0.0314887	0.831533	Down-regulated in PsA
ZCCHC9	0.0315007	0.84113	Down-regulated in PsA
HERC2P3	0.0315029	0.525553	Down-regulated in PsA
RP13-157F18.2	0.0315131	0.824122	Down-regulated in PsA
KB-1517D11.2	0.0315148	0.634883	Down-regulated in PsA
RN7SL420P	0.0315271	1.17681	Up-regulated in PsA
RP11-124N14.3	0.0315305	1.16529	Up-regulated in PsA
CTC-241F20.4	0.0315746	1.4836	Up-regulated in PsA
EFCAB11	0.0315864	0.512265	Down-regulated in PsA
XXbac-B476C20.11	0.0316131	1.14651	Up-regulated in PsA
CLYBL-AS1	0.0316203	1.70447	Up-regulated in PsA
DHX16	0.0316207	0.805608	Down-regulated in PsA
PCYT1A	0.0316271	0.775337	Down-regulated in PsA

TTC30B	0.0316319	0.67543	Down-regulated in PsA
ADRA1B	0.0316557	0.67581	Down-regulated in PsA
OR5D3P	0.0316625	1.25272	Up-regulated in PsA
DRD1	0.0316958	0.803064	Down-regulated in PsA
Six3os1_1	0.03171	1.21205	Up-regulated in PsA
RPL7P40	0.0317448	0.69346	Down-regulated in PsA
ZNF404	0.0317512	0.858093	Down-regulated in PsA
KLC3	0.0317692	0.929041	Down-regulated in PsA
TBL1XR1	0.0317779	0.666592	Down-regulated in PsA
CTD-3184A7.4	0.0318083	1.28768	Up-regulated in PsA
KCNT1	0.0318147	0.776282	Down-regulated in PsA
ZNF32	0.0318155	0.87633	Down-regulated in PsA
RP11-492L8.1	0.0318518	1.33294	Up-regulated in PsA
RP11-368M16.3	0.0318638	0.881112	Down-regulated in PsA
WDR86-AS1	0.0318707	1.19775	Up-regulated in PsA
AC254560.1	0.0319208	0.958509	Down-regulated in PsA
AGAP1-IT1	0.0319249	1.27101	Up-regulated in PsA
HNRNPA1P63	0.0319398	0.681051	Down-regulated in PsA
RP1-303A1.1	0.0319559	1.40721	Up-regulated in PsA
MRPS33	0.0319614	0.918318	Down-regulated in PsA
RP11-5C23.2	0.0319849	0.818671	Down-regulated in PsA
CCDC28A	0.0320065	0.772925	Down-regulated in PsA
ASCL4	0.0320157	0.743518	Down-regulated in PsA
CH17-258A22.4	0.0320265	0.789003	Down-regulated in PsA
RP11-989E6.13	0.0320463	1.26582	Up-regulated in PsA

AC092569.2	0.0320762	1.44714	Up-regulated in PsA
PPARGC1B	0.0320831	0.813003	Down-regulated in PsA
MRPS31P5	0.032108	1.24451	Up-regulated in PsA
C1orf74	0.0321508	0.804788	Down-regulated in PsA
KIF6	0.0321572	1.23915	Up-regulated in PsA
MECP2	0.0321706	0.895887	Down-regulated in PsA
RP11-644F5.15	0.0321793	1.4198	Up-regulated in PsA
UMOD	0.032191	1.35733	Up-regulated in PsA
ADRB1	0.0321996	0.87253	Down-regulated in PsA
AVIL	0.0322069	0.834623	Down-regulated in PsA
HTR3D	0.0322244	1.25511	Up-regulated in PsA
NDUFAF1	0.0322305	0.689817	Down-regulated in PsA
SLC2A1	0.0322309	1.51801	Up-regulated in PsA
RN7SKP230	0.0322538	1.14442	Up-regulated in PsA
CTD-2561B21.10	0.032286	1.37345	Up-regulated in PsA
ADGRF4	0.0322956	0.750437	Down-regulated in PsA
CTD-3118D11.3	0.0322995	0.607172	Down-regulated in PsA
RP11-325O24.1	0.0323329	0.659196	Down-regulated in PsA
CCBL1	0.0323465	0.75914	Down-regulated in PsA
LOC389602	0.0323665	0.877768	Down-regulated in PsA
LA16c-17H1.3	0.0323703	0.789364	Down-regulated in PsA
MRPL38	0.0323897	1.15543	Up-regulated in PsA
MIR6782	0.032478	1.07685	Up-regulated in PsA
KRTAP5-7	0.0324992	1.11447	Up-regulated in PsA
DARS-AS1	0.0325213	0.81582	Down-regulated in PsA

RP11-355K23B.1	0.0325225	1.19434	Up-regulated in PsA
CUZD1	0.0325455	0.839599	Down-regulated in PsA
MIR371B	0.0325731	0.549336	Down-regulated in PsA
RP11-94C24.6	0.0325834	1.21592	Up-regulated in PsA
PPIL1	0.0325896	0.70441	Down-regulated in PsA
CHRNA7	0.0327134	1.11198	Up-regulated in PsA
RP11-608O8.2	0.0327687	1.12451	Up-regulated in PsA
KB-1299A7.2	0.0328241	1.42979	Up-regulated in PsA
RP11-556H2.3	0.0328302	1.33215	Up-regulated in PsA
NPHS2	0.0328324	0.910705	Down-regulated in PsA
FAM109B	0.0328351	0.874956	Down-regulated in PsA
BNIP3P32	0.03286	1.25018	Up-regulated in PsA
MOB1A	0.03286	0.871803	Down-regulated in PsA
CPNE8	0.032879	0.861782	Down-regulated in PsA
RBBP4P5	0.032884	0.784564	Down-regulated in PsA
COIL	0.0328872	0.763129	Down-regulated in PsA
CTC-428G20.2	0.0328904	1.31969	Up-regulated in PsA
SLC30A6	0.032897	0.768241	Down-regulated in PsA
NUCKS1P1	0.0329141	1.29863	Up-regulated in PsA
SPATA22	0.0329222	0.485654	Down-regulated in PsA
AC073115.6	0.0329258	1.53792	Up-regulated in PsA
RP4-665N4.4	0.0329305	1.19898	Up-regulated in PsA
GFER	0.0329331	1.34332	Up-regulated in PsA
NT5E	0.0329396	0.84031	Down-regulated in PsA
LEO1	0.0329842	0.624464	Down-regulated in PsA

RP11-407A16.8	0.0329997	0.806788	Down-regulated in PsA
RP11-795H16.3	0.0330265	0.722732	Down-regulated in PsA
LOC100652768	0.0330365	0.865339	Down-regulated in PsA
ASS1P1	0.0330623	1.13	Up-regulated in PsA
DTNA	0.0330685	0.555095	Down-regulated in PsA
EVA1C	0.0330768	1.09558	Up-regulated in PsA
CTD-2396E7.7	0.0330873	1.23178	Up-regulated in PsA
PDRG1	0.0330897	0.83748	Down-regulated in PsA
RP5-845O24.8	0.03309	1.37085	Up-regulated in PsA
Y_RNA	0.0331134	1.40406	Up-regulated in PsA
SNORD87	0.0331141	0.850836	Down-regulated in PsA
AC093074.1	0.0331156	0.860503	Down-regulated in PsA
DEFA4	0.0331501	0.863931	Down-regulated in PsA
LOC101928441	0.0331745	1.30645	Up-regulated in PsA
FRYL	0.0331854	0.891533	Down-regulated in PsA
SHANK3	0.0332205	0.838114	Down-regulated in PsA
CERS2	0.0332323	0.868363	Down-regulated in PsA
RP11-404P21.9	0.0332684	1.1352	Up-regulated in PsA
LRRC30	0.0332722	0.807957	Down-regulated in PsA
CTD-2313J17.1	0.0332758	0.734658	Down-regulated in PsA
RP11-68I18.2	0.0333267	1.14277	Up-regulated in PsA
RN7SL300P	0.0333303	1.35903	Up-regulated in PsA
STX18-IT1	0.0333335	1.49625	Up-regulated in PsA
SNORD88A	0.0333336	1.18234	Up-regulated in PsA
RP11-613M5.2	0.0333364	1.11122	Up-regulated in PsA

RP11-122C5.2	0.0333449	1.18138	Up-regulated in PsA
MIR654	0.0333529	1.27751	Up-regulated in PsA
TPD52L3	0.0333795	0.764217	Down-regulated in PsA
ZNF627	0.0333859	1.16945	Up-regulated in PsA
LOC102724264	0.0333879	1.27161	Up-regulated in PsA
PIK3IP1-AS1	0.033389	0.770218	Down-regulated in PsA
RP11-170N16.1	0.0334123	0.900287	Down-regulated in PsA
RP11-305L7.3	0.033447	1.30757	Up-regulated in PsA
DISC2	0.0334528	0.534228	Down-regulated in PsA
CTA-276O3.4	0.0334563	1.18185	Up-regulated in PsA
CDCA4	0.0334605	1.15513	Up-regulated in PsA
APOBEC1	0.0334798	1.42404	Up-regulated in PsA
PCMTD1	0.0334905	0.655904	Down-regulated in PsA
DCAF7	0.0335113	0.80636	Down-regulated in PsA
SCAMP5	0.0335117	1.37524	Up-regulated in PsA
Y_RNA	0.0335273	0.77067	Down-regulated in PsA
HSPE1P16	0.0335279	0.825767	Down-regulated in PsA
RP11-453N18.1	0.0335355	0.6924	Down-regulated in PsA
IL34	0.0335424	1.10746	Up-regulated in PsA
RCC2	0.0335572	0.887127	Down-regulated in PsA
FLG-AS1	0.0335834	0.809182	Down-regulated in PsA
RP11-241F15.5	0.0335952	1.134	Up-regulated in PsA
INSIG2	0.0336002	0.523545	Down-regulated in PsA
RP11-415C15.1	0.0336146	0.858145	Down-regulated in PsA
RPL23AP63	0.0336218	1.30062	Up-regulated in PsA

UGGT1	0.0336562	0.82359	Down-regulated in PsA
RP11-475A13.2	0.0337003	0.830198	Down-regulated in PsA
MGC16142	0.0337094	0.759407	Down-regulated in PsA
KRT17P5	0.0337113	1.17124	Up-regulated in PsA
RP11-1079K10.1	0.0337178	1.4187	Up-regulated in PsA
AFF2-IT1	0.0337209	1.12363	Up-regulated in PsA
RP11-383F6.1	0.0337212	0.885545	Down-regulated in PsA
RP11-338C15.2	0.0337443	1.40901	Up-regulated in PsA
AGAP13P	0.0337687	1.23558	Up-regulated in PsA
RN7SL362P	0.0338225	1.35585	Up-regulated in PsA
RP5-1077B9.5	0.0338332	1.16664	Up-regulated in PsA
ERMARD	0.0338392	0.61129	Down-regulated in PsA
PRSS29P	0.033841	1.3125	Up-regulated in PsA
ZNF33B	0.033849	0.717258	Down-regulated in PsA
RP11-626G11.1	0.0338494	0.907283	Down-regulated in PsA
LOC101928475	0.0338506	0.809671	Down-regulated in PsA
RP11-334E15.1	0.0338977	0.783096	Down-regulated in PsA
MTND2P22	0.0339258	1.30614	Up-regulated in PsA
CTD-2331H12.5	0.0339263	0.538511	Down-regulated in PsA
RN7SL484P	0.0339425	1.24401	Up-regulated in PsA
RBM44	0.0339469	1.28707	Up-regulated in PsA
EFCAB7	0.0339561	0.53028	Down-regulated in PsA
UBALD2	0.0339871	1.16944	Up-regulated in PsA
LOC101928371	0.0339969	0.851336	Down-regulated in PsA
DACH2	0.0340202	0.720064	Down-regulated in PsA

MROH2A	0.0341395	0.78921	Down-regulated in PsA
NDUFA5P4	0.0341614	1.14547	Up-regulated in PsA
MIR4462	0.0341921	0.693542	Down-regulated in PsA
TRIM46	0.034225	1.09183	Up-regulated in PsA
PRUNE	0.0342333	0.906033	Down-regulated in PsA
INPP5D	0.0342491	0.841161	Down-regulated in PsA
NCK1	0.0342585	0.682148	Down-regulated in PsA
NKRF	0.0343012	0.798459	Down-regulated in PsA
RP1-3J17.3	0.0343047	1.1102	Up-regulated in PsA
MTERF1	0.0343409	0.692154	Down-regulated in PsA
TMEM14EP	0.0343446	0.583037	Down-regulated in PsA
MROH1	0.0343666	1.08321	Up-regulated in PsA
MIR5088	0.0343746	1.0827	Up-regulated in PsA
RP11-337N6.1	0.0343777	1.13726	Up-regulated in PsA
RPS6KC1	0.0344008	0.73826	Down-regulated in PsA
RP11-757G14.3	0.0344023	1.11224	Up-regulated in PsA
RP11-757G14.3	0.0344023	1.11224	Up-regulated in PsA
RP11-613M5.1	0.0344177	0.778212	Down-regulated in PsA
MAGEB2	0.0344218	1.03298	Up-regulated in PsA
RP11-112N19.1	0.0344555	1.33336	Up-regulated in PsA
RN7SL464P	0.0344682	1.28113	Up-regulated in PsA
KCNG1	0.034473	0.784315	Down-regulated in PsA
ERN1	0.0344748	1.19033	Up-regulated in PsA
RN7SL397P	0.0345171	1.3971	Up-regulated in PsA
BIRC6-AS2	0.0345323	0.864056	Down-regulated in PsA

RP4-781K5.2	0.0345652	1.13092	Up-regulated in PsA
HOMER3	0.0345758	0.879374	Down-regulated in PsA
LLNLR-276E7.1	0.0345785	1.10127	Up-regulated in PsA
CTD-2249K22.1	0.0345815	1.61297	Up-regulated in PsA
LILRA6	0.0345865	0.806884	Down-regulated in PsA
HDX	0.034615	0.68155	Down-regulated in PsA
RP3-514P16.1	0.0346189	0.839939	Down-regulated in PsA
AC073063.10	0.0346467	0.65254	Down-regulated in PsA
RP11-517B11.6	0.0346868	1.28504	Up-regulated in PsA
TMEM54	0.0347083	1.12392	Up-regulated in PsA
RP11-122K13.7	0.034718	0.828156	Down-regulated in PsA
SRD5A3	0.0347243	0.765566	Down-regulated in PsA
RP11-798G7.8	0.0347253	0.660445	Down-regulated in PsA
LOC101928323	0.0347342	1.16114	Up-regulated in PsA
STARD7-AS1	0.034754	1.12772	Up-regulated in PsA
RP11-690C23.5	0.0347988	1.05736	Up-regulated in PsA
KRTAP10-7	0.0348261	1.30604	Up-regulated in PsA
ALDH3A2	0.0348378	0.823091	Down-regulated in PsA
RARS2	0.0349166	0.933271	Down-regulated in PsA
AP001059.5	0.0349173	1.19415	Up-regulated in PsA
FAM183CP	0.0349427	1.36673	Up-regulated in PsA
AC137934.1	0.0349478	0.776323	Down-regulated in PsA
CTD-2012I17.1	0.0349662	0.584195	Down-regulated in PsA
BHLHA9	0.0350084	0.879862	Down-regulated in PsA
FAM129A	0.0350103	0.772888	Down-regulated in PsA

HYPM	0.035016	0.928493	Down-regulated in PsA
CTD-2410N18.3	0.035025	1.03648	Up-regulated in PsA
CCL21	0.035054	1.40557	Up-regulated in PsA
CBX1	0.0351074	0.802936	Down-regulated in PsA
IFNW1	0.035133	1.2622	Up-regulated in PsA
CCDC153	0.035146	0.894497	Down-regulated in PsA
GMPPB	0.0351462	0.762754	Down-regulated in PsA
API5P1	0.0351464	0.668939	Down-regulated in PsA
AC011242.5	0.0352121	1.27992	Up-regulated in PsA
RP11-26L20.4	0.0352197	1.12595	Up-regulated in PsA
RP11-445P17.6	0.0352392	1.45903	Up-regulated in PsA
AC004129.9	0.0352621	1.10813	Up-regulated in PsA
CDC5L	0.0352833	0.698373	Down-regulated in PsA
MIR1228	0.0352879	1.19474	Up-regulated in PsA
LINC00506	0.0352915	1.17373	Up-regulated in PsA
RP11-432I5.4	0.0352926	0.737328	Down-regulated in PsA
RPL23AP44	0.0353015	1.16808	Up-regulated in PsA
SLFN12	0.035324	0.572376	Down-regulated in PsA
MTATP6P15	0.0353435	0.772443	Down-regulated in PsA
Metazoa_SRP	0.0353761	0.864471	Down-regulated in PsA
ASB5	0.0354539	1.15825	Up-regulated in PsA
C11orf54	0.0354574	0.747229	Down-regulated in PsA
AP000560.3	0.0354631	1.47547	Up-regulated in PsA
RP11-190D6.2	0.0354798	1.31212	Up-regulated in PsA
MIR4480	0.0355016	1.31034	Up-regulated in PsA

EFCAB13	0.0355017	2.04819	Up-regulated in PsA
RP13-347D8.1	0.0355291	0.85108	Down-regulated in PsA
MIR4746	0.0355302	1.20361	Up-regulated in PsA
CCIN	0.0355367	0.83452	Down-regulated in PsA
RP11-312A15.2	0.0356204	1.26887	Up-regulated in PsA
AC008060.5	0.035642	1.19449	Up-regulated in PsA
DCAF8L1	0.0356432	0.824748	Down-regulated in PsA
RP11-259O2.1	0.0356558	1.07908	Up-regulated in PsA
XRCC6P5	0.0357117	0.846163	Down-regulated in PsA
RP11-432M8.2	0.0357132	1.17162	Up-regulated in PsA
RP11-697E22.1	0.0357158	0.781726	Down-regulated in PsA
SERPINA6	0.035731	0.794916	Down-regulated in PsA
CYP2C61P	0.035738	0.673946	Down-regulated in PsA
AC093326.3	0.0357694	0.785678	Down-regulated in PsA
RP11-326E7.1	0.0357703	0.924814	Down-regulated in PsA
RP11-333A23.2	0.0357864	0.704892	Down-regulated in PsA
ELK3	0.0358054	0.779113	Down-regulated in PsA
DEFB104A	0.035807	1.11874	Up-regulated in PsA
RP11-684N3.1	0.0358149	1.27502	Up-regulated in PsA
RP11-89K18.1	0.0358209	1.12609	Up-regulated in PsA
QRFPR	0.0358283	0.855099	Down-regulated in PsA
APIP	0.0358544	0.683758	Down-regulated in PsA
RP5-1193P9.2	0.0358715	0.680693	Down-regulated in PsA
RP11-90P5.2	0.0358725	0.894098	Down-regulated in PsA
SNORA40	0.0358774	0.8348	Down-regulated in PsA

RP11-751K21.1	0.0358835	1.09722	Up-regulated in PsA
INPP4B	0.0358901	0.711149	Down-regulated in PsA
LINC00692	0.0359252	0.901072	Down-regulated in PsA
MIR329-1	0.0359269	1.51459	Up-regulated in PsA
FSHR	0.0359327	1.16423	Up-regulated in PsA
RP11-19P22.7	0.0359612	0.775298	Down-regulated in PsA
SNAR-A3	0.0359829	1.16163	Up-regulated in PsA
SNAR-A3	0.0359829	1.16163	Up-regulated in PsA
SNAR-A3	0.0359829	1.16163	Up-regulated in PsA
SNAR-A3	0.0359829	1.16163	Up-regulated in PsA
SNAR-A4	0.0359829	1.16163	Up-regulated in PsA
SNAR-A5	0.0359829	1.16163	Up-regulated in PsA
SNAR-A7	0.0359829	1.16163	Up-regulated in PsA
SNAR-A6	0.0359829	1.16163	Up-regulated in PsA
SNAR-A8	0.0359829	1.16163	Up-regulated in PsA
RNU6-45P	0.0360393	1.14133	Up-regulated in PsA
ZNF564	0.0360515	0.845244	Down-regulated in PsA
SNORA40	0.0360754	0.866823	Down-regulated in PsA
PRPH2	0.0360846	0.861925	Down-regulated in PsA
RTKN	0.0361046	1.13533	Up-regulated in PsA
RWDD3	0.0361065	0.800592	Down-regulated in PsA
RP3-399J4.3	0.0361255	1.3224	Up-regulated in PsA
PPBP	0.03613	4.53139	Up-regulated in PsA
RP5-945F2.2	0.0361352	1.09102	Up-regulated in PsA
RP5-1037N22.2	0.0361528	1.11018	Up-regulated in PsA

LOC101929261	0.0362125	1.19633	Up-regulated in PsA
SCN4B	0.0362327	0.853344	Down-regulated in PsA
RP3-441A12.1	0.0362435	0.681339	Down-regulated in PsA
LILRA4	0.036245	1.45462	Up-regulated in PsA
WDR12	0.0362458	0.541091	Down-regulated in PsA
SORBS3	0.0362501	0.781425	Down-regulated in PsA
AKIP1	0.0362625	0.816163	Down-regulated in PsA
CD276	0.0363278	0.90596	Down-regulated in PsA
OR2T11	0.0363345	1.18046	Up-regulated in PsA
ZNF787	0.0363358	1.1581	Up-regulated in PsA
SKA2	0.0363486	0.934373	Down-regulated in PsA
RNF38	0.0363494	1.21798	Up-regulated in PsA
RP11-69M1.3	0.0363601	0.838132	Down-regulated in PsA
DENND1B	0.0363659	0.772221	Down-regulated in PsA
LDB2	0.0363763	1.46343	Up-regulated in PsA
ARHGAP15	0.0363973	0.418178	Down-regulated in PsA
ZBTB40	0.0364218	0.816841	Down-regulated in PsA
LILRP2	0.0364284	0.713719	Down-regulated in PsA
TPRG1	0.0364432	0.454708	Down-regulated in PsA
AC010872.1	0.0364809	1.22806	Up-regulated in PsA
ZNF780A	0.0365172	0.707327	Down-regulated in PsA
STAU2-AS1	0.0365172	0.59981	Down-regulated in PsA
MIR5705	0.0365313	1.18242	Up-regulated in PsA
RP4-541C22.5	0.0365421	0.949013	Down-regulated in PsA
PDCL	0.0365431	0.870319	Down-regulated in PsA

AC090505.5	0.0365504	1.25503	Up-regulated in PsA
KCNC4-AS1	0.0365806	1.48288	Up-regulated in PsA
RP11-123O10.1	0.0365909	1.16506	Up-regulated in PsA
RN7SKP249	0.0365981	1.08019	Up-regulated in PsA
TACO1	0.036601	0.714487	Down-regulated in PsA
OR9G1	0.0366068	1.55151	Up-regulated in PsA
SMARCE1P4	0.0366226	1.23848	Up-regulated in PsA
RP11-64I5.1	0.036631	1.21654	Up-regulated in PsA
SLC50A1	0.0366361	1.1491	Up-regulated in PsA
LOC101928225	0.0366466	1.38422	Up-regulated in PsA
LINC00278	0.0366713	0.802727	Down-regulated in PsA
pRNA	0.036673	1.12081	Up-regulated in PsA
CYCSP5	0.0367043	0.664112	Down-regulated in PsA
RB1	0.0367168	0.801083	Down-regulated in PsA
SMARCAL1	0.0367217	0.619922	Down-regulated in PsA
LA16c-2F2.5	0.0367332	1.06198	Up-regulated in PsA
RP11-276H7.2	0.0367645	1.2161	Up-regulated in PsA
RP11-91A18.4	0.0367888	1.23442	Up-regulated in PsA
LOC102724539	0.0367966	1.33967	Up-regulated in PsA
AC068134.8	0.036799	0.731184	Down-regulated in PsA
RN7SKP235	0.0368574	1.29633	Up-regulated in PsA
RP11-423E7.1	0.0368585	1.41171	Up-regulated in PsA
CNTNAP3P2	0.0368736	0.84189	Down-regulated in PsA
SLC26A11	0.0368798	0.824562	Down-regulated in PsA
SMIM6	0.0368903	1.13271	Up-regulated in PsA

PDHX	0.0369721	0.646206	Down-regulated in PsA
SHH	0.036975	1.17271	Up-regulated in PsA
RPL23P11	0.0369897	1.5491	Up-regulated in PsA
DIS3L	0.0369908	0.819203	Down-regulated in PsA
CLCN5	0.0369925	0.733226	Down-regulated in PsA
UCA1	0.0370029	0.907474	Down-regulated in PsA
RP11-162N7.1	0.0370802	1.41341	Up-regulated in PsA
LINC01070	0.0371163	0.771344	Down-regulated in PsA
RP11-522B15.6	0.0371203	1.2361	Up-regulated in PsA
KRT17P4	0.037124	1.18579	Up-regulated in PsA
MTCO2P23	0.0371293	1.89595	Up-regulated in PsA
RP11-666A20.3	0.0371446	1.10195	Up-regulated in PsA
BNIP3P38	0.0371585	0.777573	Down-regulated in PsA
HNRNPA1P15	0.0371654	0.799977	Down-regulated in PsA
AAGAB	0.0371662	0.876318	Down-regulated in PsA
XXbac-BPG181B23.6	0.0371732	1.32064	Up-regulated in PsA
RP11-716D19.1	0.0372192	0.739561	Down-regulated in PsA
WBSCR28	0.0372245	0.810532	Down-regulated in PsA
CTB-129O4.1	0.0372398	1.27079	Up-regulated in PsA
ST20-MTHFS	0.0373558	0.722315	Down-regulated in PsA
C10orf90	0.0373607	0.605212	Down-regulated in PsA
RP11-82O19.1	0.0373798	1.13991	Up-regulated in PsA
RP11-553L6.2	0.037389	1.49869	Up-regulated in PsA
AKNAD1	0.0373955	0.893662	Down-regulated in PsA
LINGO1	0.037444	1.07977	Up-regulated in PsA

RP11-362K14.7	0.0374512	1.2267 Up-regulated in PsA
PRKD3	0.0374551	0.765009 Down-regulated in PsA
UBTF	0.0374853	0.860412 Down-regulated in PsA
AC016735.1	0.0375036	0.976342 Down-regulated in PsA
RP11-142O6.1	0.0375534	1.72022 Up-regulated in PsA
SMKR1	0.0375677	0.851924 Down-regulated in PsA
BIK	0.0375872	1.3217 Up-regulated in PsA
MED28P7	0.0375971	0.868292 Down-regulated in PsA
SLC25A24	0.037602	0.675654 Down-regulated in PsA
RPS7P7	0.0376036	0.827875 Down-regulated in PsA
ATG101	0.0376058	0.804784 Down-regulated in PsA
Y_RNA	0.0376192	1.17003 Up-regulated in PsA
TMEM55A	0.0376266	0.755625 Down-regulated in PsA
RAB8A	0.0376384	0.914253 Down-regulated in PsA
XXbac-B444P24.14	0.0376572	1.18736 Up-regulated in PsA
MIR4283-2	0.0376621	0.888333 Down-regulated in PsA
ARL6IP1	0.0376626	0.855527 Down-regulated in PsA
FPGT	0.0376787	0.50427 Down-regulated in PsA
CPLX3	0.0376915	1.10589 Up-regulated in PsA
RP11-636N17.1	0.0376981	0.870514 Down-regulated in PsA
BLM	0.0377007	0.740259 Down-regulated in PsA
VKORC1L1	0.0377122	0.740304 Down-regulated in PsA
HAS1	0.0377399	1.39336 Up-regulated in PsA
ZNF484	0.0377674	0.837318 Down-regulated in PsA
JAGN1	0.0377789	0.837914 Down-regulated in PsA

TMEM178A	0.0377913	0.780666	Down-regulated in PsA
LRPPRC	0.0378322	0.803524	Down-regulated in PsA
NPTN-IT1	0.037833	0.711858	Down-regulated in PsA
TERT	0.0378559	1.16165	Up-regulated in PsA
RN7SL83P	0.0378798	1.05986	Up-regulated in PsA
RP11-609N14.4	0.0378861	1.17735	Up-regulated in PsA
MINA	0.0379202	0.761075	Down-regulated in PsA
RP11-452L6.5	0.0379674	1.15174	Up-regulated in PsA
HGFAC	0.0379722	0.827451	Down-regulated in PsA
CYP4A22	0.03798	1.33657	Up-regulated in PsA
FBXO45	0.037981	0.566868	Down-regulated in PsA
RP11-4M23.3	0.0379907	0.90936	Down-regulated in PsA
PLEKHA8	0.0379948	0.864241	Down-regulated in PsA
API5P2	0.0379966	0.793871	Down-regulated in PsA
FHL5	0.0380089	1.04806	Up-regulated in PsA
FAM86C2P	0.0380195	1.25346	Up-regulated in PsA
TRAPPC2L	0.0380442	0.737323	Down-regulated in PsA
LOC100505658	0.0380922	1.31567	Up-regulated in PsA
LYAR	0.0381005	0.609435	Down-regulated in PsA
GS1-279B7.2	0.038108	0.758162	Down-regulated in PsA
FGFRL1	0.0381087	0.817604	Down-regulated in PsA
RP11-255G12.2	0.0381093	1.23604	Up-regulated in PsA
SPHK2	0.0381161	0.913409	Down-regulated in PsA
WFDC3	0.038137	1.08871	Up-regulated in PsA
TMEM107	0.0381576	0.819865	Down-regulated in PsA

RBM27	0.0381905	0.828562	Down-regulated in PsA
CDK2AP1	0.0381961	0.829119	Down-regulated in PsA
Y_RNA	0.0381997	0.710288	Down-regulated in PsA
MIR203A	0.0382084	0.775436	Down-regulated in PsA
LINC00622	0.0382167	0.757911	Down-regulated in PsA
NTS	0.0382257	0.7376	Down-regulated in PsA
RP5-856G1.1	0.0382467	1.31463	Up-regulated in PsA
RP11-536P16.2	0.0382618	1.36559	Up-regulated in PsA
RP11-666E17.1	0.0383155	1.56153	Up-regulated in PsA
NUBP1	0.0383357	0.761193	Down-regulated in PsA
RN7SL340P	0.0384147	1.09326	Up-regulated in PsA
FUK	0.038432	0.785462	Down-regulated in PsA
POLQ	0.0384368	0.511374	Down-regulated in PsA
GALNTL5	0.0384534	0.635781	Down-regulated in PsA
IGBP1P4	0.0384973	1.18796	Up-regulated in PsA
ZNF205	0.0385187	0.792162	Down-regulated in PsA
ECI2	0.0385245	0.749065	Down-regulated in PsA
TMEM151B	0.0385257	1.20236	Up-regulated in PsA
ACOX2	0.0385664	1.36287	Up-regulated in PsA
AC113618.2	0.0385673	1.40879	Up-regulated in PsA
KLHL30	0.0385725	0.756761	Down-regulated in PsA
GRB14	0.0385785	0.824645	Down-regulated in PsA
NXPE4	0.0386206	0.740751	Down-regulated in PsA
TIMMDC1	0.0386259	0.778631	Down-regulated in PsA
POLR2J3	0.0387103	1.16214	Up-regulated in PsA

Y_RNA	0.0387294	0.924905	Down-regulated in PsA
TMEM106A	0.0387415	0.602046	Down-regulated in PsA
OR13F1	0.0387603	0.69956	Down-regulated in PsA
BAG2	0.0387787	0.484624	Down-regulated in PsA
RP11-861L17.2	0.0387973	1.34262	Up-regulated in PsA
CTB-57H20.1	0.0388053	0.60382	Down-regulated in PsA
NBN	0.0388164	0.744317	Down-regulated in PsA
SH2D5	0.0388176	1.19231	Up-regulated in PsA
RP11-432M8.11	0.0388513	1.42744	Up-regulated in PsA
RP1-251M9.2	0.0389058	1.36541	Up-regulated in PsA
PRR29	0.0389211	0.829447	Down-regulated in PsA
AMMECR1	0.0389225	0.921242	Down-regulated in PsA
RP11-476J6.1	0.0389288	0.667343	Down-regulated in PsA
ERCC4	0.0389565	0.5603	Down-regulated in PsA
RP11-413M3.4	0.0389724	1.32971	Up-regulated in PsA
C18orf12	0.038983	1.23297	Up-regulated in PsA
PSMC1P13	0.0389994	1.18488	Up-regulated in PsA
MTND3P17	0.0390074	0.85957	Down-regulated in PsA
RP11-102F4.2	0.0390271	1.19135	Up-regulated in PsA
NT5DC1	0.0391153	0.868158	Down-regulated in PsA
GABRE	0.0391172	1.39163	Up-regulated in PsA
PLXDC2	0.0391324	0.920773	Down-regulated in PsA
LOC101927292	0.0391464	0.858861	Down-regulated in PsA
MIR328	0.0391631	1.15179	Up-regulated in PsA
MYL12B	0.0391858	0.881678	Down-regulated in PsA

COX6B1P2	0.0392213	1.18362	Up-regulated in PsA
GJA6P	0.0392464	0.562336	Down-regulated in PsA
TLR10	0.0392684	0.491266	Down-regulated in PsA
RP11-420K8.1	0.0393005	0.65256	Down-regulated in PsA
LINC00652	0.0393051	0.678946	Down-regulated in PsA
RP11-74H8.1	0.0393284	1.19622	Up-regulated in PsA
RP11-96K19.2	0.0393367	0.765307	Down-regulated in PsA
RHOT2	0.0393646	1.12327	Up-regulated in PsA
RP11-455O6.2	0.0393841	1.42145	Up-regulated in PsA
TSPY1	0.0393857	0.78078	Down-regulated in PsA
NUS1P1	0.0393965	0.84684	Down-regulated in PsA
GOT2P5	0.0394335	1.28943	Up-regulated in PsA
NR2F6	0.0394797	1.29485	Up-regulated in PsA
C4orf32	0.0395189	0.868704	Down-regulated in PsA
TES	0.0395451	0.782409	Down-regulated in PsA
RN7SL452P	0.0396032	1.47027	Up-regulated in PsA
MYOF	0.0396447	0.583242	Down-regulated in PsA
DCAF13P3	0.0396581	0.687675	Down-regulated in PsA
RP11-239E10.3	0.0396867	0.889898	Down-regulated in PsA
HOXD-AS2	0.039713	1.30174	Up-regulated in PsA
RP11-666A1.4	0.0397405	1.36738	Up-regulated in PsA
DLGAP3	0.0397513	0.704752	Down-regulated in PsA
RP11-342K6.2	0.0397597	0.653239	Down-regulated in PsA
RP11-219I21.1	0.0397896	1.24181	Up-regulated in PsA
RAB27A	0.0397933	0.766708	Down-regulated in PsA

ARHGAP19-SLIT1	0.0397987	0.781832	Down-regulated in PsA
HELLS	0.0398404	1.25305	Up-regulated in PsA
FOXI1	0.0398489	0.907273	Down-regulated in PsA
RP11-428G2.1	0.0398997	0.729951	Down-regulated in PsA
ZNF546	0.0398998	0.931738	Down-regulated in PsA
AC007879.6	0.0399109	1.42783	Up-regulated in PsA
EIF3EP2	0.0399132	1.62409	Up-regulated in PsA
RP11-243J16.8	0.0399224	1.18259	Up-regulated in PsA
ENAH	0.0399505	1.41075	Up-regulated in PsA
RP11-15G8.1	0.039982	1.53449	Up-regulated in PsA
RP1-90J20.2	0.0400238	1.39544	Up-regulated in PsA
CES5AP1	0.0400753	1.28599	Up-regulated in PsA
TIGIT	0.0400803	1.38548	Up-regulated in PsA
LVRN	0.0401106	0.774401	Down-regulated in PsA
FAM127B	0.0401208	0.870488	Down-regulated in PsA
TTC33	0.0401801	0.467688	Down-regulated in PsA
PYGM	0.0401853	1.18099	Up-regulated in PsA
C10orf111	0.0401871	1.43967	Up-regulated in PsA
MZB1	0.0401913	1.68284	Up-regulated in PsA
HSPA8	0.0402059	0.845128	Down-regulated in PsA
RP11-536C10.10	0.0403203	1.15674	Up-regulated in PsA
C17orf77	0.0403485	1.2512	Up-regulated in PsA
RP11-113K21.1	0.0403505	1.23149	Up-regulated in PsA
B3GNT7	0.0403911	0.780481	Down-regulated in PsA
SNORD38	0.0403915	0.750388	Down-regulated in PsA

STAM	0.0404085	0.820643	Down-regulated in PsA
MIR103B2	0.0404117	0.810463	Down-regulated in PsA
DLX3	0.0405341	0.90549	Down-regulated in PsA
KCTD15	0.0405721	1.0218	Up-regulated in PsA
EIF4E2	0.0406009	0.892851	Down-regulated in PsA
MIR4316	0.0406383	1.17502	Up-regulated in PsA
CTC-436K13.6	0.0406539	1.63336	Up-regulated in PsA
MIR4774	0.0406543	0.692692	Down-regulated in PsA
RP11-359P18.5	0.0406586	1.48595	Up-regulated in PsA
NVL	0.0406709	0.751349	Down-regulated in PsA
IFNL4P1	0.040715	1.14674	Up-regulated in PsA
IFITM1	0.0407218	1.3716	Up-regulated in PsA
RP11-325O24.5	0.0407632	1.44766	Up-regulated in PsA
TONSL	0.040833	0.823483	Down-regulated in PsA
HSPA1L	0.0408776	0.869676	Down-regulated in PsA
ZNF786	0.0408944	0.760729	Down-regulated in PsA
PLSCR4	0.0409321	0.695669	Down-regulated in PsA
MTCO3P17	0.0409717	0.867943	Down-regulated in PsA
LOC100128554	0.0409739	1.44067	Up-regulated in PsA
TMEM185A	0.0409752	0.845585	Down-regulated in PsA
ATP8B1	0.0410065	0.489571	Down-regulated in PsA
CELF1	0.041026	0.895449	Down-regulated in PsA
CTD-2013N17.6	0.0410345	0.791349	Down-regulated in PsA
NPY	0.0410559	1.06896	Up-regulated in PsA
DTX2	0.0410804	1.08811	Up-regulated in PsA

RP11-240G22.1	0.0410955	0.811706	Down-regulated in PsA
RP11-225B17.1	0.0411084	0.760168	Down-regulated in PsA
ST20-AS1	0.041132	0.792106	Down-regulated in PsA
CDON	0.0411333	0.641965	Down-regulated in PsA
C18orf42	0.0411589	0.715935	Down-regulated in PsA
TSPY14P	0.0411703	1.23923	Up-regulated in PsA
MIR3692	0.0411762	1.61451	Up-regulated in PsA
MIR523	0.0411888	1.40728	Up-regulated in PsA
ZSCAN2	0.0412108	0.796817	Down-regulated in PsA
RN7SL845P	0.0412399	1.17775	Up-regulated in PsA
YAE1D1	0.0412609	0.415976	Down-regulated in PsA
RP11-666O2.5	0.0412665	1.10931	Up-regulated in PsA
RP11-129B9.1	0.041273	0.962286	Down-regulated in PsA
RP11-1399P15.1	0.041314	0.825979	Down-regulated in PsA
VTRNA1-3	0.0413143	1.42876	Up-regulated in PsA
HIF1AP1	0.0413197	1.4847	Up-regulated in PsA
LINC01021	0.0413725	0.856742	Down-regulated in PsA
RP11-236J17.6	0.0413774	0.783512	Down-regulated in PsA
SLC39A11	0.0413851	0.852239	Down-regulated in PsA
RP11-737O24.5	0.0414037	1.27219	Up-regulated in PsA
MIR6068	0.041439	1.16991	Up-regulated in PsA
TFF3	0.0414537	0.809001	Down-regulated in PsA
AC083875.2	0.0414561	0.799365	Down-regulated in PsA
Y_RNA	0.0415042	1.27859	Up-regulated in PsA
Metazoa_SRP	0.0415226	1.41453	Up-regulated in PsA

RP11-449P1.1	0.041548	1.09686	Up-regulated in PsA
NRN1L	0.0415521	1.18582	Up-regulated in PsA
RP11-571M6.18	0.0415817	1.09394	Up-regulated in PsA
TYW1B	0.0415959	0.655241	Down-regulated in PsA
TCEB1P30	0.0416125	0.768971	Down-regulated in PsA
KIAA2022	0.0416434	0.527109	Down-regulated in PsA
LCN12	0.041672	0.802293	Down-regulated in PsA
RP11-989E6.3	0.0416803	1.22631	Up-regulated in PsA
SLIT1-AS1	0.0417173	1.54009	Up-regulated in PsA
TCP1	0.0417218	0.799676	Down-regulated in PsA
RN7SL834P	0.0417558	0.884299	Down-regulated in PsA
ARHGEF39	0.0417723	0.67165	Down-regulated in PsA
RP5-1163L11.2	0.0418073	1.31571	Up-regulated in PsA
BIRC6	0.0418337	0.829271	Down-regulated in PsA
OR4E1	0.0418358	1.03492	Up-regulated in PsA
RPL21P112	0.041866	0.716047	Down-regulated in PsA
BTBD3	0.0418739	0.842632	Down-regulated in PsA
DFFA	0.0418897	0.703108	Down-regulated in PsA
RP11-315I20.1	0.0419027	1.30345	Up-regulated in PsA
AC006445.7	0.0419123	1.11773	Up-regulated in PsA
CHST15	0.0419161	0.771654	Down-regulated in PsA
MYCNOS	0.0419384	1.3062	Up-regulated in PsA
POLR3GP2	0.0419418	1.22303	Up-regulated in PsA
RP5-919F19.5	0.0419557	1.4853	Up-regulated in PsA
AC092071.1	0.0419657	1.23972	Up-regulated in PsA

YBEY	0.0419667	0.296686	Down-regulated in PsA
EPHB1	0.0420257	1.37569	Up-regulated in PsA
LOC101929723	0.0420288	0.83993	Down-regulated in PsA
TNS2	0.0420527	1.10864	Up-regulated in PsA
GNB3	0.0420545	1.05382	Up-regulated in PsA
MSH2	0.0420625	0.49034	Down-regulated in PsA
ADIPOQ	0.042106	0.896843	Down-regulated in PsA
RPE65	0.0421174	0.749081	Down-regulated in PsA
IQSEC2	0.0421539	0.935876	Down-regulated in PsA
PLXNC1	0.0421679	0.816225	Down-regulated in PsA
RNA5SP484	0.0422561	1.34866	Up-regulated in PsA
RP11-803D5.1	0.0422818	0.538036	Down-regulated in PsA
RIF1	0.0423357	1.31662	Up-regulated in PsA
PSMD2	0.0423367	0.798864	Down-regulated in PsA
DIDO1	0.0423433	0.885964	Down-regulated in PsA
TIAL1	0.0423566	0.884024	Down-regulated in PsA
RP11-17G12.2	0.0423675	1.05013	Up-regulated in PsA
RP11-56B16.5	0.0423751	1.3179	Up-regulated in PsA
RN7SL538P	0.0423863	1.5004	Up-regulated in PsA
RP11-182E7.1	0.0424214	1.15443	Up-regulated in PsA
EIF1AD	0.0424453	0.821971	Down-regulated in PsA
MAP2K6	0.0424516	0.700403	Down-regulated in PsA
RN7SL314P	0.042476	1.1985	Up-regulated in PsA
ABCE1	0.0425862	0.763275	Down-regulated in PsA
TCF12	0.0426052	0.750535	Down-regulated in PsA

RP11-521B24.5	0.0426116	1.23872 Up-regulated in PsA
RP11-471B22.2	0.0426636	0.950337 Down-regulated in PsA
BBOX1	0.0426717	1.31701 Up-regulated in PsA
AL050303.10	0.0426799	1.40207 Up-regulated in PsA
RP11-88I18.2	0.0426824	0.450051 Down-regulated in PsA
XKR8	0.0426988	1.21121 Up-regulated in PsA
MOXD1	0.0427049	1.26167 Up-regulated in PsA
RP1-131F15.2	0.0427875	1.23156 Up-regulated in PsA
SPEF1	0.0428185	0.754548 Down-regulated in PsA
MTCO1P28	0.0428656	1.32937 Up-regulated in PsA
Y_RNA	0.0428949	1.19364 Up-regulated in PsA
EIF2B4	0.0429006	0.863847 Down-regulated in PsA
MIR320B1	0.0429021	1.5184 Up-regulated in PsA
RP11-18O11.1	0.0429098	1.30044 Up-regulated in PsA
RP11-467P9.1	0.0429298	1.08503 Up-regulated in PsA
MIR4520-2	0.0429781	0.838398 Down-regulated in PsA
TMEM216	0.0429939	0.853228 Down-regulated in PsA
CBR3	0.0430077	0.66133 Down-regulated in PsA
C11orf57	0.0430093	0.706504 Down-regulated in PsA
ADPRH	0.0430095	0.807917 Down-regulated in PsA
FTH1P12	0.0430221	1.08309 Up-regulated in PsA
CTD-2620I22.2	0.0430221	0.874252 Down-regulated in PsA
LINC01571	0.0430289	0.761442 Down-regulated in PsA
TDO2	0.0430335	0.656721 Down-regulated in PsA
HNCAT21	0.0430396	1.21111 Up-regulated in PsA

Y_RNA	0.043056	1.06708	Up-regulated in PsA
GABRQ	0.043084	0.750789	Down-regulated in PsA
RP11-343D2.11	0.0430893	0.736209	Down-regulated in PsA
RN7SL513P	0.0431073	1.4155	Up-regulated in PsA
RP11-1060G2.2	0.0431246	1.28688	Up-regulated in PsA
SNORD99	0.0431773	1.23466	Up-regulated in PsA
LOC100287632	0.0431892	0.781942	Down-regulated in PsA
XPA	0.0432098	0.701987	Down-regulated in PsA
RP11-545A16.3	0.0432119	1.11336	Up-regulated in PsA
IDO1	0.043219	0.869346	Down-regulated in PsA
ARNTL	0.0432254	0.801228	Down-regulated in PsA
PKM	0.043228	0.886129	Down-regulated in PsA
WI2-88277B6.1	0.0432449	1.13436	Up-regulated in PsA
CC2D2B	0.0432573	0.49821	Down-regulated in PsA
IGBP1-AS1	0.0433002	0.67492	Down-regulated in PsA
MRPS28	0.043371	0.670346	Down-regulated in PsA
CTD-3193K9.3	0.0433764	0.67312	Down-regulated in PsA
ALS2CL	0.0433791	0.823267	Down-regulated in PsA
FOXJ1	0.0433852	1.18553	Up-regulated in PsA
ARL2BP	0.0434055	0.814123	Down-regulated in PsA
RP11-805F19.3	0.0434542	1.17231	Up-regulated in PsA
LGALS13	0.0434645	0.777559	Down-regulated in PsA
RP3-521E19.3	0.0434706	1.14245	Up-regulated in PsA
OR4X2	0.0434755	0.838863	Down-regulated in PsA
LINC00336	0.0434802	1.27899	Up-regulated in PsA

RP11-857B24.1	0.0434903	1.07578	Up-regulated in PsA
POT1	0.0434923	0.719305	Down-regulated in PsA
USP17L3	0.0434959	0.75065	Down-regulated in PsA
SCG5	0.0435159	1.21333	Up-regulated in PsA
FOXD4L5	0.0435273	0.817762	Down-regulated in PsA
RPL31P15	0.0435305	0.766325	Down-regulated in PsA
RPS8P6	0.0435409	1.12997	Up-regulated in PsA
AC006026.13	0.0435421	0.799596	Down-regulated in PsA
C15orf62	0.0435908	0.699108	Down-regulated in PsA
Metazoa_SRP	0.0435967	1.34684	Up-regulated in PsA
MIR4452	0.0436074	1.43023	Up-regulated in PsA
RP11-660L16.2	0.0436076	1.28853	Up-regulated in PsA
FAM174B	0.0436522	0.893177	Down-regulated in PsA
KRT8P29	0.0436688	1.56961	Up-regulated in PsA
OSGEP	0.043684	0.806287	Down-regulated in PsA
AC009237.16	0.0437039	1.35544	Up-regulated in PsA
LINC00909	0.0437358	0.691776	Down-regulated in PsA
LRIG1	0.043751	0.853764	Down-regulated in PsA
TMEM165	0.0437572	0.635082	Down-regulated in PsA
HDDC2	0.0437581	0.722591	Down-regulated in PsA
PPHLN1	0.04376	0.900165	Down-regulated in PsA
RP11-655G22.2	0.0437608	1.3061	Up-regulated in PsA
RP11-24J19.1	0.0437748	1.06822	Up-regulated in PsA
SSU72P1	0.04378	0.662723	Down-regulated in PsA
STK19	0.0437861	0.795134	Down-regulated in PsA

RBPJP2	0.0437947	0.729923	Down-regulated in PsA
LOC728024	0.0438117	0.68354	Down-regulated in PsA
RPS4XP4	0.0438546	1.35721	Up-regulated in PsA
DGKD	0.0438934	0.767928	Down-regulated in PsA
CTC-480C2.1	0.0438947	1.25841	Up-regulated in PsA
CDKN2B-AS1	0.0439001	0.676653	Down-regulated in PsA
AP1AR	0.0439217	0.624806	Down-regulated in PsA
RP11-309M23.1	0.0439263	0.732353	Down-regulated in PsA
RP11-309M23.1	0.0439263	0.732353	Down-regulated in PsA
TNFRSF13B	0.0439299	1.03286	Up-regulated in PsA
RP11-432M8.4	0.0439409	1.23137	Up-regulated in PsA
EDAR	0.043941	1.07753	Up-regulated in PsA
PRORY	0.0439493	0.759397	Down-regulated in PsA
CICP10	0.0439748	1.16576	Up-regulated in PsA
RP11-569G9.7	0.0439801	0.802539	Down-regulated in PsA
RP11-462L8.2	0.043984	0.872386	Down-regulated in PsA
SPAG16	0.043985	0.881294	Down-regulated in PsA
RP11-109M19.1	0.0439902	1.4593	Up-regulated in PsA
RPS27P22	0.0440264	1.19604	Up-regulated in PsA
UBE2J2	0.0440438	0.750234	Down-regulated in PsA
LINC01572	0.0440586	1.29033	Up-regulated in PsA
SRP14-AS1	0.0440777	0.637111	Down-regulated in PsA
RN7SL756P	0.0441002	1.27958	Up-regulated in PsA
EPRS	0.0441325	0.767188	Down-regulated in PsA
MEAF6P1	0.0441348	1.23138	Up-regulated in PsA

RP11-452G18.1	0.0441762	0.789995	Down-regulated in PsA
LOC101927915	0.0442082	0.825059	Down-regulated in PsA
RP11-312A15.3	0.0442193	1.10293	Up-regulated in PsA
LOC102724502	0.0442298	1.2186	Up-regulated in PsA
MFHAS1	0.0442598	0.837865	Down-regulated in PsA
RP11-291L22.4	0.0442611	0.782562	Down-regulated in PsA
RP11-316J7.4	0.0442668	1.46498	Up-regulated in PsA
CTD-3064H18.4	0.0442676	1.3753	Up-regulated in PsA
CYCSP27	0.044315	0.730553	Down-regulated in PsA
CSPG4P1Y	0.0443328	1.70263	Up-regulated in PsA
RP11-332J15.3	0.0443652	1.16619	Up-regulated in PsA
RP11-5P18.11	0.0443715	1.09266	Up-regulated in PsA
ARL14EPL	0.0443716	0.760522	Down-regulated in PsA
PQLC2	0.044373	0.75672	Down-regulated in PsA
RP11-275I4.2	0.044388	0.704935	Down-regulated in PsA
TAL2	0.0444149	0.698817	Down-regulated in PsA
KDM4A-AS1	0.0444244	0.909005	Down-regulated in PsA
LOC645513	0.0444354	0.841026	Down-regulated in PsA
PCAT14	0.0444358	1.24917	Up-regulated in PsA
CYP4F8	0.0444453	0.915337	Down-regulated in PsA
RP11-575C1.1	0.0444637	0.72765	Down-regulated in PsA
DDX39BP2	0.0444866	0.700923	Down-regulated in PsA
RP11-813B8.1	0.0445204	1.15556	Up-regulated in PsA
HYAL3	0.0445319	0.606236	Down-regulated in PsA
RP11-358D17.1	0.0445638	1.15798	Up-regulated in PsA

ZNF568	0.0445844	0.645403	Down-regulated in PsA
FOXO6	0.0445918	1.1657	Up-regulated in PsA
DDX55	0.0447003	0.916156	Down-regulated in PsA
CAPN1	0.0447247	0.935332	Down-regulated in PsA
LINC00463	0.044772	1.2467	Up-regulated in PsA
RP11-814H16.2	0.0447805	0.844357	Down-regulated in PsA
RPS17P11	0.04479	1.30166	Up-regulated in PsA
H19_3	0.0447981	0.797057	Down-regulated in PsA
NIPA2	0.0448349	1.29299	Up-regulated in PsA
HRK	0.0448435	0.910863	Down-regulated in PsA
ZBED5-AS1	0.0448682	0.673125	Down-regulated in PsA
MORC2	0.0448998	0.85425	Down-regulated in PsA
PRPF38B	0.0449274	0.826173	Down-regulated in PsA
INSR	0.0449552	0.78806	Down-regulated in PsA
MTG2	0.0449578	0.802245	Down-regulated in PsA
RP11-735A19.2	0.0449595	1.21217	Up-regulated in PsA
GSTO2	0.0449912	0.854778	Down-regulated in PsA
GAS5-AS1	0.045012	0.476191	Down-regulated in PsA
TAF15	0.0450143	0.910167	Down-regulated in PsA
POU2F1	0.0450603	0.755019	Down-regulated in PsA
SSU72P3	0.0450848	1.35072	Up-regulated in PsA
PHBP8	0.0450921	0.837559	Down-regulated in PsA
RP11-301G19.1	0.0451037	1.18124	Up-regulated in PsA
RP11-335K5.2	0.0451398	1.12941	Up-regulated in PsA
RP1-130G2.1	0.0451715	0.880288	Down-regulated in PsA

NLK	0.0451718	0.787657	Down-regulated in PsA
TMPO-AS1	0.0452041	0.822038	Down-regulated in PsA
SURF4	0.0452137	0.870432	Down-regulated in PsA
RP11-79P5.3	0.0452452	1.08995	Up-regulated in PsA
RP11-300A12.2	0.0452489	0.688773	Down-regulated in PsA
BLOC1S2	0.0452542	0.794096	Down-regulated in PsA
FLG-AS1	0.0452778	1.36952	Up-regulated in PsA
RP11-483G21.3	0.0453144	1.08249	Up-regulated in PsA
LINC01143	0.0453703	1.23966	Up-regulated in PsA
NUP50	0.045409	0.6174	Down-regulated in PsA
OR4S1	0.0454094	1.08579	Up-regulated in PsA
ZNF860	0.045431	0.828176	Down-regulated in PsA
RIF1	0.0454323	0.747668	Down-regulated in PsA
RP11-556H2.2	0.0454599	0.617361	Down-regulated in PsA
RNF41	0.0454614	0.84845	Down-regulated in PsA
SETD6P1	0.0454928	0.752541	Down-regulated in PsA
RP11-700H6.2	0.0454932	0.782272	Down-regulated in PsA
ZNF35	0.0455028	0.731868	Down-regulated in PsA
RP11-457P14.6	0.0455391	0.77687	Down-regulated in PsA
RP11-574K11.8	0.0455606	0.688416	Down-regulated in PsA
RP11-634H22.1	0.045564	1.2079	Up-regulated in PsA
SLC26A1	0.0455847	1.08612	Up-regulated in PsA
RP11-403P14.1	0.0456263	0.661096	Down-regulated in PsA
TARDBPP2	0.0456269	0.829023	Down-regulated in PsA
HMCN2	0.045637	0.818177	Down-regulated in PsA

AMDHD1	0.0456493	0.879505	Down-regulated in PsA
RN7SL113P	0.0456525	1.1382	Up-regulated in PsA
CARD16	0.0456626	0.826278	Down-regulated in PsA
SLC30A2	0.0456755	1.11985	Up-regulated in PsA
TMEM202	0.045712	0.852313	Down-regulated in PsA
PXK	0.0457197	0.914961	Down-regulated in PsA
SRGAP2D	0.0457304	0.866789	Down-regulated in PsA
LOC102724200	0.0457319	0.777412	Down-regulated in PsA
NR5A1	0.0457395	0.690137	Down-regulated in PsA
AC113167.2	0.0457494	0.845925	Down-regulated in PsA
PACRG-AS3	0.0457897	0.79759	Down-regulated in PsA
BTN2A3P	0.0458001	0.775969	Down-regulated in PsA
PRRC2CP1	0.0458265	0.772977	Down-regulated in PsA
MACROD2-IT1	0.045842	1.19691	Up-regulated in PsA
C1orf109	0.0458634	0.848073	Down-regulated in PsA
RN7SL79P	0.0458851	1.40636	Up-regulated in PsA
ZNF132	0.0458894	0.627574	Down-regulated in PsA
CTB-33O18.2	0.0459138	0.761303	Down-regulated in PsA
SDHDP1	0.0459557	0.804428	Down-regulated in PsA
RP11-174O3.3	0.0459749	0.647851	Down-regulated in PsA
RP11-521C20.3	0.0459982	0.72081	Down-regulated in PsA
KCNA6	0.0460471	1.2519	Up-regulated in PsA
ERP29	0.046063	0.792474	Down-regulated in PsA
STK32C	0.0460691	0.756166	Down-regulated in PsA
RAB2B	0.0460821	0.726235	Down-regulated in PsA

CDY2A	0.0460925	1.28608	Up-regulated in PsA
RP11-419L4.1	0.0461204	0.797195	Down-regulated in PsA
TAF1D	0.0461349	0.777175	Down-regulated in PsA
WDR33	0.0461901	0.859339	Down-regulated in PsA
RP11-59H7.1	0.0461987	1.18223	Up-regulated in PsA
VPS50	0.0462003	0.649536	Down-regulated in PsA
AC005625.1	0.0462337	1.39621	Up-regulated in PsA
MCM10	0.0462348	0.716286	Down-regulated in PsA
LILRB4	0.0462366	0.693579	Down-regulated in PsA
CBX3P9	0.0463068	0.747957	Down-regulated in PsA
CTC-338M12.1	0.0463095	1.17791	Up-regulated in PsA
RFC2	0.0463431	0.748393	Down-regulated in PsA
KEAP1	0.0463475	0.787989	Down-regulated in PsA
RP11-196G18.1	0.0463492	1.28577	Up-regulated in PsA
RP11-327F22.6	0.0463671	0.634811	Down-regulated in PsA
ZNF502	0.0463871	0.623741	Down-regulated in PsA
SNORD115-48	0.0464005	1.22965	Up-regulated in PsA
AC006942.4	0.0464336	1.10561	Up-regulated in PsA
HMGB1P19	0.0464614	0.660718	Down-regulated in PsA
LOC101927637	0.0464815	0.818499	Down-regulated in PsA
LRMP	0.0464829	0.879556	Down-regulated in PsA
RPS15AP27	0.0464836	0.792324	Down-regulated in PsA
CTB-164N12.1	0.0464874	0.688946	Down-regulated in PsA
RP11-277A4.4	0.0464987	1.53174	Up-regulated in PsA
SLC37A3	0.0465542	1.29882	Up-regulated in PsA

TAS2R13	0.0465637	1.15289	Up-regulated in PsA
CA2	0.0465769	0.590866	Down-regulated in PsA
POLR3A	0.0466138	0.684474	Down-regulated in PsA
MUC13	0.0466384	1.24603	Up-regulated in PsA
PRKY	0.04664	0.766577	Down-regulated in PsA
TINF2	0.0466437	0.86253	Down-regulated in PsA
RP4-614N24.1	0.0466508	0.743064	Down-regulated in PsA
RIBC1	0.0466512	1.05111	Up-regulated in PsA
RP11-596D21.1	0.0466601	0.840672	Down-regulated in PsA
PCGEM1	0.046676	0.775562	Down-regulated in PsA
ARL2-SNX15	0.0466919	0.859722	Down-regulated in PsA
RP11-142G1.3	0.0467318	1.50001	Up-regulated in PsA
C10orf53	0.0467601	0.633605	Down-regulated in PsA
C2orf42	0.0467633	0.689628	Down-regulated in PsA
GUCY2GP	0.0467643	1.30926	Up-regulated in PsA
RN7SL796P	0.046772	1.19459	Up-regulated in PsA
CARD17	0.046778	0.78939	Down-regulated in PsA
MIR4468	0.0468051	1.24703	Up-regulated in PsA
TMF1P1	0.0468146	0.725124	Down-regulated in PsA
RP11-467D18.2	0.0468294	1.43961	Up-regulated in PsA
RP11-480G7.2	0.0468294	1.34983	Up-regulated in PsA
SYCP1	0.0468547	0.73088	Down-regulated in PsA
KRT18P7	0.0468575	0.92939	Down-regulated in PsA
RP11-493L12.5	0.0468613	1.06918	Up-regulated in PsA
DEFB106B	0.0469067	0.914115	Down-regulated in PsA

ID2	0.0469126	0.959673	Down-regulated in PsA
DNM1P35	0.0469856	1.14199	Up-regulated in PsA
TGIF2-C20orf24	0.0470179	0.809053	Down-regulated in PsA
MYRFL	0.0470415	0.590101	Down-regulated in PsA
HIF3A	0.0470449	0.888884	Down-regulated in PsA
AP000936.4	0.0470473	1.36269	Up-regulated in PsA
RP11-440J4.2	0.047083	1.41697	Up-regulated in PsA
AIMP1	0.0470861	0.907044	Down-regulated in PsA
CLDN17	0.0471618	0.794788	Down-regulated in PsA
F11-AS1	0.0471633	1.09788	Up-regulated in PsA
IKBKB	0.0471825	0.646689	Down-regulated in PsA
CYP51A1P2	0.0471897	1.3091	Up-regulated in PsA
YME1L1	0.0472061	0.808339	Down-regulated in PsA
RN7SL183P	0.0472188	1.18083	Up-regulated in PsA
RPS3AP43	0.0472205	0.825806	Down-regulated in PsA
VGLL4	0.0472486	0.8191	Down-regulated in PsA
HIP1R	0.0472695	1.13327	Up-regulated in PsA
MKKS	0.0472712	0.536633	Down-regulated in PsA
RP11-884K10.7	0.0472733	0.581556	Down-regulated in PsA
RP11-113E21.3	0.0472911	0.892122	Down-regulated in PsA
BCL2L14	0.0472959	0.880456	Down-regulated in PsA
AC093850.1	0.0473851	1.27894	Up-regulated in PsA
RPL21P28	0.0474009	0.912595	Down-regulated in PsA
EPB41L1	0.047422	0.803128	Down-regulated in PsA
ZCCHC5	0.0474404	1.23144	Up-regulated in PsA

AC008592.5	0.0474458	0.901883	Down-regulated in PsA
MIR6780B	0.0474498	1.1585	Up-regulated in PsA
Y_RNA	0.0474597	1.89194	Up-regulated in PsA
RN7SL815P	0.0474749	1.48592	Up-regulated in PsA
C8orf87	0.0475187	0.727192	Down-regulated in PsA
CTD-2062F14.3	0.0475598	1.31072	Up-regulated in PsA
RN7SL787P	0.0475648	1.11513	Up-regulated in PsA
FLJ46284	0.0476033	0.512586	Down-regulated in PsA
AC006133.3	0.0476133	1.14398	Up-regulated in PsA
PTCD2P2	0.0476352	0.755491	Down-regulated in PsA
RP11-182L7.1	0.0476461	1.58501	Up-regulated in PsA
PLA2G4A	0.0476546	0.661331	Down-regulated in PsA
AC067945.2	0.0476556	0.75111	Down-regulated in PsA
OR9A2	0.0476859	0.833184	Down-regulated in PsA
RP11-85G18.6	0.0477033	1.35653	Up-regulated in PsA
USP16	0.0477433	0.853294	Down-regulated in PsA
NKX2-5	0.047744	0.817954	Down-regulated in PsA
RP11-622C24.1	0.0477919	0.763157	Down-regulated in PsA
EML4	0.0478134	0.795303	Down-regulated in PsA
DYNLT1	0.0478243	0.843825	Down-regulated in PsA
ZNF587P1	0.0478256	0.81302	Down-regulated in PsA
MTND6P33	0.04793	1.23416	Up-regulated in PsA
RP11-567B20.1	0.0479329	1.28966	Up-regulated in PsA
MTCYBP9	0.0479355	0.826529	Down-regulated in PsA
TDRD7	0.0479358	0.751329	Down-regulated in PsA

DTX3	0.0479509	0.745226	Down-regulated in PsA
DGUOK	0.0479893	0.860819	Down-regulated in PsA
FMO6P	0.0480346	1.32902	Up-regulated in PsA
RP11-47A8.5	0.0480358	1.16993	Up-regulated in PsA
bP-21201H5.2	0.0480398	0.401065	Down-regulated in PsA
PRR4	0.0480486	0.582164	Down-regulated in PsA
GLRX5	0.0480625	0.901084	Down-regulated in PsA
MIR6512	0.0480729	1.10941	Up-regulated in PsA
RPL36AP41	0.0481057	0.889649	Down-regulated in PsA
KIAA1456	0.0481605	1.16662	Up-regulated in PsA
PRAMEF9	0.0481676	0.89509	Down-regulated in PsA
MIR23A	0.0481703	1.33884	Up-regulated in PsA
NSUN2	0.0481771	0.77048	Down-regulated in PsA
EDEM2	0.048181	0.88874	Down-regulated in PsA
ZNF775	0.0482074	0.9397	Down-regulated in PsA
CTC-366B18.4	0.0482252	0.662996	Down-regulated in PsA
RP5-1112D6.8	0.0482516	0.486985	Down-regulated in PsA
ABCG8	0.0482626	0.896946	Down-regulated in PsA
DEFB126	0.0482653	0.816187	Down-regulated in PsA
SLC35B3	0.0482809	0.520394	Down-regulated in PsA
BX842568.4	0.0482839	1.30738	Up-regulated in PsA
LINC00488	0.0482933	1.73816	Up-regulated in PsA
DPP3P2	0.048297	0.799491	Down-regulated in PsA
CHIC2	0.0482999	0.703045	Down-regulated in PsA
TCEB1P5	0.0483126	0.782294	Down-regulated in PsA

C6orf203	0.0483285	0.80889	Down-regulated in PsA
UBR3	0.0483313	0.772356	Down-regulated in PsA
DCAF8	0.0483647	0.829344	Down-regulated in PsA
RP11-13M3.1	0.048365	1.73108	Up-regulated in PsA
NUP210	0.0483721	0.808567	Down-regulated in PsA
APC	0.0483943	0.817557	Down-regulated in PsA
NOB1	0.0483946	0.876966	Down-regulated in PsA
P3H3	0.0484023	1.16724	Up-regulated in PsA
ZFAND6	0.0484792	0.81675	Down-regulated in PsA
AGO3	0.0484845	0.751993	Down-regulated in PsA
RP11-182N22.9	0.0484995	1.2939	Up-regulated in PsA
LINC00284	0.048517	0.799284	Down-regulated in PsA
ANAPC1P1	0.0485214	0.697473	Down-regulated in PsA
TRIM32	0.0485413	0.672483	Down-regulated in PsA
HIST1H2BM	0.0485894	1.60167	Up-regulated in PsA
RP11-580I16.2	0.0486138	0.872654	Down-regulated in PsA
C21orf58	0.0486145	0.691364	Down-regulated in PsA
RP11-388E23.2	0.0486523	1.1711	Up-regulated in PsA
RP11-799M12.2	0.0486901	0.424517	Down-regulated in PsA
MIR3130-2	0.0487054	0.812464	Down-regulated in PsA
ADAM24P	0.0487152	1.22391	Up-regulated in PsA
CTC-436P18.4	0.0487166	1.11897	Up-regulated in PsA
LOC100506858	0.0487291	1.11784	Up-regulated in PsA
RFWD2	0.0487315	0.7693	Down-regulated in PsA
GGT7	0.0487393	0.819082	Down-regulated in PsA

RECQL5	0.0487451	0.787332	Down-regulated in PsA
RP11-585K6.2	0.0487696	1.1982	Up-regulated in PsA
ATP2B4	0.0487986	0.829928	Down-regulated in PsA
RP11-27M5.1	0.0488695	0.859464	Down-regulated in PsA
RANGAP1	0.0488697	0.912863	Down-regulated in PsA
ACLY	0.0488781	0.846947	Down-regulated in PsA
GPR143	0.0489021	1.06384	Up-regulated in PsA
LL22NC03-30E12.13	0.0489092	0.920143	Down-regulated in PsA
GPC2	0.0489365	0.780743	Down-regulated in PsA
RP11-510M2.1	0.04894	1.30249	Up-regulated in PsA
RP11-400N13.1	0.0489501	1.31173	Up-regulated in PsA
RP11-94D20.1	0.0489707	1.21928	Up-regulated in PsA
RP11-72M10.4	0.0490007	1.18392	Up-regulated in PsA
RP11-75D3.1	0.0490092	0.797983	Down-regulated in PsA
RPL7A	0.049028	1.02943	Up-regulated in PsA
IL20RB-AS1	0.0490495	1.2024	Up-regulated in PsA
BMP15	0.0490515	0.809734	Down-regulated in PsA
TRAPPC9	0.0490627	1.2215	Up-regulated in PsA
RP11-298O21.2	0.0490821	0.728398	Down-regulated in PsA
RP11-848D3.4	0.0491019	1.24315	Up-regulated in PsA
AC012593.1	0.0491159	0.657887	Down-regulated in PsA
UTP20	0.0491199	0.595066	Down-regulated in PsA
TRAM2	0.0491283	0.795822	Down-regulated in PsA
RP4-635A23.4	0.0491592	0.76447	Down-regulated in PsA
RP11-686F15.2	0.0491811	0.746976	Down-regulated in PsA

RNU6-101P	0.0491852	1.0603 Up-regulated in PsA
RP11-98D18.17	0.0491921	1.1035 Up-regulated in PsA
RP11-226P1.1	0.0491997	0.665215 Down-regulated in PsA
ENKUR	0.0492017	1.40405 Up-regulated in PsA
KLF13	0.0492147	0.710764 Down-regulated in PsA
CHRND	0.0492755	0.790776 Down-regulated in PsA
LOC284788	0.049277	1.24844 Up-regulated in PsA
RP11-1000B6.2	0.0492781	1.24727 Up-regulated in PsA
RP11-386M24.8	0.0492862	0.92627 Down-regulated in PsA
RP11-555G19.2	0.0492965	0.661768 Down-regulated in PsA
ANKRD27	0.0493031	0.668384 Down-regulated in PsA
SCARNA23	0.0493115	1.91568 Up-regulated in PsA
ATG4A	0.0493226	0.705562 Down-regulated in PsA
P2RX2	0.0493339	0.818287 Down-regulated in PsA
KDM3B	0.0493652	0.815604 Down-regulated in PsA
PEX6	0.0493675	0.845834 Down-regulated in PsA
AP000962.1	0.0493726	0.636498 Down-regulated in PsA
SMPD3	0.0493779	1.36935 Up-regulated in PsA
THEM5	0.0493835	1.20234 Up-regulated in PsA
LINC01080	0.0493919	0.689799 Down-regulated in PsA
DEFB123	0.0494022	0.791234 Down-regulated in PsA
C2orf88	0.0494174	1.32627 Up-regulated in PsA
RP11-240E2.2	0.049424	0.732995 Down-regulated in PsA
RP11-295G20.2	0.0494321	0.814687 Down-regulated in PsA
RP11-115A15.4	0.0494605	1.34107 Up-regulated in PsA

PKNOX2-AS1	0.0494718	0.843678	Down-regulated in PsA
LUC7L	0.0494759	0.829749	Down-regulated in PsA
MIR6829	0.049495	1.20317	Up-regulated in PsA
AC007365.4	0.0494964	1.30377	Up-regulated in PsA
WSCD2	0.0495189	0.664841	Down-regulated in PsA
ZNF546	0.0495353	0.903616	Down-regulated in PsA
AC074117.13	0.0495549	0.879415	Down-regulated in PsA
HMGN2P21	0.0495912	0.793912	Down-regulated in PsA
CTD-2534J5.1	0.0496075	0.802192	Down-regulated in PsA
AC005943.6	0.0496353	1.26054	Up-regulated in PsA
MIR302C	0.0496642	0.804858	Down-regulated in PsA
FGF22	0.0496875	0.840509	Down-regulated in PsA
LOC339059	0.0497279	1.14757	Up-regulated in PsA
SNORD4A	0.0497368	1.30849	Up-regulated in PsA
RP11-548K12.2	0.0497571	1.32019	Up-regulated in PsA
RP11-148O21.4	0.0497598	0.706894	Down-regulated in PsA
CTD-2282P23.1	0.0497667	1.3466	Up-regulated in PsA
RNF138P2	0.0497668	1.10276	Up-regulated in PsA
ZCWPW2	0.0497748	0.898568	Down-regulated in PsA
RP11-335O13.8	0.0497798	1.20831	Up-regulated in PsA
LOC105369785	0.0497902	1.31082	Up-regulated in PsA
RP11-62H7.3	0.0498165	0.830792	Down-regulated in PsA
MSX2	0.0498247	0.82666	Down-regulated in PsA
AMD1P2	0.049828	1.20477	Up-regulated in PsA
AMD1P2	0.049828	1.20477	Up-regulated in PsA

NIPBL	0.0498919	0.68871	Down-regulated in PsA
FCRL6	0.0499101	1.19096	Up-regulated in PsA
E2F3P1	0.0499363	1.07991	Up-regulated in PsA
LCMT1-AS1	0.0499427	0.855509	Down-regulated in PsA
SRIP3	0.049943	0.911923	Down-regulated in PsA
RUNDC3A	0.049977	0.86613	Down-regulated in PsA
CTC-281M20.3	0.0499888	1.16983	Up-regulated in PsA
FAM66B	0.0499955	1.19834	Up-regulated in PsA

Supplementary Table S3. The output result of pathway enrichment analysis by Partek Genomics Suite 7.0 based on the up-regultaed genes in PsA samples. Enrichment -p-value was calculated based on hyper-geometric distribution.

Pathway Name	Database	Enrichment Score	Enrichment p-value	Pathway ID
Cytokine-cytokine receptor interaction	kegg	4.94864	0.00709306	kegg_pathway_27
PPAR signaling pathway	kegg	4.03418	0.0177002	kegg_pathway_13
Phenylalanine, tyrosine and tryptophan biosynthesis	kegg	2.7063	0.0667835	kegg_pathway_271
Olfactory transduction	kegg	2.50101	0.0820019	kegg_pathway_100
Synaptic vesicle cycle	kegg	2.36201	0.0942306	kegg_pathway_91
Taste transduction	kegg	2.27745	0.102545	kegg_pathway_101
Nicotine addiction	kegg	2.26308	0.10403	kegg_pathway_148
Chemokine signaling pathway	kegg	2.1217	0.119828	kegg_pathway_28
Protein digestion and absorption	kegg	2.07102	0.126057	kegg_pathway_135
Basal transcription factors	kegg	2.06781	0.126463	kegg_pathway_8
Cysteine and methionine metabolism	kegg	2.03196	0.131079	kegg_pathway_262
Sphingolipid metabolism	kegg	1.99706	0.135734	kegg_pathway_299
Ubiquinone and other terpenoid-quinone biosynthesis	kegg	1.95819	0.141114	kegg_pathway_253
Regulation of lipolysis in adipocytes	kegg	1.74786	0.174147	kegg_pathway_118
Insulin resistance	kegg	1.68802	0.184886	kegg_pathway_122
Neuroactive ligand-receptor interaction	kegg	1.68478	0.185485	kegg_pathway_34
Glycosphingolipid biosynthesis - ganglio series	kegg	1.67463	0.187377	kegg_pathway_302
Serotonergic synapse	kegg	1.61526	0.198839	kegg_pathway_96
Phenylalanine metabolism	kegg	1.56267	0.209575	kegg_pathway_269
Primary bile acid biosynthesis	kegg	1.56267	0.209575	kegg_pathway_252

Mitophagy - animal	kegg	1.49536	0.224167	kegg_pathway_223
Alcoholism	kegg	1.45252	0.233979	kegg_pathway_149
Axon guidance	kegg	1.43995	0.23694	kegg_pathway_59
Adipocytokine signaling pathway	kegg	1.40851	0.244508	kegg_pathway_115
Relaxin signaling pathway	kegg	1.33093	0.264232	kegg_pathway_55
Biosynthesis of amino acids	kegg	1.30921	0.270033	kegg_pathway_330
Mannose type O-glycan biosynthesis	kegg	1.29966	0.272625	kegg_pathway_284
Histidine metabolism	kegg	1.29966	0.272625	kegg_pathway_267
Proximal tubule bicarbonate reclamation	kegg	1.29966	0.272625	kegg_pathway_129
ECM-receptor interaction	kegg	1.16859	0.310805	kegg_pathway_64
Fatty acid elongation	kegg	1.16519	0.311864	kegg_pathway_248
Peroxisome	kegg	1.1524	0.315877	kegg_pathway_46
Retrograde endocannabinoid signaling	kegg	1.10296	0.331887	kegg_pathway_93
cAMP signaling pathway	kegg	1.10243	0.332063	kegg_pathway_26
Regulation of actin cytoskeleton	kegg	1.09304	0.335196	kegg_pathway_104
Non-alcoholic fatty liver disease (NAFLD)	kegg	1.09165	0.335663	kegg_pathway_123
Pentose phosphate pathway	kegg	1.07907	0.33991	kegg_pathway_242
Ribosome	kegg	1.06941	0.343212	kegg_pathway_3
GABAergic synapse	kegg	1.06098	0.346117	kegg_pathway_97
RNA polymerase	kegg	1.05267	0.349005	kegg_pathway_7
beta-Alanine metabolism	kegg	1.05267	0.349005	kegg_pathway_272
Morphine addiction	kegg	1.03252	0.356107	kegg_pathway_147
Autophagy - other	kegg	1.02729	0.357976	kegg_pathway_224
PI3K-Akt signaling pathway	kegg	1.02609	0.358404	kegg_pathway_48
Small cell lung cancer	kegg	1.005	0.366044	kegg_pathway_191

Apoptosis - multiple species	kegg	1.00287	0.366824	kegg_pathway_211
Fructose and mannose metabolism	kegg	1.00287	0.366824	kegg_pathway_244
Base excision repair	kegg	0.979361	0.375551	kegg_pathway_14
SNARE interactions in vesicular transport	kegg	0.979361	0.375551	kegg_pathway_40
Endocrine resistance	kegg	0.978371	0.375923	kegg_pathway_215
Amoebiasis	kegg	0.965379	0.380839	kegg_pathway_163
Circadian entrainment	kegg	0.952592	0.38574	kegg_pathway_89
Tyrosine metabolism	kegg	0.934837	0.39265	kegg_pathway_268
Starch and sucrose metabolism	kegg	0.934837	0.39265	kegg_pathway_279
DNA replication	kegg	0.934837	0.39265	kegg_pathway_9
HIF-1 signaling pathway	kegg	0.915439	0.400341	kegg_pathway_30
AGE-RAGE signaling pathway in diabetic complications	kegg	0.915439	0.400341	kegg_pathway_208
Primary immunodeficiency	kegg	0.913734	0.401024	kegg_pathway_202
NF-kappa B signaling pathway	kegg	0.903441	0.405173	kegg_pathway_29
Glucagon signaling pathway	kegg	0.891632	0.409986	kegg_pathway_117
Ferroptosis	kegg	0.854566	0.425468	kegg_pathway_225
Endocytosis	kegg	0.846186	0.429048	kegg_pathway_44
Pathways in cancer	kegg	0.812625	0.443692	kegg_pathway_173
Fatty acid degradation	kegg	0.784105	0.456528	kegg_pathway_249
Cholinergic synapse	kegg	0.782997	0.457034	kegg_pathway_95
Hedgehog signaling pathway	kegg	0.751989	0.471428	kegg_pathway_57
Intestinal immune network for IgA production	kegg	0.751989	0.471428	kegg_pathway_87
Notch signaling pathway	kegg	0.721703	0.485924	kegg_pathway_56
Ovarian steroidogenesis	kegg	0.707199	0.493023	kegg_pathway_108

Huntington disease	kegg	0.699397	0.496885	kegg_pathway_143
Arginine and proline metabolism	kegg	0.693095	0.500026	kegg_pathway_266
Cholesterol metabolism	kegg	0.693095	0.500026	kegg_pathway_232
Mineral absorption	kegg	0.679378	0.506932	kegg_pathway_139
Osteoclast differentiation	kegg	0.655537	0.519163	kegg_pathway_61
Autophagy - animal	kegg	0.63932	0.527651	kegg_pathway_41
FoxO signaling pathway	kegg	0.615823	0.540196	kegg_pathway_31
Systemic lupus erythematosus	kegg	0.615823	0.540196	kegg_pathway_198
Rap1 signaling pathway	kegg	0.614356	0.540989	kegg_pathway_23
Lysine degradation	kegg	0.58181	0.558886	kegg_pathway_265
Ubiquitin mediated proteolysis	kegg	0.571641	0.564598	kegg_pathway_38
Apelin signaling pathway	kegg	0.571641	0.564598	kegg_pathway_222
Human T-cell leukemia virus 1 infection	kegg	0.545232	0.579707	kegg_pathway_170
Basal cell carcinoma	kegg	0.539927	0.582791	kegg_pathway_186
Arachidonic acid metabolism	kegg	0.539927	0.582791	kegg_pathway_296
Shigellosis	kegg	0.53007	0.588564	kegg_pathway_154
MicroRNAs in cancer	kegg	0.524122	0.592075	kegg_pathway_178
Central carbon metabolism in cancer	kegg	0.520443	0.594257	kegg_pathway_193
Retinol metabolism	kegg	0.501846	0.605412	kegg_pathway_316
Phospholipase D signaling pathway	kegg	0.499368	0.606914	kegg_pathway_207
Gastric cancer	kegg	0.493307	0.610604	kegg_pathway_231
Drug metabolism - cytochrome P450	kegg	0.492865	0.610874	kegg_pathway_323
Glycolysis / Gluconeogenesis	kegg	0.492865	0.610874	kegg_pathway_240
Renal cell carcinoma	kegg	0.492865	0.610874	kegg_pathway_180
RIG-I-like receptor signaling pathway	kegg	0.475497	0.621576	kegg_pathway_76

B cell receptor signaling pathway	kegg	0.467101	0.626817	kegg_pathway_82
p53 signaling pathway	kegg	0.458888	0.631986	kegg_pathway_37
Metabolism of xenobiotics by cytochrome P450	kegg	0.458888	0.631986	kegg_pathway_322
Adherens junction	kegg	0.458888	0.631986	kegg_pathway_66
Bile secretion	kegg	0.458888	0.631986	kegg_pathway_137
Bacterial invasion of epithelial cells	kegg	0.450854	0.637084	kegg_pathway_150
Wnt signaling pathway	kegg	0.447552	0.639191	kegg_pathway_54
Necroptosis	kegg	0.421264	0.656217	kegg_pathway_226
Complement and coagulation cascades	kegg	0.406111	0.666236	kegg_pathway_70
Chemical carcinogenesis	kegg	0.392409	0.675428	kegg_pathway_176
Alzheimer disease	kegg	0.377961	0.685257	kegg_pathway_140
Salmonella infection	kegg	0.360504	0.697325	kegg_pathway_155
Insulin secretion	kegg	0.360504	0.697325	kegg_pathway_106
Fc gamma R-mediated phagocytosis	kegg	0.331623	0.717758	kegg_pathway_84
mRNA surveillance pathway	kegg	0.331623	0.717758	kegg_pathway_5
Kaposi sarcoma-associated herpesvirus infection	kegg	0.315794	0.72921	kegg_pathway_227
Calcium signaling pathway	kegg	0.308341	0.734665	kegg_pathway_24
Glycerophospholipid metabolism	kegg	0.300455	0.740481	kegg_pathway_294
Progesterone-mediated oocyte maturation	kegg	0.295596	0.744088	kegg_pathway_109
Choline metabolism in cancer	kegg	0.290827	0.747645	kegg_pathway_194
Inflammatory mediator regulation of TRP channels	kegg	0.286146	0.751153	kegg_pathway_103
Ribosome biogenesis in eukaryotes	kegg	0.286146	0.751153	kegg_pathway_2
Focal adhesion	kegg	0.273688	0.760569	kegg_pathway_63
C-type lectin receptor signaling pathway	kegg	0.26826	0.764709	kegg_pathway_236
Carbon metabolism	kegg	0.232445	0.792593	kegg_pathway_327

Glutamatergic synapse	kegg	0.228808	0.795481	kegg_pathway_94
Thyroid hormone signaling pathway	kegg	0.221721	0.801139	kegg_pathway_114
Sphingolipid signaling pathway	kegg	0.21154	0.809337	kegg_pathway_33
Platelet activation	kegg	0.195696	0.822262	kegg_pathway_71
Cell cycle	kegg	0.195696	0.822262	kegg_pathway_35
Oocyte meiosis	kegg	0.192686	0.824741	kegg_pathway_36
Ras signaling pathway	kegg	0.184891	0.831195	kegg_pathway_22
Thermogenesis	kegg	0.184891	0.831195	kegg_pathway_234
Dopaminergic synapse	kegg	0.175651	0.838911	kegg_pathway_98
Human papillomavirus infection	kegg	0.175419	0.839105	kegg_pathway_229
Vascular smooth muscle contraction	kegg	0.172973	0.84116	kegg_pathway_53
Oxidative phosphorylation	kegg	0.167751	0.845564	kegg_pathway_255
Apoptosis	kegg	0.162698	0.849848	kegg_pathway_50
Insulin signaling pathway	kegg	0.160233	0.851945	kegg_pathway_105
Signaling pathways regulating pluripotency of stem cells	kegg	0.155423	0.856053	kegg_pathway_69
Parkinson disease	kegg	0.148494	0.862005	kegg_pathway_141
Cell adhesion molecules (CAMs)	kegg	0.146259	0.863934	kegg_pathway_65
Cushing syndrome	kegg	0.122054	0.885101	kegg_pathway_235
JAK-STAT signaling pathway	kegg	0.108294	0.897364	kegg_pathway_78
Protein processing in endoplasmic reticulum	kegg	0.106691	0.898803	kegg_pathway_42
cGMP-PKG signaling pathway	kegg	0.103561	0.901621	kegg_pathway_25
Influenza A	kegg	0.103561	0.901621	kegg_pathway_169
RNA transport	kegg	0.103561	0.901621	kegg_pathway_4
Hepatocellular carcinoma	kegg	0.102032	0.903001	kegg_pathway_230

Proteoglycans in cancer	kegg	0.0618158	0.940056	kegg_pathway_177
Viral carcinogenesis	kegg	0.0618158	0.940056	kegg_pathway_175
Human immunodeficiency virus 1 infection	kegg	0.0541975	0.947245	kegg_pathway_239
Human cytomegalovirus infection	kegg	0.043557	0.957378	kegg_pathway_238
Herpes simplex virus 1 infection	kegg	0.0321142	0.968396	kegg_pathway_171
Metabolic pathways	kegg	0.0163164	0.983816	kegg_pathway_326
MAPK signaling pathway	kegg	0.015789	0.984335	kegg_pathway_20

Supplementary Table S4. The output result of pathway enrichment analysis by Partek Genomics Suite 7.0 based on the down-regultaed genes in PsA samples. Enrichment -p-value was calculated based on hyper-geometric distribution.

Pathway Name	Database	Enrichment Score	Enrichment p-value	Pathway ID
Homologous recombination	kegg	6.93223	0.000975825	kegg_pathway_17
Protein processing in endoplasmic reticulum	kegg	6.29289	0.00184941	kegg_pathway_42
Apoptosis	kegg	4.60809	0.00997089	kegg_pathway_50
Fanconi anemia pathway	kegg	4.11426	0.0163381	kegg_pathway_19
Salivary secretion	kegg	4.08869	0.0167611	kegg_pathway_131
Tryptophan metabolism	kegg	3.93999	0.0194485	kegg_pathway_270
RNA degradation	kegg	3.93519	0.0195419	kegg_pathway_6
Ubiquitin mediated proteolysis	kegg	3.7425	0.0236948	kegg_pathway_38
Epithelial cell signaling in Helicobacter pylori infection	kegg	3.70887	0.0245052	kegg_pathway_152
Apoptosis - multiple species	kegg	3.67429	0.0253673	kegg_pathway_211
Peroxisome	kegg	3.64543	0.0261101	kegg_pathway_46
RIG-I-like receptor signaling pathway	kegg	3.63157	0.0264746	kegg_pathway_76
Basal transcription factors	kegg	3.62825	0.0265625	kegg_pathway_8
Type II diabetes mellitus	kegg	3.53113	0.0292717	kegg_pathway_121
Nucleotide excision repair	kegg	3.43715	0.0321563	kegg_pathway_15
Longevity regulating pathway	kegg	3.25252	0.0386768	kegg_pathway_210
NOD-like receptor signaling pathway	kegg	3.25014	0.0387687	kegg_pathway_75
Hepatitis B	kegg	3.15543	0.04262	kegg_pathway_167
Shigellosis	kegg	3.09963	0.0450657	kegg_pathway_154
Synaptic vesicle cycle	kegg	3.00979	0.0493022	kegg_pathway_91

TNF signaling pathway	kegg	2.88123	0.0560655 kegg_pathway_85
Fc epsilon RI signaling pathway	kegg	2.82667	0.0592098 kegg_pathway_83
Legionellosis	kegg	2.78261	0.061877 kegg_pathway_157
ErbB signaling pathway	kegg	2.71416	0.0662605 kegg_pathway_21
Fatty acid degradation	kegg	2.60779	0.0736976 kegg_pathway_249
Platinum drug resistance	kegg	2.57953	0.0758099 kegg_pathway_214
One carbon pool by folate	kegg	2.52002	0.0804581 kegg_pathway_307
Spliceosome	kegg	2.4805	0.083701 kegg_pathway_10
RNA transport	kegg	2.46055	0.0853878 kegg_pathway_4
AMPK signaling pathway	kegg	2.41828	0.0890746 kegg_pathway_49
Chronic myeloid leukemia	kegg	2.35517	0.0948774 kegg_pathway_189
Fc gamma R-mediated phagocytosis	kegg	2.34966	0.0954013 kegg_pathway_84
Notch signaling pathway	kegg	2.31549	0.0987175 kegg_pathway_56
Amino sugar and nucleotide sugar metabolism	kegg	2.31549	0.0987175 kegg_pathway_285
Terpenoid backbone biosynthesis	kegg	2.29354	0.100909 kegg_pathway_318
Insulin resistance	kegg	2.27068	0.103242 kegg_pathway_122
IL-17 signaling pathway	kegg	2.25491	0.104883 kegg_pathway_220
Ubiquinone and other terpenoid-quinone biosynthesis	kegg	2.23678	0.106802 kegg_pathway_253
Cellular senescence	kegg	2.1777	0.113302 kegg_pathway_228
Osteoclast differentiation	kegg	2.17575	0.113523 kegg_pathway_61
Toxoplasmosis	kegg	2.14512	0.117054 kegg_pathway_162
alpha-Linolenic acid metabolism	kegg	2.00294	0.134938 kegg_pathway_298
Breast cancer	kegg	2.00022	0.135306 kegg_pathway_216
Phosphatidylinositol signaling system	kegg	1.99282	0.136311 kegg_pathway_32
Biotin metabolism	kegg	1.92055	0.146527 kegg_pathway_313

B cell receptor signaling pathway	kegg	1.85071	0.157126	kegg_pathway_82
Sphingolipid signaling pathway	kegg	1.84198	0.158504	kegg_pathway_33
Glycosaminoglycan biosynthesis - keratan sulfate	kegg	1.8327	0.159981	kegg_pathway_289
Hippo signaling pathway	kegg	1.80878	0.163854	kegg_pathway_62
Toll-like receptor signaling pathway	kegg	1.76059	0.171943	kegg_pathway_74
Collecting duct acid secretion	kegg	1.75865	0.172277	kegg_pathway_130
Metabolic pathways	kegg	1.75538	0.172842	kegg_pathway_326
Porphyrin and chlorophyll metabolism	kegg	1.72437	0.178286	kegg_pathway_317
Inositol phosphate metabolism	kegg	1.71628	0.179734	kegg_pathway_292
Th1 and Th2 cell differentiation	kegg	1.71571	0.179836	kegg_pathway_218
Herpes simplex virus 1 infection	kegg	1.71163	0.180571	kegg_pathway_171
VEGF signaling pathway	kegg	1.6846	0.185518	kegg_pathway_60
Pancreatic cancer	kegg	1.67373	0.187547	kegg_pathway_181
Nicotinate and nicotinamide metabolism	kegg	1.61642	0.198608	kegg_pathway_311
Cysteine and methionine metabolism	kegg	1.5596	0.21022	kegg_pathway_262
Antifolate resistance	kegg	1.55062	0.212116	kegg_pathway_217
Longevity regulating pathway - multiple species	kegg	1.54682	0.212923	kegg_pathway_209
Endocrine resistance	kegg	1.53205	0.216093	kegg_pathway_215
Selenocompound metabolism	kegg	1.52888	0.216778	kegg_pathway_275
Autophagy - other	kegg	1.48803	0.225817	kegg_pathway_224
Dopaminergic synapse	kegg	1.46183	0.231811	kegg_pathway_98
Phenylalanine, tyrosine and tryptophan biosynthesis	kegg	1.46056	0.232106	kegg_pathway_271
2-Oxocarboxylic acid metabolism	kegg	1.44343	0.236117	kegg_pathway_328
Fructose and mannose metabolism	kegg	1.42845	0.239681	kegg_pathway_244
Ovarian steroidogenesis	kegg	1.41217	0.243613	kegg_pathway_108

Pathways in cancer	kegg	1.3864	0.249973	kegg_pathway_173
HIF-1 signaling pathway	kegg	1.36754	0.254734	kegg_pathway_30
Inflammatory mediator regulation of TRP channels	kegg	1.36754	0.254734	kegg_pathway_103
Glycosaminoglycan degradation	kegg	1.36436	0.255544	kegg_pathway_287
MAPK signaling pathway	kegg	1.35261	0.258564	kegg_pathway_20
NF-kappa B signaling pathway	kegg	1.33673	0.262702	kegg_pathway_29
Vibrio cholerae infection	kegg	1.32239	0.266497	kegg_pathway_151
Neurotrophin signaling pathway	kegg	1.31501	0.268472	kegg_pathway_92
Vitamin B6 metabolism	kegg	1.30332	0.271629	kegg_pathway_310
Phosphonate and phosphinate metabolism	kegg	1.30332	0.271629	kegg_pathway_274
MicroRNAs in cancer	kegg	1.28294	0.277222	kegg_pathway_178
Measles	kegg	1.275	0.279432	kegg_pathway_168
Fluid shear stress and atherosclerosis	kegg	1.275	0.279432	kegg_pathway_221
Colorectal cancer	kegg	1.27008	0.280809	kegg_pathway_179
Adipocytokine signaling pathway	kegg	1.2691	0.281084	kegg_pathway_115
Pyruvate metabolism	kegg	1.26584	0.282001	kegg_pathway_303
Wnt signaling pathway	kegg	1.24345	0.288388	kegg_pathway_54
Fatty acid metabolism	kegg	1.23874	0.28975	kegg_pathway_329
Aminoacyl-tRNA biosynthesis	kegg	1.19904	0.301483	kegg_pathway_321
p53 signaling pathway	kegg	1.16622	0.311542	kegg_pathway_37
Pathogenic Escherichia coli infection	kegg	1.1607	0.313268	kegg_pathway_153
Glutathione metabolism	kegg	1.1607	0.313268	kegg_pathway_278
Other types of O-glycan biosynthesis	kegg	1.15907	0.313777	kegg_pathway_283
mRNA surveillance pathway	kegg	1.11994	0.326298	kegg_pathway_5
Epstein-Barr virus infection	kegg	1.09998	0.332879	kegg_pathway_172

Mismatch repair	kegg	1.09958	0.33301	kegg_pathway_16
Histidine metabolism	kegg	1.09958	0.33301	kegg_pathway_267
Autophagy - animal	kegg	1.08683	0.337284	kegg_pathway_41
Human papillomavirus infection	kegg	1.07044	0.342859	kegg_pathway_229
Small cell lung cancer	kegg	1.06483	0.344786	kegg_pathway_191
Bladder cancer	kegg	1.03976	0.35354	kegg_pathway_188
Fat digestion and absorption	kegg	1.03976	0.35354	kegg_pathway_136
Hepatocellular carcinoma	kegg	1.03116	0.356593	kegg_pathway_230
Non-alcoholic fatty liver disease (NAFLD)	kegg	1.02484	0.358855	kegg_pathway_123
Lysine degradation	kegg	1.01972	0.360697	kegg_pathway_265
Kaposi sarcoma-associated herpesvirus infection	kegg	1.01952	0.360769	kegg_pathway_227
FoxO signaling pathway	kegg	1.01927	0.360859	kegg_pathway_31
Endocytosis	kegg	0.995361	0.36959	kegg_pathway_44
Calcium signaling pathway	kegg	0.983422	0.374029	kegg_pathway_24
Sulfur metabolism	kegg	0.971719	0.378432	kegg_pathway_320
Long-term depression	kegg	0.956014	0.384422	kegg_pathway_99
Glycosylphosphatidylinositol (GPI)-anchor biosynthesis	kegg	0.942568	0.389626	kegg_pathway_293
Pancreatic secretion	kegg	0.938196	0.391333	kegg_pathway_133
Choline metabolism in cancer	kegg	0.914655	0.400655	kegg_pathway_194
Hepatitis C	kegg	0.907861	0.403386	kegg_pathway_166
Biosynthesis of unsaturated fatty acids	kegg	0.896402	0.408035	kegg_pathway_325
Arachidonic acid metabolism	kegg	0.89639	0.40804	kegg_pathway_296
Cytosolic DNA-sensing pathway	kegg	0.89639	0.40804	kegg_pathway_77
Ribosome biogenesis in eukaryotes	kegg	0.891674	0.409969	kegg_pathway_2
ABC transporters	kegg	0.891057	0.410222	kegg_pathway_1

Synthesis and degradation of ketone bodies	kegg	0.890523	0.410441	kegg_pathway_250
Chagas disease (American trypanosomiasis)	kegg	0.825961	0.437814	kegg_pathway_159
Ether lipid metabolism	kegg	0.825504	0.438014	kegg_pathway_295
Taurine and hypotaurine metabolism	kegg	0.819153	0.440805	kegg_pathway_273
Non-small cell lung cancer	kegg	0.813958	0.443101	kegg_pathway_192
Acute myeloid leukemia	kegg	0.813958	0.443101	kegg_pathway_190
C-type lectin receptor signaling pathway	kegg	0.805092	0.447047	kegg_pathway_236
Valine, leucine and isoleucine degradation	kegg	0.794682	0.451725	kegg_pathway_263
Necroptosis	kegg	0.786546	0.455415	kegg_pathway_226
Salmonella infection	kegg	0.785574	0.455858	kegg_pathway_155
Cell cycle	kegg	0.784824	0.4562	kegg_pathway_35
Circadian rhythm	kegg	0.773187	0.46154	kegg_pathway_88
Citrate cycle (TCA cycle)	kegg	0.773187	0.46154	kegg_pathway_241
Glyoxylate and dicarboxylate metabolism	kegg	0.773187	0.46154	kegg_pathway_304
Cocaine addiction	kegg	0.765077	0.465298	kegg_pathway_145
Th17 cell differentiation	kegg	0.764828	0.465414	kegg_pathway_219
Adrenergic signaling in cardiomyocytes	kegg	0.751645	0.47159	kegg_pathway_52
Gap junction	kegg	0.742228	0.476052	kegg_pathway_68
Arginine and proline metabolism	kegg	0.736637	0.478721	kegg_pathway_266
N-Glycan biosynthesis	kegg	0.736637	0.478721	kegg_pathway_280
Cholesterol metabolism	kegg	0.736637	0.478721	kegg_pathway_232
RNA polymerase	kegg	0.736593	0.478742	kegg_pathway_7
Mucin type O-glycan biosynthesis	kegg	0.736593	0.478742	kegg_pathway_282
GABAergic synapse	kegg	0.721427	0.486058	kegg_pathway_97
Human cytomegalovirus infection	kegg	0.71022	0.491536	kegg_pathway_238

Endocrine and other factor-regulated calcium reabsorption	kegg	0.709307	0.491985	kegg_pathway_127
Antigen processing and presentation	kegg	0.693141	0.500003	kegg_pathway_72
Gastric cancer	kegg	0.687833	0.502664	kegg_pathway_231
Morphine addiction	kegg	0.681491	0.505862	kegg_pathway_147
Leishmaniasis	kegg	0.671212	0.511089	kegg_pathway_158
Tight junction	kegg	0.665686	0.513921	kegg_pathway_67
TGF-beta signaling pathway	kegg	0.662325	0.515651	kegg_pathway_58
Human immunodeficiency virus 1 infection	kegg	0.653757	0.520088	kegg_pathway_239
PPAR signaling pathway	kegg	0.649967	0.522063	kegg_pathway_13
GnRH signaling pathway	kegg	0.643672	0.52536	kegg_pathway_107
mTOR signaling pathway	kegg	0.643207	0.525604	kegg_pathway_47
Thermogenesis	kegg	0.636756	0.529006	kegg_pathway_234
Regulation of actin cytoskeleton	kegg	0.617244	0.539429	kegg_pathway_104
Thyroid hormone signaling pathway	kegg	0.590192	0.554221	kegg_pathway_114
Tyrosine metabolism	kegg	0.580771	0.559467	kegg_pathway_268
Estrogen signaling pathway	kegg	0.57503	0.562688	kegg_pathway_110
Insulin signaling pathway	kegg	0.57503	0.562688	kegg_pathway_105
Glycerophospholipid metabolism	kegg	0.573938	0.563303	kegg_pathway_294
Pertussis	kegg	0.571411	0.564728	kegg_pathway_156
Tuberculosis	kegg	0.561524	0.570339	kegg_pathway_165
Human T-cell leukemia virus 1 infection	kegg	0.560293	0.571042	kegg_pathway_170
Aldosterone-regulated sodium reabsorption	kegg	0.554243	0.574507	kegg_pathway_126
African trypanosomiasis	kegg	0.554243	0.574507	kegg_pathway_160
Thyroid cancer	kegg	0.554243	0.574507	kegg_pathway_185
Signaling pathways regulating pluripotency of stem cells	kegg	0.547726	0.578263	kegg_pathway_69

Endometrial cancer	kegg	0.545198	0.579727 kegg_pathway_182
EGFR tyrosine kinase inhibitor resistance	kegg	0.535696	0.585262 kegg_pathway_213
AGE-RAGE signaling pathway in diabetic complications	kegg	0.526396	0.59073 kegg_pathway_208
T cell receptor signaling pathway	kegg	0.526396	0.59073 kegg_pathway_81
Nitrogen metabolism	kegg	0.522753	0.592886 kegg_pathway_319
Fatty acid biosynthesis	kegg	0.522753	0.592886 kegg_pathway_247
Phenylalanine metabolism	kegg	0.522753	0.592886 kegg_pathway_269
Lysosome	kegg	0.490775	0.612152 kegg_pathway_43
Taste transduction	kegg	0.486156	0.614986 kegg_pathway_101
Nicotine addiction	kegg	0.482301	0.617361 kegg_pathway_148
Transcriptional misregulation in cancer	kegg	0.471985	0.623763 kegg_pathway_174
Basal cell carcinoma	kegg	0.452181	0.636239 kegg_pathway_186
Retrograde endocannabinoid signaling	kegg	0.438781	0.644822 kegg_pathway_93
Inflammatory bowel disease (IBD)	kegg	0.435588	0.646884 kegg_pathway_197
Ras signaling pathway	kegg	0.432913	0.648617 kegg_pathway_22
Glycosaminoglycan biosynthesis - chondroitin sulfate / dermatan sulfate	kegg	0.426696	0.652662 kegg_pathway_288
Purine metabolism	kegg	0.418067	0.658318 kegg_pathway_257
Arginine biosynthesis	kegg	0.399621	0.670574 kegg_pathway_256
Retinol metabolism	kegg	0.389368	0.677485 kegg_pathway_316
Glycolysis / Gluconeogenesis	kegg	0.375072	0.68724 kegg_pathway_240
Amphetamine addiction	kegg	0.375072	0.68724 kegg_pathway_146
Rheumatoid arthritis	kegg	0.374689	0.687503 kegg_pathway_199
Leukocyte transendothelial migration	kegg	0.370749	0.690217 kegg_pathway_86
Proteasome	kegg	0.366766	0.692972 kegg_pathway_11

Hedgehog signaling pathway	kegg	0.366766	0.692972	kegg_pathway_57
Oxidative phosphorylation	kegg	0.365161	0.694085	kegg_pathway_255
Carbon metabolism	kegg	0.359943	0.697716	kegg_pathway_327
Proximal tubule bicarbonate reclamation	kegg	0.35143	0.703681	kegg_pathway_129
Mannose type O-glycan biosynthesis	kegg	0.35143	0.703681	kegg_pathway_284
Sphingolipid metabolism	kegg	0.350547	0.704303	kegg_pathway_299
Glutamatergic synapse	kegg	0.34943	0.70509	kegg_pathway_94
Prolactin signaling pathway	kegg	0.34802	0.706085	kegg_pathway_112
Axon guidance	kegg	0.327482	0.720736	kegg_pathway_59
Adherens junction	kegg	0.322895	0.72405	kegg_pathway_66
Melanoma	kegg	0.322895	0.72405	kegg_pathway_187
Metabolism of xenobiotics by cytochrome P450	kegg	0.322895	0.72405	kegg_pathway_322
Rap1 signaling pathway	kegg	0.30065	0.740337	kegg_pathway_23
Thyroid hormone synthesis	kegg	0.299555	0.741148	kegg_pathway_113
Biosynthesis of amino acids	kegg	0.299555	0.741148	kegg_pathway_330
Amyotrophic lateral sclerosis (ALS)	kegg	0.292772	0.746192	kegg_pathway_142
Maturity onset diabetes of the young	kegg	0.291394	0.747221	kegg_pathway_125
Folate biosynthesis	kegg	0.291394	0.747221	kegg_pathway_315
Renin-angiotensin system	kegg	0.291394	0.747221	kegg_pathway_73
Gastric acid secretion	kegg	0.288513	0.749377	kegg_pathway_132
Glioma	kegg	0.288513	0.749377	kegg_pathway_183
Prostate cancer	kegg	0.287894	0.749841	kegg_pathway_184
Aldosterone synthesis and secretion	kegg	0.287894	0.749841	kegg_pathway_120
Progesterone-mediated oocyte maturation	kegg	0.287894	0.749841	kegg_pathway_109
Ascorbate and aldarate metabolism	kegg	0.274086	0.760267	kegg_pathway_246

Glycosphingolipid biosynthesis - lacto and neolacto series	kegg	0.274086	0.760267	kegg_pathway_300
Olfactory transduction	kegg	0.271309	0.762381	kegg_pathway_100
Platelet activation	kegg	0.258868	0.771925	kegg_pathway_71
Phototransduction	kegg	0.257942	0.77264	kegg_pathway_102
Butanoate metabolism	kegg	0.257942	0.77264	kegg_pathway_306
Hippo signaling pathway - multiple species	kegg	0.257942	0.77264	kegg_pathway_212
Regulation of lipolysis in adipocytes	kegg	0.244759	0.782893	kegg_pathway_118
Linoleic acid metabolism	kegg	0.242866	0.784377	kegg_pathway_297
Asthma	kegg	0.242866	0.784377	kegg_pathway_195
Phagosome	kegg	0.227651	0.796402	kegg_pathway_45
Pyrimidine metabolism	kegg	0.223846	0.799438	kegg_pathway_259
beta-Alanine metabolism	kegg	0.215586	0.806069	kegg_pathway_272
Propanoate metabolism	kegg	0.203238	0.816084	kegg_pathway_305
Cushing syndrome	kegg	0.203093	0.816202	kegg_pathway_235
Proteoglycans in cancer	kegg	0.199226	0.819365	kegg_pathway_177
Insulin secretion	kegg	0.190397	0.826631	kegg_pathway_106
Glycerolipid metabolism	kegg	0.187282	0.82921	kegg_pathway_291
SNARE interactions in vesicular transport	kegg	0.180812	0.834592	kegg_pathway_40
Cholinergic synapse	kegg	0.179876	0.835374	kegg_pathway_95
Serotonergic synapse	kegg	0.179876	0.835374	kegg_pathway_96
Prion diseases	kegg	0.170626	0.843137	kegg_pathway_144
JAK-STAT signaling pathway	kegg	0.161066	0.851236	kegg_pathway_78
Starch and sucrose metabolism	kegg	0.161059	0.851242	kegg_pathway_279
DNA replication	kegg	0.161059	0.851242	kegg_pathway_9
Alanine, aspartate and glutamate metabolism	kegg	0.161059	0.851242	kegg_pathway_260

Central carbon metabolism in cancer	kegg	0.156715	0.854948	kegg_pathway_193
Cortisol synthesis and secretion	kegg	0.156715	0.854948	kegg_pathway_233
cAMP signaling pathway	kegg	0.149778	0.860899	kegg_pathway_26
cGMP-PKG signaling pathway	kegg	0.14747	0.862888	kegg_pathway_25
Influenza A	kegg	0.14747	0.862888	kegg_pathway_169
Drug metabolism - cytochrome P450	kegg	0.137108	0.871876	kegg_pathway_323
Huntington disease	kegg	0.133547	0.874986	kegg_pathway_143
Renin secretion	kegg	0.131131	0.877103	kegg_pathway_119
PI3K-Akt signaling pathway	kegg	0.127908	0.879934	kegg_pathway_48
Phospholipase D signaling pathway	kegg	0.122416	0.88478	kegg_pathway_207
Type I diabetes mellitus	kegg	0.121137	0.885913	kegg_pathway_124
Bile secretion	kegg	0.114707	0.891627	kegg_pathway_137
Bacterial invasion of epithelial cells	kegg	0.109699	0.896104	kegg_pathway_150
Viral carcinogenesis	kegg	0.10644	0.899029	kegg_pathway_175
Neuroactive ligand-receptor interaction	kegg	0.10624	0.899209	kegg_pathway_34
Glucagon signaling pathway	kegg	0.102873	0.902242	kegg_pathway_117
Carbohydrate digestion and absorption	kegg	0.102349	0.902714	kegg_pathway_134
Vasopressin-regulated water reabsorption	kegg	0.102349	0.902714	kegg_pathway_128
Relaxin signaling pathway	kegg	0.0963105	0.908182	kegg_pathway_55
Vascular smooth muscle contraction	kegg	0.089719	0.914188	kegg_pathway_53
Parathyroid hormone synthesis, secretion and action	kegg	0.0879824	0.915777	kegg_pathway_237
Drug metabolism - other enzymes	kegg	0.0877204	0.916017	kegg_pathway_324
Chemical carcinogenesis	kegg	0.0766813	0.926185	kegg_pathway_176
Mineral absorption	kegg	0.069423	0.932932	kegg_pathway_139
Parkinson disease	kegg	0.0626555	0.939267	kegg_pathway_141

Alzheimer disease	kegg	0.0573008	0.94431	kegg_pathway_140
Viral myocarditis	kegg	0.0499742	0.951254	kegg_pathway_206
Ribosome	kegg	0.0450612	0.955939	kegg_pathway_3
Steroid hormone biosynthesis	kegg	0.042441	0.958447	kegg_pathway_254
Oxytocin signaling pathway	kegg	0.0418433	0.95902	kegg_pathway_116
Oocyte meiosis	kegg	0.0412606	0.959579	kegg_pathway_36
Amoebiasis	kegg	0.0389415	0.961807	kegg_pathway_163
Natural killer cell mediated cytotoxicity	kegg	0.0380456	0.962669	kegg_pathway_80
Circadian entrainment	kegg	0.0372087	0.963475	kegg_pathway_89
Mitophagy - animal	kegg	0.03236	0.968158	kegg_pathway_223
Melanogenesis	kegg	0.0310016	0.969474	kegg_pathway_111
Staphylococcus aureus infection	kegg	0.0306551	0.96981	kegg_pathway_164
Long-term potentiation	kegg	0.0290417	0.971376	kegg_pathway_90
Renal cell carcinoma	kegg	0.0275141	0.972861	kegg_pathway_180
Apelin signaling pathway	kegg	0.0252563	0.97506	kegg_pathway_222
Focal adhesion	kegg	0.022307	0.97794	kegg_pathway_63
Cell adhesion molecules (CAMs)	kegg	0.0196886	0.980504	kegg_pathway_65
Complement and coagulation cascades	kegg	0.0152111	0.984904	kegg_pathway_70
Hypertrophic cardiomyopathy (HCM)	kegg	0.0110195	0.989041	kegg_pathway_203
Chemokine signaling pathway	kegg	0.0100837	0.989967	kegg_pathway_28
Dilated cardiomyopathy (DCM)	kegg	0.0079858	0.992046	kegg_pathway_205
Alcoholism	kegg	0.00403613	0.995972	kegg_pathway_149
Cytokine-cytokine receptor interaction	kegg	0.00177357	0.998228	kegg_pathway_27
Systemic lupus erythematosus	kegg	0.000933436	0.999067	kegg_pathway_198

Supplementary Table S5. The down-regulated genes involved in apoptosis pathway.

Gene Symbol	Function	p-value(PsA vs. HC)	Ratio(PsA vs. HC)	Fold-Change(PsA vs. HC)
ATM	Apoptosis	0.0182896	0.84475	Down-regulated in PsA
NFKB1	Apoptosis	0.00639143	0.777525	Down-regulated in PsA
BIRC2	Apoptosis	0.00755812	0.738397	Down-regulated in PsA
IKBKB	Apoptosis	0.0471825	0.646689	Down-regulated in PsA
TUBA8	Apoptosis	0.0227001	0.903879	Down-regulated in PsA
CAPN1	Apoptosis	0.0447247	0.935332	Down-regulated in PsA
CASP10	Apoptosis	0.0156491	0.815481	Down-regulated in PsA
CASP7	Apoptosis	0.00662208	0.918258	Down-regulated in PsA
CTSL	Apoptosis	0.0217646	0.601047	Down-regulated in PsA
CTSD	Apoptosis	0.0107855	0.919197	Down-regulated in PsA
MAPK10	Apoptosis	0.00752709	0.935478	Down-regulated in PsA
HRK	Apoptosis	0.0448435	0.910863	Down-regulated in PsA
FADD	Apoptosis	0.00357763	0.803587	Down-regulated in PsA
DFFA	Apoptosis	0.0418897	0.703108	Down-regulated in PsA
